# Can primary health care mitigate the effects of economic crises on child health in Latin America? An integrated multicountry evaluation and forecasting analysis

**DOI:** 10.1016/S2214-109X(24)00094-9

**Published:** 2024-05-16

**Authors:** Ana L Moncayo, Daniella Medeiros Cavalcanti, José Alejandro Ordoñez, Cristina Almeida, Juan Felipe Perdomo, Daniela Zuluaga, Alejandro Zamudio Sosa, Philipp Hessel, Carlos Chivardi, Davide Rasella

**Affiliations:** a Centro de Investigación para la Salud en América Latina (CISeAL), Pontificia Universidad Católica del Ecuador, Quito, Ecuador; a Institute of Collective Health (ISC), Federal University of Bahia, Bahia, Brazil; a Alberto Lleras Camargo School of Government, Universidad de los Andes, Bogotá, Colombia; a Health Research Consortium (CICIDAT), Cuernavaca, Mexico; a Swiss Tropical and Public Health Institute, Department of Public Health and Epidemiology, Basel, Switzerland; a Centre for Health Economics, University of York, York, UK; a Institute for Global Health (ISGlobal), Hospital Clinic-Universitat de Barcelona, Barcelona, Spain

## Abstract

**Background:**

Latin American and Caribbean countries are dealing with the combined challenges of pandemic-induced socicoeconomic stress and increasing public debt, potentially leading to reductions in welfare and health-care services, including primary care. We aimed to evaluate the impact of primary health-care coverage on child mortality in Latin America over the past two decades and to forecast the potential effects of primary health-care mitigation during the current economic crisis.

**Methods:**

This multicountry study integrated retrospective impact evaluations in Brazil, Colombia, Ecuador, and Mexico from 2000 to 2019 with forecasting models covering up to 2030. We estimated the impact of coverage of primary health care on mortality rates in children younger than 5 years (hereafter referred to as under-5 mortality) across different age groups and causes of death, adjusting for all relevant demographic, socioeconomic, and health-care factors, with fixed-effects multivariable negative binomial models in 5647 municipalities with an adequate quality of vital statistics. We also performed several sensitivity and triangulation analyses. We integrated previous longitudinal datasets with validated dynamic microsimulation models and projected trends in under-5 mortality rates under alternative policy response scenarios until 2030.

**Findings:**

High primary health-care coverage was associated with substantial reductions in post-neonatal mortality rates (rate ratio [RR] 0·72, 95% CI 0·71–0·74), toddler (ie, aged between 1 year and <5 years) mortality rates (0·75, 0·73–0·76), and under-5 mortality rates (0·81, 0·80–0·82), preventing 305 890 (95% CI 251 826–360 517) deaths of children younger than 5 years over the period 2000–19. High primary health-care coverage was also associated with lower under-5 mortality rates from nutritional deficiencies (RR 0·55, 95% CI 0·52–0·58), anaemia (0·64, 0·57–0·72), vaccine-preventable and vaccine-sensitive conditions (0·70, 0·68–0·72), and infectious gastroenteritis (0·78, 0·73–0·84). Considering a scenario of moderate economic crisis, a mitigation response strategy implemented in the period 2020–30 that increases primary health-care coverage could reduce the under-5 mortality rate by up to 23% (RR 0·77, 95% CI 0·72–0·84) when compared with a fiscal austerity response, and this strategy would avoid 142 285 (95% CI 120 217–164 378) child deaths by 2030 in Brazil, Colombia, Ecuador, and Mexico.

**Interpretation:**

The improvement in primary health-care coverage in Brazil, Colombia, Ecuador, and Mexico over the past two decades has substantially contributed to improving child survival. Expansion of primary health-care coverage should be considered an effective strategy to mitigate the health effects of the current economic crisis and to achieve Sustainable Development Goals related to child health.

**Funding:**

UK Medical Research Council.

**Translations:**

For the Spanish and Portuguese translations of the abstract see Supplementary Materials section.

## Introduction

The Declaration of Astana in 2018 emphasised the importance of health systems based on strong primary health care, which is essential for achieving universal health coverage and should be an integral part of coordinated multisectoral actions addressing economic and social determinants of health.^1^ According to this declaration, there is an urgent need to evaluate the effectiveness of primary health-care strategies in low-income and middle-income countries (LMICs) and how they can be developed, strengthened, and made more comprehensive, effective, and sustainable—especially in terms of achieving Sustainable Development Goals (SDGs).^1^ There is a growing consensus that health systems with greater primary health-care coverage respond better to local health needs and provide comprehensive services more efficiently.^2^ Several studies indicate that countries with well developed primary health care have better and more equitable health outcomes and provide stronger financial protection to their citizens.^3^

Latin American and Caribbean countries are among those that have suffered—and continue to suffer—the most from the socioeconomic consequences of the COVID-19 pandemic, the global inflationary surge, and widespread economic instabilities.^4^ Moreover, they are among the LMICs that have increased their public debt and might be forced to implement fiscal austerity measures,^5^ reducing the budget for welfare state and health-care services, including primary health care.

**Research in context**

**Evidence before this study**

We searched PubMed for published studies with the terms “primary health care” [MeSH Terms] OR “primary care” [MeSH Terms] AND “child mortality” OR “infant mortality”. The last search was conducted on Feb 20, 2023, and results were not limited by language. The reference lists of selected papers were also examined. Our search produced few studies on the effect of primary health care on child mortality in low-income and middle-income countries, most of which were conducted in Brazil. Moreover, most studies were retrospective, covered short periods of time, were country-specific, or were even limited to particular areas within a country. No studies evaluated the effectiveness of primary health care implementation strategies over long periods of time and in multiple countries or the effect of these implementation strategies on a wide number of causes of child mortality and different child age groups.

**Added value of this study**

To our knowledge, this is the first multicountry study that uses retrospective models to evaluate the impact of primary health care coverage on a wide range of causes of death in childhood over a 20-year period. In addition, this is the first study to forecast the effects of various policy implementation scenarios on child mortality up to 2030, integrating the datasets and parameters obtained from the retrospective impact evaluations.

**Implications of all the available evidence**

Primary health-care coverage was associated with reduced child mortality across different age groups and causes of death. The primary health-care effect was greatest for deaths related to nutritional deficiencies and anaemia. Evidence also indicates that reductions in public health-care coverage due to austerity measures during economic crises could be responsible for a substantial number of preventable child deaths in the coming years, and, if implemented, are likely to prevent Latin American countries from achieving Sustainable Development Goals related to child mortality.

The Latin American and Caribbean region is particularly interesting for evaluating primary health-care policies because several Latin American and Caribbean countries started reforming their health-care systems in the 1990s by developing frameworks to track improvements in care quality, enhancing primary health care, decentralising health governance, bolstering regulatory measures, and increasing effectiveness.^6^ In the Latin American and Caribbean region, Brazil, Colombia, Ecuador, and Mexico (which represent 63% of the Latin American and Caribbean population) are among the countries that have implemented strategies for nationwide delivery of public primary health care. Brazil has one of the largest primary health-care programmes in the world.^2^ The Estratégia Saúde da Família (ESF; Family Health Strategy) is the primary vehicle for achieving universal health coverage within Brazil's Unified Health System and its coverage has expanded from 6·6% in 1998 to 62·6% in 2019.^7^ The ESF encompasses key principles of primary health care including community-based care, multidisciplinary teams, and a focus on health prevention and promotion.^8^ In 2004, Mexico established a health protection system, known as Seguro Popular de Salud (SPS), which covers people working in the informal sector or people who are socioeconomically vulnerable. SPS has gradually expanded to include 51 million people by 2019 (approximately 41% of Mexico's population).^5^ Colombia's subsidised health-care scheme was introduced in 1993 (known as the Régimen Subsidiado [RS]) for people with low income or people working in the informal sector. Its coverage increased from 15·7% in 1993 to 45·3% in 2019.^9^ In 2008, Ecuador reformed its constitution to consider health care as a right and built a health-care model, known as the Modelo de Atención Integral de Salud Familiar, Comunitario e Intercultural (MAIS-FCI), based on primary health-care strategy with a focus on prevention and health promotion, and including community-based care and multidisciplinary health teams. Under this model, use of health-care services increased by 300% during the 2008–16 period. More details on each primary health-care strategy are available in appendix 3 (pp 12–14).^10^ As a consequence, primary health care has been strengthened but implemented in different ways in Brazil, Colombia, Ecuador, and Mexico. In this study, we consider the ESF, SPS, RS, and MAIS-FCI nationwide strategies for delivering public primary health care inspired by the main principles of the Declarations of Alma-Ata and Astana:^11^, ^12^ promoting universal access to health services for everyone; fostering comprehensive care; and including health promotion, health prevention, treatment, rehabilitation, and palliation with a health equity prospective and a particular focus on providing health-care access to the most vulnerable populations. A more complete description is provided in appendix 3 (pp 2–13).^1^

The objective of this study was to comprehensively evaluate the effects of strategies that have been implemented to expand primary health-care coverage over the past two decades on mortality among children younger than 5 years (hereafter referred to as under-5 mortality; overall, for different causes, and for different age subgroups) in Brazil, Colombia, Ecuador, and Mexico. Moreover, we aimed to forecast the mitigation effects on child mortality of a potential expansion in primary health care versus the effects of a reduction in primary health care due to fiscal austerity during the current economic crisis and beyond, until 2030.

## Methods

### Study design

We performed a multicountry study with a mixed ecological design, which combines a time-trend design with a multiple-group ecological design, and municipalities as our units of analysis. We created a longitudinal dataset (2000–19) linking different demographic, socioeconomic, and health data from Brazil, Colombia, Ecuador, and Mexico from several resources (appendix 3 pp 13–14) We used a validated multidimensional criterion to select only municipalities with adequate vital information (appendix 3 pp 20–22).^13^ Of the 8332 municipalities in Brazil, Colombia, Ecuador, and Mexico, 5647 met the inclusion criteria for adequacy of vital information. The exclusion of municipalities without adequate data is important for strengthening the internal validity of this study and reducing any possible bias due to changes in the quality of the death notification system. Moreover, the selected municipalities represent 86% of the entire population of these four countries. We carried out tests that showed inclusion of these data-poor municipalities in our analyses does not significantly change the results (appendix 3 p 23).

We defined the following dependent variables: under-5 mortality rate (number of deaths of children younger than 5 years per 1000 livebirths); toddler mortality rate (number of deaths of children aged between 1 year and <5 years per 1000 livebirths); infant mortality rate (number of deaths of children younger than 1 year per 1000 livebirths); post-neonatal mortality rate (number of deaths of children aged between 28 days and <1 year per 1000 livebirths; neonatal mortality rate (number of deaths of children younger than 28 days per 1000 livebirths; and cause-specific under-5 mortality rate (number of deaths of children younger than 5 years resulting from specific causes per 1000 livebirths). We selected specific causes of death from the Brazilian list of primary care-sensitive conditions created by the Ministry of Health in 2008,^14^ which has been used in Brazilian settings as a measure of primary health-care effectiveness and covers immunopreventable diseases and vaccine-sensitive conditions, infectious gastroenteritis and its complications, anaemia, nutritional deficiencies, and bacterial pneumonia, among others.^14^

The main exposure variable was populational primary health-care coverage, which was calculated for each country with country-specific parameters, conditions, and strategies to deliver primary health care. In Brazil and Ecuador, we multiplied the number of primary health-care units or teams by the expected number of individuals living in their catchment areas, as done in previous studies.^15^, ^16^ In Brazil, populational primary health-care coverage was derived by dividing the number of people in ESF catchment areas (number of family health teams × 3500) by the total population of the municipality.^12^, ^16^ In Ecuador, coverage was derived by dividing the number of people in primary health-care catchment areas (number of general and family physicians in primary care facilities × 2500 in rural areas and × 4000 in urban areas)^17^ by the total population of the municipality. In Mexico and Colombia, we used the number of individuals enrolled into programmes or strategies to deliver primary health care for the most vulnerable populations. In Mexico, the number of people enrolled into the SPS was divided by the total population of the municipality. In Colombia, the number of people enrolled into the RS was divided by the total population of the municipality. As in previous studies,^12^, ^15^, ^18^ to evaluate a dose–response relationship for an intervention, we categorised primary health-care coverage in quartiles for all countries: low (<34%); intermediate (≥34% to <70%); high (≥70% to <100%); and consolidate (100%). Finally, we also included mortality from external causes (International Classification of Diseases [10th revision] codes V01–99, which only include transport accidents) as a negative control variable, because primary health care was not expected to exert any effect on these causes.^15^

On the basis of a literature review,^15^, ^18^ a set of covariates—determinants of child mortality and potential confounders of the primary health-care association—was used to adjust the models. These covariates were stratified according to their median value over the period for: the Gini index (0 for ≤46·5% and 1 for >46·5%); the poverty rate index (0 for ≤27·0% and 1 for >27·0%); illiteracy (0 for ≤12·6% and 1 for >12·6%); the percentage of households with adequate sewage (0 for ≤38·5% and 1 for >38·5%) and clean, piped water (0 for ≤71·5% and 1 for >71·5%); the number of physicians per 1000 population (0 for ≤0·6 and 1 for >0·6); and the number of hospital beds per 1000 population (0 for ≤1·7 and 1 for >1·7). Time dummy variables were also included in the models to control for the creation of the SPS in 2004,^19^ health reform in Ecuador in 2008,^10^ and major economic crises in Latin American and Caribbean countries over the past two decades (ie, the 2007–08 subprime mortgage crisis, the 2015 trade crisis, and the austerity measures introduced in 2018 by the Brazilian Government).^20^ Moreover, fixed terms were used to adjust for other time-invariant, unobserved variables with potentially confounding effects. The data used in this study were collected from different data resources in national information systems (see appendix 3 pp 13–14). We estimated some covariates through linear interpolation or exponential decay extrapolation, as in previous studies.^12^, ^15^, ^18^ These methods are described in appendix 3 (pp 15–19).

### Retrospective analysis

The effects of primary health-care strategies on child mortality were evaluated with conditional negative binomial regression models for panel data with fixed-effects specification (with municipalities as the units of analysis and observations repeated over time). Fixed-effect models include a term to control unmeasured time-invariant municipality characteristics, such as geographical, cultural, or socioeconomic factors, allowing for unbiased effect estimates. The model type used is a consolidated model for impact evaluations with aggregate-level panel data, and our choice of this model was confirmed with the Hausmann test.^11^, ^12^, ^18^ The negative binomial distribution was chosen because municipal mortality rates are characterised by overdispersion and the combination of this distribution with a fixed-effect specification can control selection biases related to the occurrence of death and the intensity of child mortality. We calculated mortality rate ratios, both crude and adjusted for a set of demographics and social, economic, and health-care-related covariates. Moreover, we estimated how many child deaths have been averted in Brazil, Colombia, Ecuador, and Mexico during the past two decades (2000–19) due to the implementation of primary health-care programmes by comparing the real primary health-care coverage with a hypothetic scenario with 0% primary health-care coverage in all countries over the same period (see appendix 3 p 53).

A wide range of sensitivity analyses were performed to show the robustness of the results (appendix 3 pp 27–52), including fitting Poisson regressions instead of negative binomial regressions; testing different sets of time variables; fitting the models with all municipalities in Brazil, Colombia, Ecuador, and Mexico; testing random instead of fixed-effect models; including continuous primary health-care coverage variables; and testing different categorisations for primary health-care coverage. We also estimated an alternative correction for potential selection biases following the Heckman (1979) model.^21^ Moreover, triangulation analyses were conducted with difference-in-differences with propensity score matching (appendix 3 pp 27–28).^22^ The software Stata (version 15) was used for data processing and analysis.

### Forecast analysis

We used validated municipal-level microsimulation models to predict the consequences of the current economic crisis and the mitigating effects of modifications of primary health-care coverage.^20^ We developed the microsimulation in two steps, as in previous studies.^20^ The first step was to create a 2020–30 synthetic cohort of all municipalities in Brazil, Colombia, Ecuador, and Mexico by extrapolating and modelling the independent variables as an extension of the retrospective cohort (ie, the 2000–19 dataset). The second step was to predict mortality rates from 2020 to 2030 with these independent variables as inputs in the same multivariate regression models used in the retrospective analysis.

We simulated three economic crisis scenarios based on changes in poverty rates (established from household surveys in Brazil, Colombia, Ecuador, and Mexico), which vary in the severity and duration of their poverty rates increases (2020–22 for the shorter economic crisis scenario, 2020–24 for the moderate scenario, and 2020–26 for the longer scenario), but are all followed by gradual decreases in poverty rates until 2030 (as in previous studies).^20^ In response to the economic crisis, three policy response scenarios (the maintenance, baseline, and fiscal austerity response scenarios) were defined as potential changes in primary health-care coverage, with functions and parameters from verified models of previous studies based in Brazil.^23^ In the maintenance response scenario, the increase in primary health-care coverage was proportional to the poverty rate increase during the economic crisis, with a subsequent reduction in primary health-care coverage after the estimated end of the crisis. The baseline response scenario depicts a situation in which current trends are maintained, gradually reducing the budget for all welfare state services in accordance with published estimates.^20^, ^23^ In the fiscal austerity response scenario, primary health-care coverage falls in line with the estimated decline in public spending on the welfare state—excluding some cash transfer programmes—from 2014 to 2019, and with potential fiscal austerity measures in LMICs.^24^ A detailed description of the equations and parameters for each economic crisis and primary health-care policy scenario, together with the complete modelling process, is provided in appendix 3 (pp 54–58).

All modelling techniques, including calibration, validation, and model equations, are detailed in appendix 3 according to the international model reporting guidelines (The International Society for Pharmacoeconomics and Outcomes Research and Society for Medical Decision Making).^25^ The forecasting analysis was performed in R (version 4.1.2).

### Role of the funding source

The funder of the study had no role in the study design, data collection and analysis, decision to publish, or preparation of the manuscript.

## Results

Descriptive statistics show a decrease in the average municipal mortality rates between 2000 and 2019 in Brazil, Colombia, Ecuador, and Mexico. The under-5 mortality rate decreased by 36·4% during 2000–10, and by 5·6% in 2010–19 for the municipalities under study. The largest reductions were observed in the post-neonatal and toddler mortality rates (which decreased by 45·3% and 42·8%, respectively, over the 2000–19 period; table 1).

**Table 1:** Mortality rates, primary health-care coverage, and control variables for selected municipalities in Brazil, Colombia, Ecuador, and Mexico (N=5647)

	**2000**	**2010**	**2019**	**Percentage change, 2000–19**
**Mortality rate for children younger than 5 years**				
Overall	24·89 (17·92)	15·84 (12·47)	14·95 (12·91)	−39·94%
Neonatal mortality	13·36 (13·28)	9·70 (9·72)	9·86 (11·56)	−26·20%
Post-neonatal mortality	8·75 (14·36)	4·74 (6·36)	4·79 (7·74)	−45·26%
Infant mortality	21·10 (16·07)	13·48 (10·98)	13·26 (11·57)	−37·16%
Toddler* mortality	4·00 (5·78)	2·67 (4·69)	2·29 (4·60)	−42·75%
Primary health-care coverage (%)	22·48% (32·01)	71·04% (29·96)	76·86% (26·32)	241·90%
**Control variables**				
Proportion of households with adequate sanitation (%)	29·59% (25·97)	40·83% (30·14)	46·01% (31·86)	55·49%
Proportion of households with adequate clean water (%)	63·24% (24·18)	71·58% (21·78)	77·49% (21·73)	22·53%
Proportion of individuals older than 15 years who are illiterate (%)	16·57% (10·21)	12·36% (7·93)	9·00% (6·53)	−45·68%
Proportion of individuals living in poverty (%)	38·89% (21·63)	26·37% (19·99)	18·87% (18·28)	−51·48%
Gini index (%)	51·17% (7·54)	45·90% (6·99)	43·78% (9·09)	−14·44%
Hospital bed rate (per 1000 inhabitants)	1·91 (2·43)	1·71 (2·02)	1·61 (1·99)	−15·71%
Physician rate (per 1000 inhabitants)	0·57 (0·57)	0·61 (0·61)	0·83 (0·94)	45·61%

Data are mean (SD). Mortality rates were calculated per 1000 livebirths.

* Aged between 1 year and <5 years.

Table 2 shows both the non-adjusted and adjusted effects of primary health-care coverage on the mortality rates of different age groups. All models show a negative association between primary health-care coverage and child mortality outcomes. In multivariate analysis, a dose–response effect of primary health care was shown for the under-5 mortality rate, the post-neonatal mortality rate, and the toddler mortality rate, with a reduction of 18·6% (rate ratio [RR] 0·81, 95% CI 0·80–0·82), 27·6% (0·72, 0·71–0·74), and 25·4% (0·75, 0·73–0·76), respectively, when consolidate coverage is achieved, compared with low coverage. On the basis of these models, we estimated that the number of child deaths prevented by the implementation of primary health care programmes over the past two decades in Brazil, Colombia, Ecuador, and Mexico was 305 890 deaths (95% CI 251 826–360 517; appendix 3 p 53).

**Table 2:** Fixed-effect negative binomial model results for the association between primary health-care coverage and age-specific mortality rates in Brazil, Colombia, Ecuador, and Mexico between 2000 and 2019

	**Child (younger than 5 years) mortality (109 080 observations, 5576 municipalities)**	**Toddler (aged between 1 year and <5 years) mortality (103 234 observations, 5362 municipalities)**	**Infant (younger than 1 year) mortality (106 544 observations, 5575 municipalities)**	**Post-neonatal (aged 28 days to <1 year) mortality (105 699 observations, 5526 municipalities)**	**Neonatal (younger than 28 days) mortality (105 170 observations, 5572 municipalities)**
**Primary health-care coverage**					
Low (less than 34%)	1·000 (ref)	1·000 (ref)	1·000 (ref)	1·000 (ref)	1·000 (ref)
Intermediate (≥34% to <70%)	0·888* (0·883–0·893)	0·900* (0·889–0·911)	0·866* (0·861–0·872)	0·876* (0·868–0·884)	0·890* (0·884–0·896)
High (≥70% to <100%)	0·843* (0·837–0·851)	0·807* (0·793–0·821)	0·863* (0·855–0·871)	0·817* (0·806–0·828)	0·865* (0·856–0·874)
Consolidate (100%)	0·814* (0·805–0·822)	0·746* (0·730–0·763)	0·864* (0·853–0·875)	0·724* (0·711–0·737)	0·866* (0·854–0·879)
**Control variables**					
Proportion of households with adequate sanitation	0·901* (0·893–0·909)	0·914* (0·896–0·932)	0·919* (0·909–0·928)	0·868* (0·855–0·882)	0·918* (0·908–0·928)
Proportion of households with adequate clean water	0·991† (0·982–1·000)	1·006 (0·987–1·026)	0·960* (0·950–0·970)	0·956* (0·942–0·971)	1·004 (0·992–1·015)
Proportion of individuals older than 15 years who are illiterate	1·094* (1·083–1·105)	1·114* (1·091–1·1·138)	1·129* (1·117–1·142)	1·089* (1·071–1·107)	1·078* (1·065–1·091)
Proportion of individuals living in poverty	1·252* (1·240–1·263)	1·296* (1·273–1·320)	1·306* (1·294–1·318)	1·266* (1·248–1·284)	1·247* (1·234–1·260)
Gini index	1·099* (1·091–1·107)	1·083* (1·065–1·102)	1·057* (1·048–1·066)	1·107* (1·093–1·121)	1·089* (1·079–1·100)
Hospital bed rate	0·993 (0·982–1·005)	1·002 (0·978–1·027)	0·953* (0·941–0·965)	1·003 (0·984–1·022)	0·985† (0·971–1·000)
Physician rate	0·993‡ (0·984–1·002)	0·974* (0·955–0·993)	0·951* (0·941–0·961)	0·986‡ (0·971–1·001)	1·003 (0·992–1·015)

Data are rate ratios (95% CI) unless otherwise specified. Time dummy variables were also included in the models to control for the creation of the Seguro Popular de Salud in 2004, health reform in Ecuador in 2008, and major economic crises in Latin American and Caribbean countries over the past two decades (ie, the 2007–08 subprime mortgage crisis, the 2015 trade crisis, and the austerity measures introduced in 2018 by the Brazilian Government).

* p<0·01.

† p<0·05.

‡ p<0·1.

The adjusted associations between primary health-care programme coverage and the under-5 mortality rate associated with selected primary care-sensitive conditions are shown in table 3. Of all the causes included in the analysis, primary health-care coverage shows effects on vaccine-preventable and vaccine-sensitive conditions, anaemia, infectious gastroenteritis, bacterial pneumonias, and skin and subcutaneous infection. The strongest reductions (in municipalities with consolidate coverage) were observed for the under-5 mortality rates caused by nutritional deficiencies (reduction of 45·3%, RR 0·55, 95% CI 0·52–0·58) and anaemia (reduction of 36·0%, 0·64, 0·57–0·72). Reductions in under-5 mortality rates from vaccine-preventable and vaccine-sensitive conditions and infectious gastroenteritis were 30·1% (0·70, 0·68–0·72) and 21·7% (0·78, 0·73–0·84), respectively. No level of primary health-care coverage had any effect on mortality rates caused by external causes, which was used as a negative control. All sensitivity and triangulation analyses confirmed the robustness and the high degree of confidence in the results. These analyses are presented, together with complementary tests, in appendix 3 (pp 27–52).

**Table 3:** Fixed-effect negative binomial model results for the association between primary health-care coverage and the mortality rate for children younger than 5 years due to primary care-sensitive conditions in Brazil, Colombia, Ecuador, and Mexico between 2000 and 2019

	**Vaccine-preventable and vaccine-sensitive conditions (86 493 observations, 4797 municipalities)**	**Infectious gastroenteritis and complications (69 631 observations, 3749 municipalities)**	**Anaemia (38 675 observations, 2036 municipalities)**	**Nutritional deficiencies (72 832 observations, 3926 municipalities)**	**Bacterial pneumonias (77 596 observations, 4221 municipalities)**	**Skin and subcutaneous infection (93 665 observations, 5523 municipalities)**	**External causes (32 812 observations, 1991 municipalities)**
Low (less than 34%)	1·000 (ref)	1·000 (ref)	1·000 (ref)	1·000 (ref)	1·000 (ref)	1·000 (ref)	1·000 (ref)
Intermediate (≥34 to <70%)	0·813* (0·798–0·828)	0·956* (0·926–0·988)	0·897* (0·836–0·962)	0·779* (0·755–0·805)	0·870* (0·846–0·895)	0·894* (0·887–0·901)	0·936 (0·871–1·100)
High (≥70% to <100%)	0·750* (0·732–0·769)	0·825* (0·786–0·867)	0·749* (0·680–0·824)	0·633* (0·606–0·662)	0·775* (0·744–0·806)	0·851* (0·842–0·861)	0·864 (0·782–1·002)
Consolidate (100%)	0·699* (0·678–0·721)	0·783* (0·732–0·837)	0·640* (0·571–0·716)	0·547* (0·517–0·579)	0·809* (0·766–0·854)	0·852* (0·839–0·865)	0·974 (0·856–1·108)

Data are rate ratios (95% CI) unless otherwise specified. Time dummy variables were included in the models to control for specific years of economic crisis (2007, 2008, 2015, and 2018) and for specific years related to primary health-care programmes (2004–08). Models were also adjusted by the following control variables: adequate sanitation, adequate clean water, illiteracy, poverty, GINI index, hospital bed rate, and physicians rate.

* p<0·01.

The figure shows projections of the effects of a moderate economic crisis from 2020 to 2030 and three alternative policy responses in terms of primary health-care coverage on change in child mortality rate; the projections for other economic scenarios are shown in appendix 3 (p 56). Under a mitigation policy response scenario, the effect of the increase in poverty rates is completely mitigated by the increase in primary health-care coverage to maintenance response levels, and the trajectory of the under-5 mortality rate continuously declines until 2030. Under the baseline and fiscal austerity response scenarios, the under-5 mortality rate shows an initial increase due to the growth in poverty rates and the decrease in primary health-care coverage, followed by a more gradual reduction in mortality rate after the expected end of the economic crisis.

**Figure:**
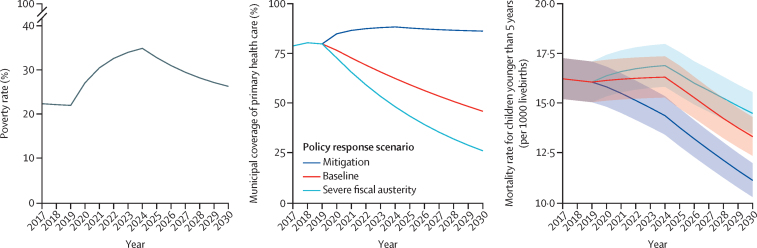
Predicted poverty rates under the moderate crisis scenario, alternative responses of primary health-care coverage, and related child mortality rate predictions for Brazil, Colombia, Ecuador, and Mexico up to 2030

Table 4 shows the comparison between different primary health-care policy responses in terms of the under-5 mortality rate and avoidable deaths under the moderate economic crisis scenario over the period 2020–30 (see appendix 3 p 59 for other scenarios). In 2030, the under-5 mortality rate will be 16·7% lower (RR 0·83, 95% CI 0·79–0·88) if the maintenance policy is implemented than if the baseline implementation scenario is followed, averting 103 737 (95% CI 86 813–120 895) deaths of children younger than 5 years over the period 2020–30. The reduction in mortality was greater when we compared the mitigation versus fiscal austerity response scenarios (reduction of 23·0%, RR 0·77, 95% CI 0·72–0·84, in 2030; 142 285 [95% CI 120 217–164 378] averted deaths of children younger than 5 years if the mitigation response was adopted during the period 2020–30).

**Table 4:** Rate ratios and number of avoidable deaths under the moderate economic crisis scenario for comparisons between the maintenance, baseline, and austerity primary health-care coverage response scenarios in Brazil, Colombia, Ecuador, and Mexico from 2020 to 2030

	**Maintenance *vs* baseline**	**Maintenance *vs* fiscal austerity**
2020	0·942 (0·904–0·982)	0·938 (0·900–0·983)
2025	0·869 (0·828–0·911)	0·841 (0·798–0·894)
2030	0·833 (0·788–0·881)	0·770 (0·724–0·842)
Avoidable deaths	103 737 (86 813–120 895)	142 285 (120 217–164 378)

Data are rate ratios (95% CI) or n (95% CI).

## Discussion

The findings of this study show that primary health-care strategies had a substantial impact on childhood mortality over the past two decades in four large countries in Latin America, having prevented more than 300 000 child deaths between 2000 and 2019 in Brazil, Colombia, Ecuador, and Mexico. An additional 140 000 child deaths could potentially be averted if primary health care is further expanded (rather than reduced by austerity measures) by 2030. Primary health-care coverage had particularly strong effects in reducing post-neonatal mortality and mortality of children aged 1 year to younger than 5 years, and of childhood deaths from anaemia, nutritional deficiencies, infectious gastroenteritis, and vaccine-preventable conditions. This is the most comprehensive multicountry study on the effects of primary health-care coverage on child health, and it is unique in its integration of a two-decades-long retrospective impact evaluation with dynamic microsimulation models to forecast primary health-care mitigation effects during an economic crisis.

Previous studies in Brazil and Mexico over shorter periods have shown that primary health-care strategies could reduce child and infant mortality.^26^, ^27^ In this study, we found a stronger, dose–response effect on post-neonatal mortality and mortality for children aged between 1 year and 4 years than on other child mortality rates. This result is expected because primary health care has been shown to increase access to routine immunisation in these age groups, preventing infectious diseases that contribute to child mortality.^11^ In fact, one of the strongest effects of primary health-care coverage that we found was on reducing deaths from vaccine-preventable diseases and vaccine-sensitive conditions, and our complementary analyses (appendix 3 p 44) showed a dose–response effect of primary health care on increases of vaccination rates. Moreover, primary health care has been associated with better postnatal care in terms of offering support for breastfeeding, child growth control, and education on health and nutrition for mothers.^12^ Primary health care promotes important public health strategies that have been recognised as the most effective interventions to reduce childhood mortality due to nutritional conditions worldwide, such as oral rehydration therapy, exclusive breastfeeding, correct weaning practices, micronutrient supplementation, improved personal hygiene, and safer food preparation practices.^28^ In addition, primary health services are well placed to assess people's diets, screen for dietary risk factors, diagnose diet-related disease, and take appropriate action.^28^ In fact, our findings show a strong effect of primary health-care coverage on mortality of children younger than 5 years from nutritional deficiencies and anaemia. Primary health care also allows timely diagnosis and treatment of common childhood illnesses such as pneumonia, diarrhoea, and malaria—reducing geographical, economic, and cultural barriers to child health care and ultimately contributing to reduced child mortality.^29^ We found a strong impact of primary health-care coverage on mortality from infectious gastroenteritis and bacterial pneumonias. Similar results showing a strong effect of primary health care on mortality for diarrhoea and lower respiratory infections previously were reported in two studies in Brazil.^12^, ^15^ Primary health care can reduce child mortality by promoting health education and empowering families to make informed decisions about their children's health and wellbeing, often through the work and domiciliary visits of community health workers, physicians, and nurses (besides other educational activities).^18^ Moreover, primary health care encourages community engagement and empowerment, involving communities in health decision making and promoting community-based health services.^19^

The sustainability of health system investments to achieve and maintain primary health care and universal health care in LMICs has been affected by the current global economic crisis, which started with the COVID-19 pandemic and is continuing with the global consequences of the war in Ukraine, among other factors.^4^ Consequently, Latin American countries face a reduction in their economic growth, inflationary pressures, and declining value of their currencies. These countries have also experienced high levels of unemployment and poverty, increased inequality, and social exclusion.^23^ The most common political response to these economic downturns in Latin American and Caribbean countries has historically been the implementation of fiscal austerity measures to reduce public debt, and this response is often translated into the reduction of social protection and health-care services. For instance, Brazil's Unified Health System continues to be underfunded as a result of the federal health budget's stagnation, with only a 3·2% growth in public health spending over the past 10 years.^6^ Colombia has also been plagued by underfunding of its subsidised Plan Obligatorio de Salud and Mexico faced a 25% deficit in health spending in 2013.^6^ Ecuador also had stagnation in its health spending in the past few years and saw a decrease in spending during the COVID-19 pandemic.^10^ Our forecast analysis broadens the conclusions of other studies,^8^, ^26^ showing how in Latin American and Caribbean countries, the implementation of fiscal austerity measures affecting primary health care will have a substantial negative impact on child health outcomes, potentially causing a large number of preventable child deaths in the next decade. In the upcoming years, additional studies will be necessary to assess which specific characteristics of primary health-care strategies contribute to the ability to mitigate and demonstrate resilience in the face of socioeconomic crises.

The limitations of this study include the exclusive use of municipalities with an adequate quality of vital statistics (which was done to improve the internal validity of our analyses). These municipalities represent more than 85% of the total population in Brazil, Colombia, Ecuador, and Mexico because the municipalities with a lower or inadequate quality of vital information are also the smallest municipalities in terms of their populations. However, models that were fitted with data from all municipalities returned similar dose–response relationships and statistically significant effects to those of the models that excluded some municipal data. Another limitation of our study is its ecological design, as there is the possibility of ecological fallacy, wherein associations observed at an ecological level might not accurately represent associations at the individual level.

In conclusion, our results show the fundamental role of primary health-care strategies in reducing childhood mortality in four large countries in Latin America (and potentially in other LMICs over the past two decades). The effects of primary health-care coverage on poverty-related diseases and vaccine-preventable conditions are particularly apparent. Moreover, all our forecast scenarios show that a prompt expansion of primary health-care coverage, to protect growing numbers of socioeconomically vulnerable populations, could represent an effective policy to mitigate the adverse health impact of the current economic crises. Reductions in primary health-care coverage due to austerity measures, however, could be responsible for a large number of preventable child deaths in coming years and might prevent LMICs from achieving SDGs related to child health and child mortality.

## Data sharing

All data used for this study are available in the public domain. Details of all datasets are provided in appendix 3 (pp 4–5).

## Declaration of interests

We declare no competing interests.

## Supplementary Materials

Spanish translation of the abstract

Portuguese translation of the abstract
