# Supplementary material for: Can primary health care mitigate the effects of economic crises on child health in Latin America? An integrated multicountry evaluation and forecasting analysis
Source: Lancet Glob Health. 2024 May 16;12(6):e938–46. doi: 10.1016/S2214-109X(24)00094-9 (PMC11126366; doi:10.1016/S2214-109X(24)00094-9)
Supplement: Spanish translation of the abstract [file mmc1.pdf]

THE LANCET
Global Health

Supplementary appendix 1

This translation in Spanish was submitted by the authors and we reproduce it as supplied. It has not been peer reviewed. *The Lancet*'s editorial processes have only been applied to the original in English, which should serve as reference for this manuscript.

Los autores nos proporcionaron esta traducción al español y la reproducimos tal como nos fue entregada. No la hemos revisado. Los procesos editoriales de *The Lancet* se han aplicado únicamente al original en inglés, que debe servir de referencia para este manuscrito.

Supplement to: Moncayo AL, Medeiros Cavalcanti D, Ordoñez JA, et al. Can primary health care mitigate the effects of economic crises on child health in Latin America? An integrated multicountry evaluation and forecasting analysis. *Lancet Glob Health* 2024; **12:** e938–46.

¿Puede la atención primaria de salud mitigar los efectos de las crisis económicas en la salud infantil en América Latina? Una evaluación multicéntrica integrada con un análisis de pronóstico.

Resumen

Antecedentes Los países de América Latina y el Caribe están enfrentando los desafíos combinados del estrés socioeconómico inducido por la pandemia y el aumento de la deuda pública, lo que podría conducir a reducciones en la oferta de los servicios de bienestar y de cuidados de salud, incluyendo la atención primaria. Nuestro objetivo fue evaluar el impacto de la cobertura de atención primaria de salud en la mortalidad infantil en países de América Latina durante las últimas dos décadas y pronosticar los efectos potenciales de la mitigación de los cuidados primarios de salud durante la actual crisis económica.

Métodos Este estudio multicéntrico integró evaluaciones de impacto retrospectivas en Brasil, Colombia, Ecuador y México desde 2000 hasta 2019 con modelos de pronóstico hasta 2030. Estimamos el impacto de la cobertura de atención primaria de salud en las tasas de mortalidad en niños menores de 5 años (en adelante, mortalidad de menores de 5 años) en diferentes grupos de edad y causas de muerte, ajustando para todos los factores demográficos, socioeconómicos y de atención médica relevantes. Utilizamos modelos binomiales negativos de efectos fijos en 5647 municipios con una adecuada calidad de estadísticas vitales. También realizamos varios análisis de sensibilidad y triangulación. Integramos un conjunto de datos longitudinales con modelos validados de microsimulación dinámica y tendencias proyectadas en las tasas de mortalidad de menores de 5 años bajo diferentes escenarios de respuesta política hasta el 2030.

Hallazgos Una alta cobertura de atención primaria de salud se asoció con reducciones sustanciales en las tasas de mortalidad postneonatal (razón de tasas [RT] 0·72, IC del 95% 0·71–0·74), tasas de mortalidad de niños pequeños (es decir, de 1 a <5 años) (0·75, 0·73–0·76), y tasas de mortalidad infantil (0·81, 0·80–0·82), previniendo 305.890 (IC del 95% 251·826–360·517) muertes de niños menores de 5 años durante el período 2000–19. Una alta cobertura de atención primaria de salud también se asoció con tasas más bajas de mortalidad de menores de 5 años por deficiencias nutricionales (RT 0·55, IC del 95% 0·52–0·58), anemia (0·64, 0·57–0·72), condiciones prevenibles y sensibles a la vacuna (0·70, 0·68–0·72), y gastroenteritis infecciosa (0·78, 0·73–0·84). Considerando un escenario de crisis económica moderada, una estrategia de respuesta de mitigación implementada en el período 2020–30, que aumenta la cobertura de atención primaria de salud, podría reducir la tasa de mortalidad de menores de 5 años en hasta un 23% (RT 0·77, IC del 95% 0·72–0·84) en comparación con una respuesta de austeridad fiscal, y esta estrategia evitaría 142.285 (IC del 95% 120·217–164·378) muertes de niños para 2030 en Brasil, Colombia, Ecuador y México.

Interpretación La mejora en la cobertura de atención primaria de salud en Brasil, Colombia, Ecuador y México durante las últimas dos décadas ha contribuido de manera sustancial para mejorar la supervivencia infantil. La expansión de la cobertura de atención primaria de salud debería considerarse una estrategia efectiva para mitigar los efectos de la actual crisis económica en la salud y para alcanzar los Objetivos de Desarrollo Sostenible relacionados con la salud infantil.

Financiamiento UK Medical Research Council.
