# Supplementary material for: Can primary health care mitigate the effects of economic crises on child health in Latin America? An integrated multicountry evaluation and forecasting analysis
Source: Lancet Glob Health. 2024 May 16;12(6):e938–46. doi: 10.1016/S2214-109X(24)00094-9 (PMC11126366; doi:10.1016/S2214-109X(24)00094-9)
Supplement: Portuguese translation of the abstract [file mmc2.pdf]

THE LANCET
Global Health

Supplementary appendix 2

This translation in Portuguese was submitted by the authors and we reproduce it as supplied. It has not been peer reviewed. *The Lancet*'s editorial processes have only been applied to the original in English, which should serve as reference for this manuscript.

Esta tradução em português foi submetida pelos autores e nós não fizemos quaisquer alterações. Esta versão não foi revista por pares. O processo editorial do The Lancet só foi aplicado à versão original em inglês, que deve servir como referência para este artigo.

Supplement to: Moncayo AL, Medeiros Cavalcanti D, Ordoñez JA, et al. Can primary health care mitigate the effects of economic crises on child health in Latin America? An integrated multicountry evaluation and forecasting analysis. *Lancet Glob Health* 2024; **12:** e938–46.

Os cuidados da atenção primária de saúde podem mitigar os efeitos das crises econômicas na saúde infantil de países da América Latina? Uma avaliação muticêntrica integrada com análise de previsão

Resumo

Antecedentes Os países da América Latina e Caribe enfrentam múltiplos desafios induzidos pela pandemia – incluindo crises socioeconômicas e aumento da dívida pública – podendo levar a reduções na oferta de serviços de bem-estar e de cuidados de saúde, incluindo a atenção primária. Nosso objetivo é avaliar o impacto da cobertura da atenção primários de saúde na mortalidade infantil em países da América Latina nas últimas duas décadas; e prever os efeitos potenciais da mitigação dos cuidados primários de saúde durante a atual crise econômica.

Métodos Este estudo muticêntrico integrou avaliações de impacto retrospectivas de 2000 a 2019 no Brasil, Colômbia, Equador e México com modelos de previsão até 2030. Estimamos o impacto da cobertura da atenção primária à saúde nas taxas de mortalidade em crianças menores de 5 anos (doravante denominadas até a mortalidade de menores de 5 anos) em diferentes grupos etários e causas de morte, ajustando para todos os fatores demográficos, socioeconômicos e de saúde relevantes. Utilizamos modelos binomiais negativos multivariáveis de efeitos fixos em 5·647 municípios com qualidade adequada de estatísticas vitais. Também realizamos diversas análises de sensibilidade e triangulação. Integramos um conjunto de dados longitudinais com modelos validados de microssimulação dinâmica, e tendências projetadas nas taxas de mortalidade de menores de 5 anos em cenários alternativos de resposta política até 2030.

Resultados A elevada cobertura da atenção primária à saúde foi associada a reduções substanciais nas taxas de mortalidade de pós-neonatal (razão de taxa [RT] 0·72, IC 95% 0·71–0·74), nas taxas de mortalidade de crianças pequenas (ou seja, com idade entre 1 ano e <5 anos) taxas de mortalidade (0·75, 0·73–0·76) e taxas de mortalidade de menores de 5 anos (0·81, 0·80–0·82); prevenindo 305·890 (IC 95% 251·826–360·517) mortes de crianças com menos de 5 anos durante o período 2000–19. A elevada cobertura da atenção primária à saúde também foi associada a taxas mais baixas de mortalidade de menores de 5 anos por deficiências nutricionais (RT 0·55, IC 95% 0·52–0·58), anemia (0·64, 0·57–0·72), doenças imunopreveníveis sensíveis à vacina (0·70, 0·68–0·72), e gastroenterite infecciosa (0·78, 0·73–0·84). Considerando um cenário de crise econômica moderada, uma estratégia de resposta de mitigação implementada no período 2020–30 que aumente a cobertura da atenção primária à saúde poderia reduzir a taxa de mortalidade de menores de 5 anos em até 23% (RR 0·77, IC 95% 0·72–0·84) quando comparada com uma resposta de austeridade fiscal, e esta estratégia evitaria 142·285 (IC 95% 120·217–164·378) mortes de crianças até 2030 no Brasil, Colômbia, Equador e México.

Interpretação A melhoria na cobertura da atenção primária à saúde ao longo das últimas duas décadas no Brasil, Colômbia, Equador e México contribuiu substancialmente para melhorar a sobrevivência infantil. A expansão da cobertura da atenção primária à saúde deve ser considerada uma estratégia eficaz para mitigar os efeitos da atual crise económica na saúde e para alcançar os Objetivos de Desenvolvimento Sustentável relacionados com a saúde infantil.

Financiamento UK Medical Research Council.
