# Supplementary material for: Can primary health care mitigate the effects of economic crises on child health in Latin America? An integrated multicountry evaluation and forecasting analysis
Source: Lancet Glob Health. 2024 May 16;12(6):e938–46. doi: 10.1016/S2214-109X(24)00094-9 (PMC11126366; doi:10.1016/S2214-109X(24)00094-9)
Supplement: Supplementary appendix 3 [file mmc3.pdf]

THE LANCET
Global Health

Supplementary appendix 3

This appendix formed part of the original submission and has been peer reviewed. We post it as supplied by the authors.

Supplement to: Moncayo AL, Medeiros Cavalcanti D, Ordoñez JA, et al. Can primary health care mitigate the effects of economic crises on child health in Latin America? An integrated multicountry evaluation and forecasting analysis. *Lancet Glob Health* 2024; **12:** e938–46.

Supplementary Material

Can Primary Health Care mitigate the effects of economic crises on child health? An integrated multi-country evaluation and forecasting analysis in Latin America

TABLE OF CONTENTS

PART I – BACKGROUND ON PRIMARY HEALTH CARE IN THE COUNTRIES UNDER STUDY 2

1. **Introduction** 2
2. **Contextualization and further discussion of the findings** 4
3. **References** 7

PART II – SUMMARY OF PRIMARY HEALTH PROGRAMS, DATA SOURCES AND GENERAL METHODOLOGY 11

1. **Primary Healthcare Programs in BCEM countries** 11
   - 1.1. Primary Health Care Program- Brazil 11
   - 1.2. Primary Health Care Program- Colombia 11
   - 1.3. Primary Health Care Program – Ecuador 11
   - 1.4. Primary Health Care Program – México 12
   - 1.5. Mechanism of the effect of Primary Health Care (PHC) strategies on health outcomes 12
2. **Datasets** 13
   - 2.1. Data sources 13
   - 2.2. Interpolation and extrapolation method 15
   - 2.3. Some information about poverty rate 19
   - 2.4. Quality of Vital Statistics Method 20
     - 2.4.1. Discussion and Results – quality of CRVS method 20

PART III - RETROSPECTIVE ANALYSIS 23

3. **Empirical Methods** 23
   - 3.1. Negative binomial regression - Fixed Effects 23
4. **Results** 23
   - 4.1. Descriptive Analysis 23
   - 4.2. Triangulation – Difference-in-difference with propensity score matching 27
   - 4.3. Fit and sensitivity tests 29
5. **Deaths averted by PHC programs during 2000-19** 54

PART IV – FORECASTING ANALYSIS 55

6. **Description of the forecasting methodology** 55
7. **Purpose of the forecasting and its applications** 55
8. **Inputs, outputs, and other parameters** 55
   - 8.1. Scenarios of poverty and coverage of PHC programs 55
9. **Prediction methodology** 57
   - 9.1. External validation of each model 58
10. **Sensitivity analysis** 58
11. **Main Limitations** 59

REFERENCES 60

PART I – BACKGROUND ON PRIMARY HEALTH CARE IN THE COUNTRIES UNDER STUDY

1. Introduction

The Astana Declaration in 2018 emphasized the importance of health systems based on strong Primary Health Care (PHC), which is essential for achieving Universal Health Coverage (UHC) and should be an integral part of coordinated multisectoral actions addressing economic and social determinants of health.[1] According to this declaration, there is an urgent need to evaluate the effectiveness of PHC strategies in Low and Middle-Income Countries (LMIC) and how they can be developed, strengthened, and made more comprehensive, effective, and sustainable – especially for the achievement of Sustainable Development Goals (SDGs).[1] There is a growing consensus that health systems with stronger PHC have greater population coverage, respond better to local health needs, and provide comprehensive services more efficiently.[2] Several studies indicate that countries with well-developed PHC have better and more equitable health outcomes and provide stronger financial protection to citizens.[3]

The consequences of the COVID-19 pandemic, of the current wars, and of the global inflationary surge have dramatically increased socioeconomic vulnerabilities and health inequalities in LMICs,[4] with particularly detrimental effects on child health and child survival.[5,6] In addition, it is possible that the budget for health and social protection programs would be directly reduced due to measures of fiscal austerity,[7] which can further worsen the health of the population, in particular of the most vulnerable age groups such as children.[5,8] While during the COVID-19 pandemic several short-term emergency social protection interventions were implemented, the majority of them have already been dismantled in LMICs, while vulnerabilities and inequalities are high and, in some countries, still growing.[6]

Previous studies have documented the effects of social protection in the mitigation of the impact of economic crises on adult mortality,[7,9] but none has ever systematically evaluated the potential of interventions related to the access to healthcare, such as PHC, to attenuate the health impact of crises and create resilience, especially in the most vulnerable segments of the population, such as children. PHC mitigation effects in these circumstances are particularly plausible because PHC is particularly effective in improving the health of the poorest populations,[8,10] decreasing geographical, cultural, and economic barriers to healthcare and improving health equity.[11,12] Moreover, PHC is particularly effective in interventions of child health promotion and prevention, increasing prenatal visits, vaccination rates, child growth control, among many others.[11–13]

The Latin American and Caribbean (LAC) countries are among the ones which have suffered – and continue to suffer - the most from the socioeconomic consequences of the COVID-19 pandemic, the global inflationary surge, and widespread economic instabilities.[14,15] Moreover, they are among the LMIC that have increased more their public debt and could be forced to implement fiscal austerity measures,[16,17] reducing the budget for welfare state and healthcare services, inclusive PHC.

The LAC region is particularly interesting to evaluate PHC policies because several LAC countries started reforming their healthcare systems in the 1990s by developing frameworks to track improvements in care quality, enhancing primary healthcare (PHC), decentralizing health governance, bolstering regulatory measures, and increasing effectiveness.[13] In the LAC region, Brazil, Colombia, Ecuador, and Mexico (BCEM) - which represent 63% of LAC population, – are among the countries that have implemented strategies for nationwide delivery of public PHC. Brazil has one of the largest PHC programs in the world. The *Estratégia Saúde da Família* (ESF; Family Health Strategy) is the primary vehicle for achieving UHC within the Unified Health System and its coverage has expanded from 6·6% in 1998 to 62·6% in 2019.[14] The ESF encompasses key principles of PHC including community-based care, multi-disciplinary teams, and a focus on health prevention and promotion.[11] In 2004, Mexico established the health protection system (known as *Seguro Popular de Salud- SPS*), covering individuals in the informal sector or socioeconomically vulnerable. SPS has gradually expanded to include 51 million people by 2019 (about 41% of Mexico´s population).[16] Colombia´s subsidized scheme was introduced in 1993 (known as *Régimen Subsidiado -RS*) for low-income or informal-sector workers whose coverage increased from 15.7% in 1993 to 45·3% in 2019.[18] In 2008, Ecuador reformed the constitution to consider health care as a right and built a health care model (known as *Modelo de Atención Integral de Salud Familiar, Comunitario e Intercultural-* MAIS-FCI) based on Primary Health strategy with a focus on prevention and health promotion, and include community-based care and multi-disciplinary health teams. Under this model, the utilization of healthcare services increased by 300% during the period between 2008 and 2016 (for more details on each PHC strategy, see Appendix, p.2-4).[19] As a consequence, PHC has been implemented in different ways in Brazil, Colombia, Ecuador and Mexico (BCEM). In this study we have considered the ESF, SPS, RS, and MAIS-FCI as nationwide strategies to deliver public PHC inspired by the main principles of the Alma Ata and Astana Declaration,[20–22] promoting universal access to health services to everyone, fostering comprehensive care, including health promotion, health prevention, treatment, rehabilitation and palliation, with a health equity prospective and a particular focus on providing healthcare access to the most vulnerable populations.[23,24]

Though great progress has been made in PHC, both external and internal factors within the health sector have negatively impacted on the reach and effectiveness of PHC in LAC, such as inadequate government spending, health workforce shortages, and a lack of training in multisectoral actions, among others.[29,30] In this context, there are not studies that have ever evaluated the effectiveness of these PHC strategies over their entire implementation period, and that included multiple countries to estimate their impact as PHC strategy in the region, evaluating several causes of death and child age groups. In previous studies with short follow-up time, increasing coverage of Family Health Strategy was consistently associated with lower post-neonatal and child mortality in Brazil.[29] In Mexico, Seguro Popular was shown to reduce late neonatal mortality and infant mortality from conditions covered by the programme.[31] Moreover, no researches have ever systematically evaluated and forecasted the potential mitigation effect of cross-country PHC expansion strategies in periods of economic crises.

The objective of this study was to comprehensively evaluate the effects of PHC implementation strategies over the last two decades on child mortality – overall, for different causes, and for age subgroups - in Brazil, Colombia, Ecuador and Mexico.

Moreover, we aimed to forecast the mitigation effects of a potential PHC expansion, versus a reduction due to fiscal austerity, during the current economic crisis and beyond, up to 2030.

2. Contextualization and further discussion of the findings

The findings of our study show that PHC strategies had a significant impact on childhood mortality over the past two decades in four large countries of Latin America, having prevented more than 300,000 child death between 2000-2019 in Brazil, Colombia, Ecuador, and Mexico. Additional 140,000 child deaths could be potentially averted if PHC will be further expanded by 2030. PHC effects were particularly strong for post-neonatal and 1 to 4-years mortality, and mortality from anaemia, nutritional deficiencies, infectious gastroenteritis, and vaccine-preventable conditions. To our knowledge, this is the most comprehensive multi-country study on the effects of PHC on child health, and it is unique in its integration of a two-decades-long retrospective impact evaluation with dynamic microsimulation models to forecast PHC mitigation effects during an economic crisis.

Previous studies in Brazil and Mexico over shorter periods have shown that PHC strategies could reduce child and infant mortality,[29,31] in our study, we found a stronger and dose-response effect on post-neonatal mortality and mortality between 1 to 4 years. This is expected because PHC has been shown to increase access to routine immunization in this age period, preventing infectious diseases that contribute to child mortality.[20,44] As a matter of fact, one of the strongest PHC effects we found was on mortality from vaccine-preventable diseases and sensitive conditions, and our complementary analyses (Appendix,p.35, Table 24) showed a dose-response effect of PHC in the increase of vaccination rates. Moreover, PHC has also been associated to better postnatal care, in terms of support for breastfeeding, child growth control, health and nutrition education of the mothers.[21,45] In fact, PHC promotes important strategies that have been recognized as the most effective interventions to reduce childhood mortality due to nutritional conditions worldwide, such as: oral rehydration therapy, exclusive breastfeeding, correct weaning practices, micronutrients supplementation, personal hygiene, and food preparation practices.[46] In addition, primary health services are well-placed to assess patient´s diets, screen for dietary risk factors, to diagnose diet-related disease, and to take appropriate action.[47] As a matter of fact, our findings show a stronger effect of PHC on under-five mortality from Nutritional Deficiencies and Anemia. PHC also allows a timelier diagnosis and treatment of common childhood illnesses such as pneumonia, diarrhea, and malaria, reducing geographical, economical, and cultural barriers to child healthcare, especially for the most vulnerable populations, and ultimately contributing to reduced child mortality.[12,23] Indeed, we found a strong impact of PHC on mortality from infectious gastroenteritis and bacterial pneumonias. Similar results were showed in two studies in Brazil with a strong effect of PHC on mortality for diarrhoea and lower respiratory infections.[33] PHC can also reduce child mortality promoting health education, empowering families to make informed decisions about their children's health and well-being, often through the work and domiciliary visits of community health workers, physicians, and nurses, besides educational activities in the health posts.[30,36,48] Moreover, PHC promote community engagement and empowerment, involving communities in health decision-making, and promoting community-based health services.[37,49]

Additionally, our findings indicate that primary health care (PHC) can decrease neonatal mortality. While neonatal deaths are more linked to birth complications or congenital conditions than to primary care assistance, PHC facilitates early and regular antenatal check-up,[50] ensuring timely identification and management of potential complications, besides executing family planning services.[38,51] In fact, our complementary analyses (Appendix,p.35, Table 24) showed a dose-response effect of PHC coverage in the increase of prenatal care activities.

While previous studies[32,52] have already evaluated the impact of nationwide Primary Health Care on infant mortality within specific countries, our study represents the first comprehensive attempt to assess the effects of PHC as a macro-strategy across four countries, collectively representing the majority of the Latin American population. Despite inherent differences in characteristics and implementation of PHC, both between and within these countries, our findings highlight the strong effects of PHC as strategy to improve health and reduce infant and child mortality in Low- and Middle-Income Countries (LMIC). These results have a particular importance in the context of the discussions about PHC, given the common criticism on its heterogeneity and incomplete implementation across the majority of LMIC.[53,54] While it is acknowledged that PHC struggle with challenges such as fragmentation, inadequate integration with other healthcare services, insufficient funding and resources, inadequate workforce and training, limited community engagement, among others,[55–57] it is equally crucial to recognize that, despite these limitations and variations in its implementation, PHC has played a pivotal role in improving the overall health of populations, especially among the most vulnerable age groups, over the past two decades.

The sustainability of health systems investments to achieve and maintain PHC and UHC in LMICs has been affected by the global economic crisis, which started with the COVID-19 pandemic and is continuing with the global consequences of the war in Ukraine, among other factors.[4] As consequence, Latin America countries faced a reduction in economic growth, inflationary pressures, and declining value of their currencies. Also, these countries experienced high unemployment and poverty, increased inequality, and social exclusion.[9,58] The most common political response to these economic downturns in LAC has historically been the implementation of fiscal austerity measures to reduce the public debt, and this is often translated into the reduction of social protection and healthcare services. For instance, Brazil's SUS continues to be underfunded as a result of the federal health budget's stagnation with only a 3.2% growth in public health spending over the previous 10 years.[13] Colombia has also plagued by underfunding of its subsidized POS (*Plan Obligatorio de Salud*) component and Mexico faced a 25% deficit in health spending.[13] Ecuador also had a stagnation in health spending in last years and even a decrease during the pandemic.[59] Our forecast analyses broadens the conclusions of other studies,[8,27] showing how in LAC the implementation of fiscal austerity measures affecting PHC will have a significant negative impact on child health outcomes, potentially causing a large number of preventable child deaths in the next decade. In the upcoming years, additional studies will be necessary to assess which specific characteristics of Primary Health Care (PHC) contribute to its ability to mitigate and demonstrate resilience in the face of socioeconomic crises.

Our study also shows, as many other evaluation studies conducted in the last years in Latin America,[21,32,34,36] that the presence of sufficient and high-quality administrative data, encompassing demographic, socioeconomic, and health-related information, enables the development of highly relevant studies that can guide evidence-based decision-making processes.

The study's limitations include the exclusive use of municipalities with adequate quality of vital statistics, to improve the internal validity of our analyses. These municipalities represent more than 85% of the total population in BCEM, because the municipalities with lower/inadequate quality of vital information are also the smallest municipalities in terms of population. Our models - fitted with data from all BCEM municipalities - returned similar dose-response and statistically significant effects. Another limitation of our study is its ecological design, because there is the possibility of ecological fallacy, wherein associations observed at an ecological level may not accurately represent associations at the individual level. Even if our focus is on the ecological inference, assessing the effectiveness of a health policy at an aggregate scale, the plausibility of our ecological associations depends, to some extent, on associations at the individual level. Specifically, in our study, the inability to definitively establish whether individuals experiencing the outcome (such as deaths in children less than 5-years old) were covered by PHC arises, because our available information is only at the municipal level. Nevertheless, it is crucial to note that PHC implementation prioritizes deprived neighborhoods within municipalities, where the incidence of child preventable mortality is concentrated due to unfavorable socioeconomic conditions and various barriers to healthcare. In such instances, the reduction in these preventable deaths (as shown in Table3, mainly due to infectious gastroenteritis, anemia, nutritional deficiencies, and bacterial pneumonias, among others), after accounting for all confounding factors and selection biases, can reasonably be attributed to the inclusion of these vulnerable individuals in PHC catchment areas. Additionally, the use of small units of analysis, such as in our case municipalities, allow to diminish the risk of ecological fallacy.[60] It has also to be considered that ecological-level designs have been consistently used in several nationwide evaluations to estimate the impact of public policies in Latin America.[9,20,36] Another limitation of this study is that we used a simple indicator of populational PHC coverage rather than a more refined indicator of effective PHC coverage.[28] Consequently, while increased populational PHC coverage suggests a greater capacity to provide essential health services to the population, it does not necessarily reflect the quantity and quality of the service provided by PHC teams.

An additional constraint of the study is the heterogeneity of the strategies used to deliver public PHC evaluated in the four countries, and the diversity of their operational indicators of coverage. In the BCEM, there were two very similar strategies of public PHC: the ESF (Brazil) and MAIS-FCI (Ecuador). They represent together the majority of the municipalities and of the population in the BCEM group, and they could be considered the more conventional strategy to deliver PHC, using PHC teams with well-established catchment areas, for which they are responsible and where they deliver PHC actions.[21,32–34,52] On the other side, *Seguro Popular de Salud* - SPS (Mexico) and *Régimen Subsidiado* - RS (Colombia), are essentially subsidized medical insurance plans that focus on providing basic healthcare services for individuals with low incomes, mostly those who are unemployed and working in the informal economy. Both are solidly grounded and focused in free-of-charge, low-complexity primary health care, while they also provide – to a

lesser extent - secondary and tertiary levels of care.[61,62] Both SP and SR can be considered more as strategies to deliver PHC than well-defined PHC programs itself. However, we have used the population coverage of these programs as a proxy for PHC coverage for multiple reasons, including that they offer healthcare services mainly focused on PHC, individuals affiliated to them are all covered by their PHC services, they have an equity approach, and they are focused on the most vulnerable populations. Moreover, child health in SP and RS is primarily addressed at their PHC level, and our models included as adjusting variables hospital bed rates and physicians rates, able to adjust for the effects of higher-than-PHC levels of care. Furthermore, our fixed effects specifications can adjust for the effects of unobserved time-invariant variables, such as hospital structures in the municipalities. As a matter of fact, in the sensitivity analyses, we controlled for the different types of PHC strategies in these countries, and the effect estimates were consistently similar to our main results (Appendix,p.31,Table 19). Therefore, despite the heterogeneity in the strategies to deliver public PHC and in the PHC populational coverage indicators in the four countries under study, our effect estimates could be considered robust, consistent, and representative of the impact of the cross-country implementation of the PHC strategies in the last two decades.

In conclusion, our results demonstrate the fundamental role of Primary Health Care strategies in reducing childhood mortality in four large countries of Latin America - and potentially in all LMIC - over the last two decades. Moreover, all our forecast scenarios show that a prompt expansion of PHC coverage, to protect the growing numbers of vulnerable populations, could represent an effective policy to mitigate the adverse health impact of the current economic crises. On the contrary, reductions in PHC coverage due to austerity measures could be responsible for a significant number of preventable child deaths in coming years and may prevent LMIC from achieving SDGs related to child health and child mortality.

3. References

1 Kluge H, Kelley E, Swaminathan S, *et al.* After Astana: building the economic case for increased investment in primary health care. *The Lancet* 2018; **392**: 2147–52.

2 Macinko J, Starfield B, Erinosho T. The Impact of Primary Healthcare on Population Health in Low- and Middle-Income Countries. *Journal of Ambulatory Care Management* 2009; **32**: 150–71.

3 Starfield B. Primary care: an increasingly important contributor to effectiveness, equity, and efficiency of health services. SESPAS report 2012. *Gac Sanit* 2012; **26**: 20–6.

4 United Nations. World Economic Situation and Prospects 2023. New York, 2023 https://desapublications.un.org/publications/world-economic-situation-and-prospects-2023 (accessed Dec 7, 2023).

5 Castro A. Challenges posed by the COVID-19 pandemic in the health of women, children, and adolescents in Latin America and the Caribbean. New York, 2020.

6 Molina GG, Montoya-Aguirre M, Ortiz-Juarez E. Addressing the cost-of-living crisis in developing countries: Poverty and vulnerability projections and policy responses. New York, 2022.

7 Stubbs T, Kentikelenis A, Gabor D, Ghosh J, McKee M. The return of austerity imperils global health. *BMJ Glob Health* 2023; **8**: e011620.

8 Rasella D, Basu S, Hone T, Paes-Sousa R, Ocké-Reis CO, Millett C. Child morbidity and mortality associated with alternative policy responses to the economic crisis in Brazil: A nationwide microsimulation study. *PLoS Med* 2018; **15**: e1002570.

9 Hone T, Mirelman AJ, Rasella D, *et al.* Effect of economic recession and impact of health and social protection expenditures on adult mortality: a longitudinal analysis of 5565 Brazilian municipalities. *Lancet Glob Health* 2019; **7**: e1575–83.
10 Langlois E V, McKenzie A, Schneider H, Mecaskey JW. Measures to strengthen primary health-care systems in low- and middle-income countries. *Bull World Health Organ* 2020; **98**: 781–91.
11 Hone T, Rasella D, Barreto M, Atun R, Majeed A, Millett C. Large Reductions In Amenable Mortality Associated With Brazil's Primary Care Expansion And Strong Health Governance. *Health Aff* 2017; **36**: 149–58.
12 Bhutta ZA, Ali S, Cousens S, *et al.* Interventions to address maternal, newborn, and child survival: what difference can integrated primary health care strategies make? *The Lancet* 2008; **372**: 972–89.
13 Gilardino RE, Valanzasca P, Rifkin SB. Has Latin America achieved universal health coverage yet? Lessons from four countries. *Archives of Public Health* 2022; **80**: 38.
14 Giovanella L, Bousquat A, Schenkman S, Almeida PF de, Sardinha LMV, Vieira MLFP. Cobertura da Estratégia Saúde da Família no Brasil: o que nos mostram as Pesquisas Nacionais de Saúde 2013 e 2019. *Cien Saude Colet* 2021; **26**: 2543–56.
15 World Bank. From Infection to Inflation. LAC Poverty and Labor Brief. Washington, DC, 2023 http://hdl.handle.net/10986/39806 (accessed Dec 7, 2023).
16 CONEVAL. Sistema de Protección Social en Salud: Seguro Popular y Seguro Médico Siglo XXI. Mexico, 2019.
17 Cohen MA. Austerity and the Global Crisis: Lessons from Latin America. In: Ghilarducci T, McGahey R, Mack A, eds. AUSTERITY: Failed Economics but Persistent Policy (FALL 2013), 3rd edn. Baltimore: Johns Hopkins University Press, 2013: 929–52.
18 Gobierno de Colombia. Informe Global de Colombia Sobre Indicadores de Progreso para la Medición de Derechos Contemplados en el Protocolo de San Salvador. Colombia, 2020.
19 Flores Jimenez SE, San Sebastián M. Assessing the impact of the 2008 health reform in Ecuador on the performance of primary health care services: an interrupted time series analysis. *Int J Equity Health* 2021; **20**: 169.
20 Moncayo AL, Granizo G, Grijalva MJ, Rasella D. Strong effect of Ecuador's conditional cash transfer program on childhood mortality from poverty-related diseases: A nationwide analysis. *BMC Public Health* 2019; **19**. DOI:10.1186/s12889-019-7457-y.
21 Rasella D, Aquino R, Barreto ML. Impact of the Family Health Program on the quality of vital information and reduction of child unattended deaths in Brazil: an ecological longitudinal study. 2010 http://www.biomedcentral.com/1471-2458/10/380.
22 B. I. Ткаченко. Primary Health Care in the World: Results of the 40-year Implementation of the Almaty Declaration (1978) and Future Development According to the Astana Declaration (2018). *Fam Med* 2019; : 97–102.
23 Schilling Mendonça C, Bielefeldt Leotti V, Soares Dias-da-Costa J, Harzheim E. Hospitalizations for primary care sensitive conditions: association with socioeconomic status and quality of family health teams in Belo Horizonte, Brazil. *Health Policy Plan* 2017; **32**: 1368–74.
24 Kluge H, Kelley E, Swaminathan S, *et al.* After Astana: building the economic case for increased investment in primary health care. *The Lancet* 2018; **392**: 2147–52.
25 Mariano T da SO, Nedel FB. Hospitalização por Condições Sensíveis à Atenção Primária em menores de cinco anos de idade em Santa Catarina, 2012: estudo descritivo*. *Epidemiologia e Serviços de Saúde* 2018; **27**. DOI:10.5123/S1679-49742018000300006.
26 BRASIL. Publica a lista brasileira de internações por condições sensíveis à atenção primária. Brazil: Ministério da Saúde, 2008.
27 Jannati A, Sadeghi V, Imani A, Saadati M. Effective coverage as a new approach to health system performance assessment: a scoping review. *BMC Health Serv Res* 2018; **18**: 886.
28 Leslie HH, Malata A, Ndiaye Y, Kruk ME. Effective coverage of primary care services in eight high-mortality countries. *BMJ Glob Health* 2017; **2**: e000424.c

29 Bastos ML, Menzies D, Hone T, Dehghani K, Trajman A. The impact of the Brazilian family health on selected primary care sensitive conditions: A systematic review. *PLoS One* 2017; **12**: e0182336.

30 Chotchoungchatchai S, Marshall AI, Witthayapipopsakul W, Panichkriangkrai W, Patcharanarumol W, Tangcharoensathien V. Primary health care and sustainable development goals. *Bull World Health Organ* 2020; **98**: 792–800.

31 Celhay P, Martinez S, Muñoz M, Perez M, Perez-Cuevas R. Long-term effects of public health insurance on the health of children in Mexico: a retrospective study. *Lancet Glob Health* 2019; **7**: e1448–57.

32 Aquino R, de Oliveira NF, Barreto ML. Impact of the Family Health Program on Infant Mortality in Brazilian Municipalities. *Am J Public Health* 2009; **99**: 87–93.

33 Rasella D, Aquino R, Barreto ML. Reducing childhood mortality from diarrhea and lower respiratory tract infections in brazil. *Pediatrics* 2010; **126**. DOI:10.1542/peds.2009-3197.

34 Rasella D, Harhay MO, Pamponet ML, Aquino R, Barreto ML. Impact of primary health care on mortality from heart and cerebrovascular diseases in Brazil: a nationwide analysis of longitudinal data. *BMJ* 2014; **349**: g4014–g4014.

35 Giovanella L, Almeida PF de, Vega Romero R, Oliveira S, Tejerina Silva H. Panorama de la Atención Primaria de Salud en Suramérica: concepciones, componentes y desafíos. *Saúde em Debate* 2015; **39**: 300–22.

36 Rasella D, Aquino R, Santos CAT, Paes-Sousa R, Barreto ML. Effect of a conditional cash transfer programme on childhood mortality: A nationwide analysis of Brazilian municipalities. *The Lancet* 2013; **382**: 57–64.

37 Frenk J, Gómez-Dantés O, Knaul FM. The democratization of health in Mexico: financial innovations for universal coverage. *Bull World Health Organ* 2009; **87**: 542–8.

38 Dufrénot G, Mignon V, Péguin-Feissolle A. The effects of the subprime crisis on the Latin American financial markets: An empirical assessment. *Econ Model* 2011; **28**: 2342–57.

39 Economic Commission for Latin America and the Caribbean (ECLAC). Latin America and the Caribbean in the World Economy. Santiago, Chile, 2014 https://repositorio.cepal.org/server/api/core/bitstreams/6d6ac875-3ab0-4de7-b534-4c07f466ef15/content (accessed Dec 7, 2023).

40 Heckman JJ. Sample Selection Bias as a Specification Error. *Econometrica* 1979; **47**: 153.

41 Lawlor DA, Tilling K, Davey Smith G. Triangulation in aetiological epidemiology. *Int J Epidemiol* 2017; : dyw314.

42 Rasella D, Hone T, de Souza LE, Tasca R, Basu S, Millett C. Mortality associated with alternative primary healthcare policies: a nationwide microsimulation modelling study in Brazil. *BMC Med* 2019; **17**: 82.

43 Caro JJ, Briggs AH, Siebert U, Kuntz KM. Modeling Good Research Practices—Overview: A Report of the ISPOR-SMDM Modeling Good Research Practices Task Force-1. *Value in Health* 2012; **15**: 796–803.

44 Rainey JJ, Watkins M, Ryman TK, Sandhu P, Bo A, Banerjee K. Reasons related to non-vaccination and under-vaccination of children in low and middle income countries: Findings from a systematic review of the published literature, 1999–2009. *Vaccine* 2011; **29**: 8215–21.

45 Bhutta ZA, Das JK, Rizvi A, *et al.* Evidence-based interventions for improvement of maternal and child nutrition: what can be done and at what cost? *The Lancet* 2013; **382**: 452–77.

46 Jones G, Steketee RW, Black RE, Bhutta ZA, Morris SS. How many child deaths can we prevent this year? *The Lancet* 2003; **362**: 65–71.

47 Kraef C, Wood B, von Philipsborn P, Singh S, Peterson SS, Kallestrup P. Primary health care and nutrition. *Bull World Health Organ* 2020; **98**: 886–93.

48 Perry HB, Zulliger R, Rogers MM. Community Health Workers in Low-, Middle-, and High-Income Countries: An Overview of Their History, Recent Evolution, and Current Effectiveness. *Annu Rev Public Health* 2014; **35**: 399–421.

49 Kluge H, Kelley E, Birtanov Y, *et al.* Implementing the renewed vision for Primary Health Care in the Declaration of Astana: the time is now. *Prim Health Care Res Dev* 2019; **20**: e158.

50 Al-Nasser AN, Bamgboye EA, Abdullah FA. Providing antenatal services in a primary health care system. *J Community Health* 1994; **19**: 115–23.

51 Dumitru D, Sarah S, Corina C, *et al.* Family Planning Programs – An Opportunity to Integrate Preconception Health Services into Primary Health Care. *International Journal of Biomedicine* 2021; **11**: 467–72.

52 Macinko J. Evaluation of the impact of the Family Health Program on infant mortality in Brazil, 1990-2002. *J Epidemiol Community Health (1978)* 2006; **60**: 13–9.

53 Jungo KT, Anker D, Wildisen L. Astana declaration: a new pathway for primary health care. *Int J Public Health* 2020; **65**: 511–2.

54 Walraven G. The 2018 Astana Declaration on Primary Health Care, is it useful? *J Glob Health* 2019; **9**. DOI:10.7189/jogh.09.010313.

55 Maeseneer J De, Willems S, Sutter A De, Geuchte V de, Billings M. Primary health care as a strategy for achieving equitable care: a literature review commissioned by the Health Systems Knowledge Network. Johannesburg: Centre for Health Policy, 2007 https://biblio.ugent.be/publication/396406/file/1041490 (accessed Dec 7, 2023).

56 Dolea C, Stormont L, Braichet J-M. Evaluated strategies to increase attraction and retention of health workers in remote and rural areas. *Bull World Health Organ* 2010; **88**: 379–85.

57 Rohde J, Cousens S, Chopra M, *et al.* 30 years after Alma-Ata: has primary health care worked in countries? *The Lancet* 2008; **372**: 950–61.

58 United Nations. The Sustainable Development Goals Report 2021. New York, 2021 https://desapublications.un.org/publications/category/Sustainable%20Development/year/2021 (accessed Dec 7, 2023).

59 OIT. El sistema de salud ecuatoriano y la COVID-19. Lima, Peru, 2021.

60 Wakefield J. Ecologic Studies Revisited. *Annu Rev Public Health* 2008; **29**: 75–90.

61 Leyva-Flores R, Servan-Mori E, Infante-Xibille C, Pelcastre-Villafuerte BE, Gonzalez T. Primary Health Care Utilization by the Mexican Indigenous Population: The Role of the Seguro Popular in Socially Inequitable Contexts. *PLoS One* 2014; **9**: e102781.

62 Montenegro Torres F, Bernal Acevedo O. Colombia Case Study: The Subsidized Regime of Colombia’s National Health Insurance System. Washington DC, 2013 http://hdl.handle.net/20.500.12424/530793 (accessed Dec 7, 2023).

PART II – SUMMARY OF PRIMARY HEALTH PROGRAMS, DATA SOURCES AND GENERAL METHODOLOGY

1. Primary Healthcare Programs in BCEM countries

1.1. Primary Health Care Program- Brazil

The "Estratégia Saúde da Família" (ESF; Family Health Strategy – previously called Family Health Program), is the primary vehicle for achieving Universal Health Coverage (UHC) within the SUS and is one of the largest Primary Health Care (PHC) programs of the world. Implemented since 1994, the FHS encompasses key principles of PHC including community-based care, multi-disciplinary teams, and a focus on health prevention and promotion, composed of physicians, nurses, and community health workers to provide basic health care and preventive services to families and households in selected communities.[1] The FHS teams reinforces the promotion, prevention, protection, diagnosis, treatment, rehabilitation, harm reduction, palliative care and health surveillance of the Unified Health System (SUS) in Brazil.[2]

The FHS coverage has expanded from 6·6% in 1998 to 63·7% in 2016 (covering 123 million people).[3] Prior studies associated expanding FHS coverage with reductions in infant mortality,[4] adult mortality from conditions amenable to healthcare,[1] cardiovascular mortality[5] and health inequalities.[6] Despite the large amount of evidence of its effectiveness, according to recent forecasting studies performed by the proponents of the project, austerity measures implemented in the country could affect future ESF coverage, especially in the poorest areas, and be responsible for a large number of avoidable child and adult deaths in the next years.[3]

1.2. Primary Health Care Program- Colombia

Within Colombia's General Social Security System in Health, which was created by the so-called Law 100 of 1993, the Subsidized Regime ("Régimen Subsidiado") is the non-contributory healthcare insurance plan for individuals with low incomes, mostly those working in the informal economy.[7] It is funded by taxes as well as contributions from the Contributory Regime (Régimen Contributivo), the healthcare plan for individuals with sufficient ability to pay that mostly work in the formal economy, and covers most low-complexity care, but provides only limited coverage for most hospital care.[8] To fill the gap, the Subsidized Regime is complemented by services provided by public hospitals, financed through direct payments to providers independent of what services they supply and of patients' insurance status. The Subsidized Regime covers about 55% of the total population.

1.3. Primary Health Care Program – Ecuador

In 2008, Ecuador underwent a major health reform with the aim of universal coverage.[9] The constitution states the right to comprehensive health care based on principles of equity, quality, and efficiency. This policy, along with the Social Development Agenda and the Millennium Development Goals, was key to build the Model of Comprehensive Family, Community and Intercultural Health Care (MAIS-FCI for its Spanish acronym). This model is based on Primary Health Care (PHC) strategy to strengthen the decision-making capacity in the first level of attention, with a focus on prevention and health promotion and include community-based care and multi-disciplinary teams.[10] New

hospitals and primary level health centers were punt into operation, allowing for an increase of over 300% in demand for health services with a total investment of US.16,208 million between 2007 and 2016 and an average annual investment five times higher than the period 2000-2006.[11]

1.4. Primary Health Care Program – México

In 2003, the Mexican government initiated a comprehensive reform of its public health sub-system to enhance the financial protection of approximately 50 million individuals who did not have coverage under existing mandatory contributory insurance schemes that catered to formal workers. As part of this reform, the *Seguro Popular de Salud (SPS),* a voluntary public health insurance program, was developed and implemented.[12] SPS specifically targeted the most economically disadvantaged segments of the population who were without health insurance, with the aim of expanding healthcare services and addressing disparities in health financing. Since its introduction in 2004, SPS has progressively grown to include 55.6 million people.[12]

1.5. Mechanism of the effect of Primary Health Care (PHC) strategies on health outcomes

Population's overall health is a complex construct with multiple determinants. While fundamental causes, such as the underlying social determinants of health and inequality largely shape risk factors in the general population--with particularly strong impacts on those at the lower end of the socioeconomic spectrum--numerous factors may intervene to disrupt these negative health impacts. PHC has been conceptualized[13] as one of the main hubs of the broader network of interventions that attempt to prevent illness and disability and promote healthy lifestyles and behaviors. PHC serves as the main entry point to the health system for curative and rehabilitative care. Numerous studies have documented the importance of effective PHC on health system effectiveness, equity, and overall health outcomes.[1, 14, 15] While the relationship between PHC and other levels of the health system has been well documented, an increasingly important role has been identified for PHC as a means of synergistically connecting the healthcare system with other aspects of the welfare state, such as social assistance and poverty relief interventions including conditional cash transfer programs (CCTs), Social Pensions (SP), and other programs, such as those related to housing and caring for people with disabilities, among others.[16-18] For example, the social determinants of health, and poverty in particular, strongly affect both health outcomes of individuals and the ability of PHC to improve them. Such interrelationships likely require different strategies at the PHC level.[19, 20] On the other hand, poverty is also one barrier for access to healthcare, and poverty-relief interventions should be able to improve PHC access, although the current evidence on this relationship is limited at best.[21] The model also shows that PHC has the potential to deliver directly (or facilitate referrals) for preventive interventions to children at risk of poverty-related disparities, in particular for early child development.[22] PHC can be considered an efficient setting to deliver advice on welfare rights and on social interventions eligibility especially for the less educated and poorer individuals, but there is a scarcity of studies on these aspects.[23, 24] A recent study in the UK showed no effect of this strategy on the health of more affluent elderly, but the impact on vulnerable populations could well be different. The Framework below shows this mechanism of the effect of PHC strategies on health outcomes.

FIGURE 1. Conceptual Framework of the mechanism of the effect of Primary Health Care Strategies on child health outcomes

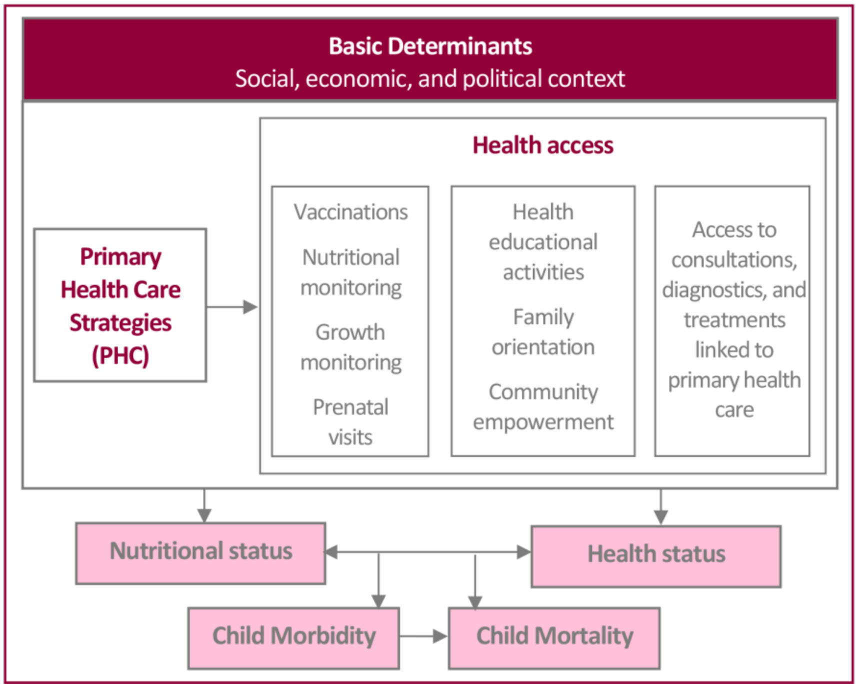

2. Datasets

2.1. Data sources

The data used in this study was obtained from various governmental platforms, detailed in Table 1. All the variables used in this study are aggregated in a municipal level. However, there was not data available for specific years and municipalities. Therefore, it was performed an exponential decay method for interpolation, as detailed in Section 2.2. of this supplementary document.

TABLE 1. Sources and description for Brazil and Ecuador

Variable	BRAZIL				ECUADOR			
	**Years**	**Units of analysis**	**Source**	**Link**	**Years**	**Units of analysis**	**Source**	**Link**
Mortality and morbidity	2000 to 2019	Individual	DATASUS -SIM	Link 1	2000 to 2019	Individual	INEC	Link 8
Population estimation	2000 to 2019	Municipal	IBGE-Census	Link 2	2000 to 2019	Municipal	INEC	Link 9
Livebirth	2000 to 2019	Municipal	DATASUS -SINASC	Link 3	2000 to 2019	Municipal	INEC	Link 10
Socioeconomic variables	2000 and 2010, 2001 - 2019	State and municipal	Census, PNAD, and PNADC (IBGE)	Link 4	2001 and 2010	Municipal	INEC	Link 11

Variable	BRAZIL				ECUADOR			
	**Years**	**Units of analysis**	**Source**	**Link**	**Years**	**Units of analysis**	**Sourc e**	**Link**
PHC coverage	2000 to 2019	Municipal	DATASUS -DAB	Link 5	2000 to 2019	Municipal	INEC	Link 12
Hospital bed rate (beds per 1.000 inhabitants)	2000 to 2019	Municipal	DATASUS -CNES	Link 6	2004 to 2019	Municipal	INEC	Link 13
Doctors rate (Physicians per 1.000 inhabitants)	2000 to 2019	Municipal	DATASUS -CNES	Link 7	2000 to 2017	Municipal	INEC	Link 14

Note: **BRAZIL SOURCES**: DATASUS – Department of informatics of the Unified Health System (Departamento de Informátiva do Sistema Único de Saúde); SIM – Mortality Information Systrem (Sistema de Informações sobre Mortalidade); DAB – Department of Primary Care (Departamento de Atenção Básica); CNES – National Register of Health Establishments (Cadastro Nacional de Estabelecimentos de Saúde); SINACS – Live Birth Information System (Sistema de Informações sobre Nascidos Vivos); IBGE – Brazilian Institute of Geography and Statistics (Instituto Brasileiro de Geografia e Estatística); MDS – Ministry of Social Development and Fight against Hunger (Ministério do Desenvolvimento Social e Combate à Fome); PNAD – (Pesquisa Nacional por Amostra de Domicílios); PNADC – Continuous PNAD Quaterly (Pesquisa Nacional por Amostra de Domicílios Contínua). | **ECUADOR SOURCES**: INEC: National Institute of Statistics and Census (Instituto Nacional de Estadísticas y Censos).

TABLE 2. Sources and description for México and Colombia

Variable	MEXICO				COLOMBIA			
	**Years**	**Units of analysis**	**Source**	**Link**	**Years**	**Units of analysis**	**Source**	**Link**
Mortality and morbidity	2000 to 2020	Individual	INEGI	Link 15	2000 to 2019	Individual	DANE	Link 22
Population estimation	2000 to 2020	Municipal	CONAPO	Link 16	2000 to 2019	Municipal	DANE	Link 23
Livebirth	2000 to 2019	Municipal	DGIS	Link 17	2000 to 2019	Municipal	DANE	Link 24
Socioeconomic variables	2000, 2005, 2010 and 2020	Municipal	CONEVAL	Link 18	2002 to 2019	Municipal	DANE	Link 25
PHC coverage	2004 to 2019	Municipal	CONAPO	Link 19	2000 to 2015	Municipal	CEDE	Link 26
Hospital bed rate (beds per 1.000 inhabitants)	2001 to 2020	Municipal	DGIS	Link 20	2010 to 2019	Municipal	REPS	Link 27
Doctors rate (Physicians per 1.000 inhabitants)	2001 to 2020	Municipal	DGIS	Link 21	2010 to 2019	Municipal	ReThus	Link 28

Note: **MEXICO SOURCES**: INEGI – National Institute of Statistics and Geography (Instituto Nacional de Estadística y Geografía); CONAPO – National Population Council (Consejo Nacional de Población); CONEVAL – National Council of Evaluation of the Social Development Policy (Consejo Nacional de Evaluación de la Política de Desarollo Social); DGIS – General Directorate of Health Information (Dirección General de Información en Salud); DGAM – Open Data - Government of Mexico (Datos Abiertos – Gobierno de Mexico). **COLOMBIA SOURCES:** DANE - National Administrative Department of Statistics **(**Departamento Administrativo Nacional de Estadística); CEDE - Center for Economic Development Studies (Centro de Estudios sobre Desarrollo Económico); REPS – Special Registry of Healthcare Providers (Registro Especial de Prestadores de Servicios de Salud); ReTHUS – National Unique Registry of Human Talent (Registro Único Nacional de Talento Humano).

2.2. Interpolation and extrapolation method

Some of the control variables are not available in the period of 2000-2019. In these cases, it was used the exponential decay method to extrapolate the available variables at least in two points of the timeline. There were dropped off the municipalities with only one element.

The interpolated variables were illiteracy rate, household infrastructure (sewage and piped water), hospital bed rate, doctor rate, inequality, and income variables (Gini index and poverty rate). Some of these variables were used as control variables in the models; none of the outcome variables (mortality) or exposure variables (PHC coverage) were interpolated. At the end, it was observed that the interpolated and extrapolated variables improve the control and precision of the retrospective and predictive models.

FIGURE 2. Gini index boxplot for selected municipalities in Brazil, Colombia, Ecuador, and Mexico (BCEM), from the period 2000-19.

EACH COUNTRY – GINI INDEX | ALL COUNTRIES (BCEM) – GINI INDEX

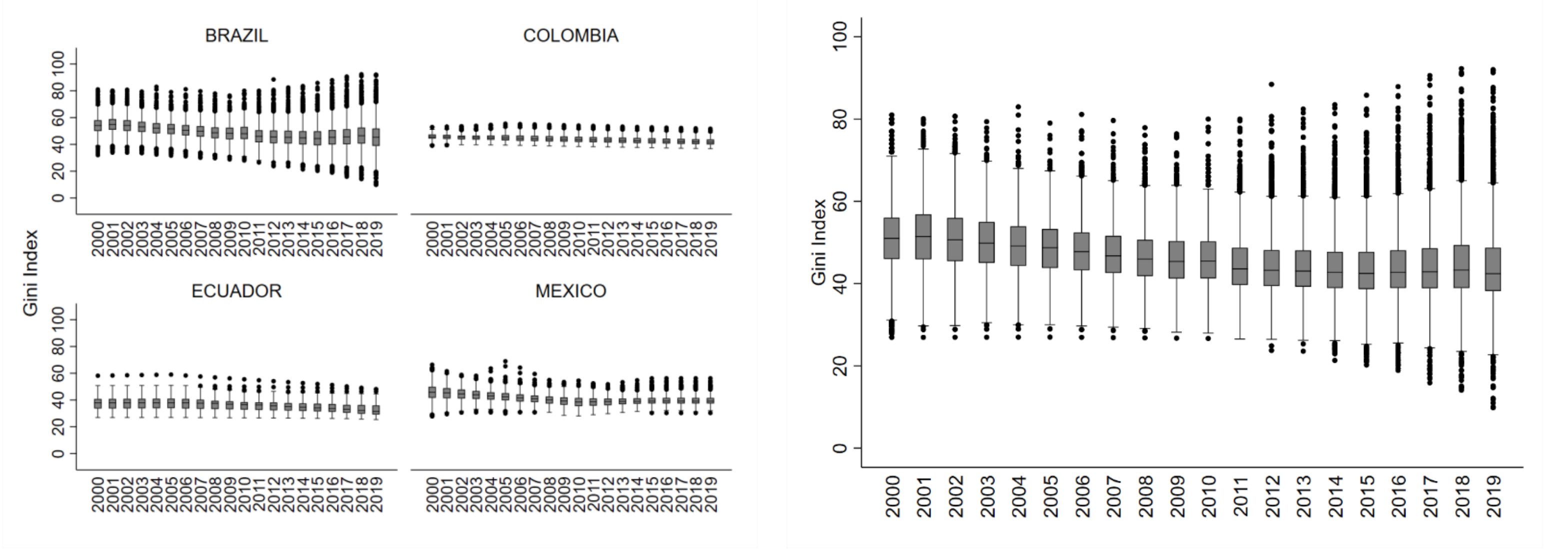

Source: Author's plot for 8,332 selected municipalities in Brazil, Colombia, Ecuador, and Mexico, over 20 years (from 2000 to 2019).
Note: We selected municipalities with adequate quality of civil registration and vital statistics (CRVS). In Brazil, the Gini index was available at the municipal level for the years 2000 and 2010, and the period 2001-09 and 2011-119 were extrapolated. In Colombia, Gini index was available for the period 2002-2019, and we extrapolate the years 2000 and 2001. Ecuador has the Gini index available at the municipal level for the years 2005 and 2014, and we extrapolate the period 2000-04, and 2015-19. In Mexico, the Gini index at the municipal level is available for the years 2000, 2002, and 2004-19, and we only extrapolate the years 2001 and 2003.

FIGURE 3. Illiteracy rate boxplot for selected municipalities in Brazil, Colombia, Ecuador, and Mexico, (BCEM), from the period 2000-19.

EACH COUNTRY – ILLITERACY RATE

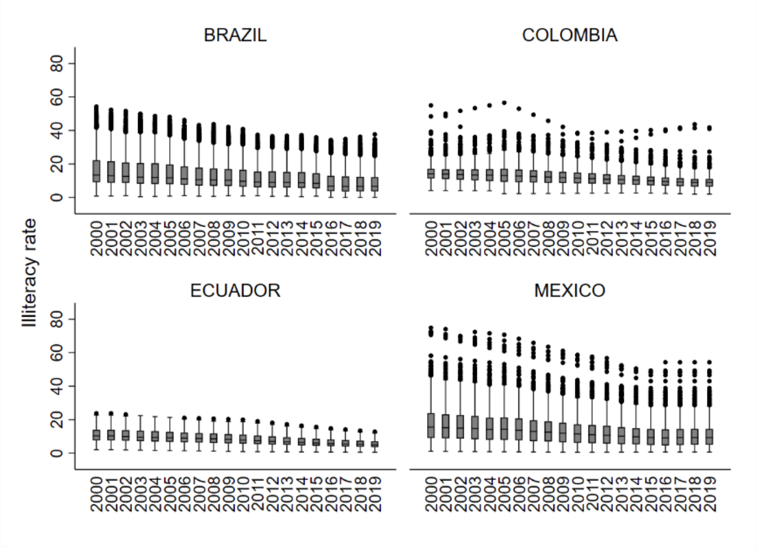

ALL COUNTRIES (BCEM) – ILLITERACY RATE

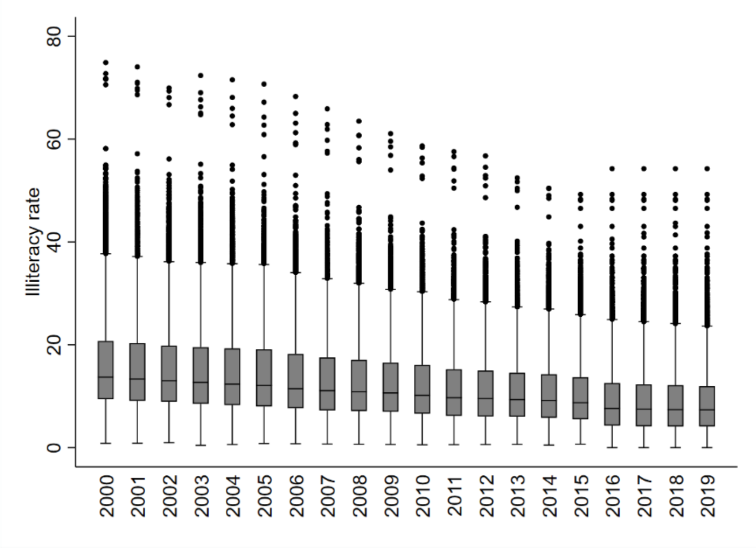

Source: Author's plot for 8,332 selected municipalities in Brazil, Colombia, Ecuador, and Mexico, over 20 years (from 2000 to 2019).
Note: Illiteracy rate is the proportion of individuals older than 15 years who are illiterate. We selected municipalities with adequate quality of civil registration and vital statistics (CRVS). In Brazil, the illiteracy rate was available at the municipal level for the years 2000 and 2010, and the period 2001-09 and 2011-119 were extrapolated. In Colombia, the illiteracy rate was available for the years 2005 and 2018, and we extrapolate the period 2000-2004, and te year 2019. Ecuador has the illiteracy rate available at the municipal level for the years 2001 and 2010, and we extrapolate the period 2002-09, 2011-19, and the year 2000. In Mexico, the illiteracy at the municipal level is available for the years 2000, 2005, 2010, 2015 and 2020, so we extrapolate the years 2001-04, 2006-09, 2011-14, and 2015-19.

FIGURE 4. Sewage boxplot for selected municipalities in Brazil, Colombia, Ecuador, and Mexico (BCEM), from the period 2000-19.

EACH COUNTRY – SEWAGE

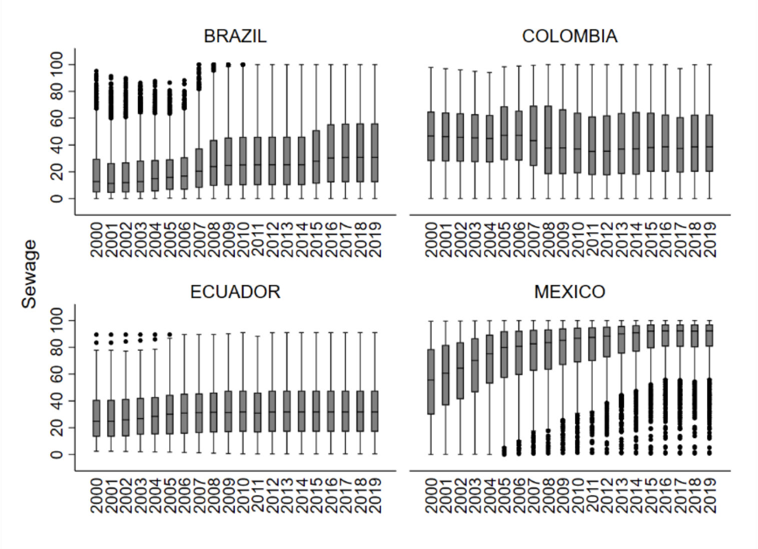

ALL COUNTRIES (BCEM) – SEWAGE

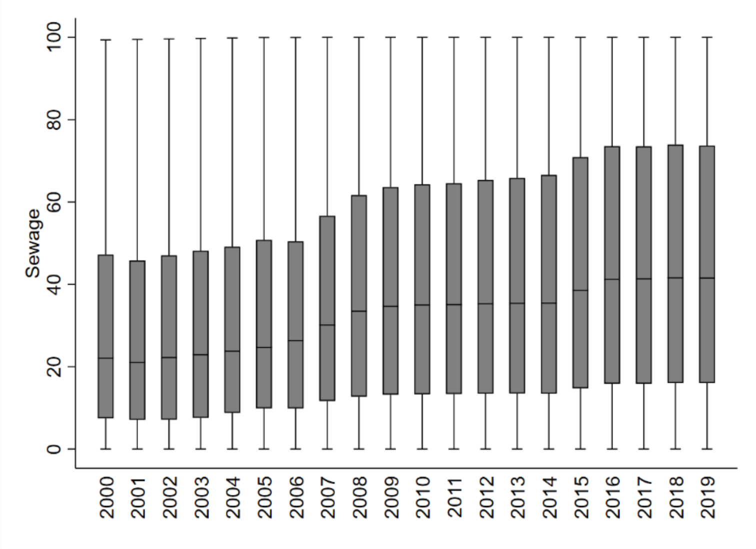

Source: Author's plot for 8,332 selected municipalities in Brazil, Colombia, Ecuador, and Mexico, over 20 years (from 2000 to 2019).
Note: This variable refers is the proportion of individuals living in households with adequate sanitation. We selected municipalities with adequate quality of civil registration and vital statistics (CRVS). In Brazil, the sewage coverage was available at the municipal level for the years 2000 and 2010, and the period 2001-09 and 2011-119 were extrapolated. In Colombia, the sewage coverage was available for the period 2005-2015, so we extrapolate the years 2000-2004 and 2016-2019. Ecuador has the sewage coverage available at the municipal level for the years 2001 and 2010, and we extrapolate the period 2002-09, 255 2011-19, and the year 2000. In Mexico, the sewage at the municipal level is available for the years 2000, 2005, 2010, 2015 and 256 2020, so we extrapolate the years 2001-04, 2006-09, 2011-14, and 2015-19.

FIGURE 5. Piped water boxplot for selected municipalities in Brazil, Colombia, Ecuador, and Mexico, (BCEM), from the period 2000-19.

EACH COUNTRY – PIPED WATER

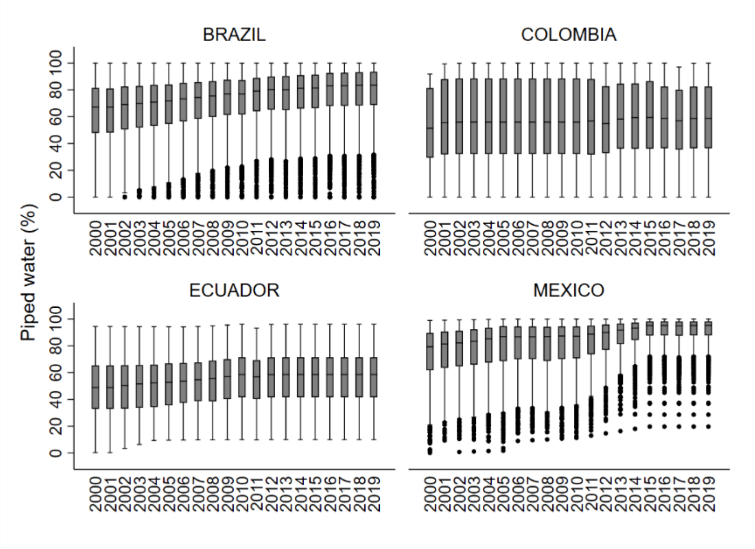

ALL COUNTRIES (BCEM) – PIPED WATER

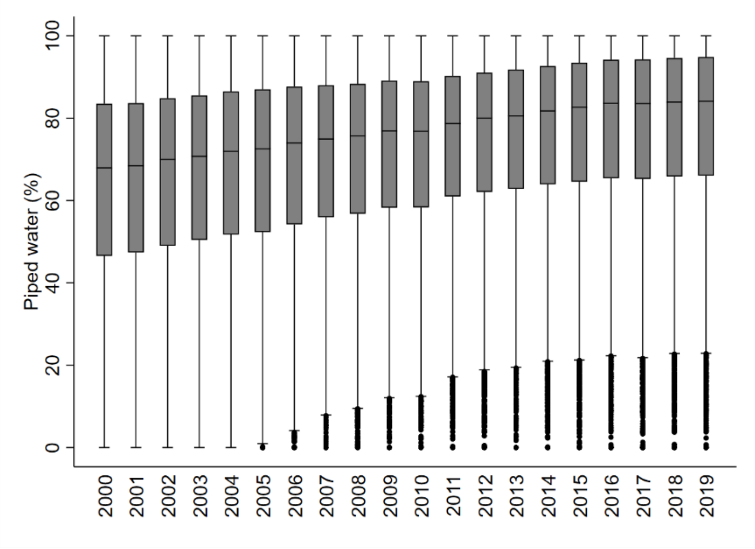

Source: Author's plot for 8,332 selected municipalities in Brazil, Colombia, Ecuador, and Mexico, over 20 years (from 2000 to 2019).
Note: This variable refers is the proportion of individuals living in households with piped water. We selected municipalities with adequate quality of civil registration and vital statistics (CRVS). In Brazil, the piped water was available at the municipal level for the years 2000 and 2010, and the period 2001-09 and 2011-19 were extrapolated. In Colombia, the sewage coverage was available for the period 2005-2015, so we extrapolate the years 2000-2004 and 2016-2019. Ecuador has the piped water coverage available at the municipal level for the years 2001 and 2010, and we extrapolate the period 2002-09, 2011-19, and the year 2000. In Mexico, the piped water at the municipal level is available for the years 2000, 2005, 2010, 2015 and 2020, so we extrapolate the years 2001-04, 2006-09, 2011-14, and 2015-19.

FIGURE 6. Physicians rate boxplot for selected municipalities in Brazil, Colombia, Ecuador, and Mexico (BCEM), and Mexico (BCEM), from the period 2000-19.

EACH COUNTRY – PHYSICIANS RATE

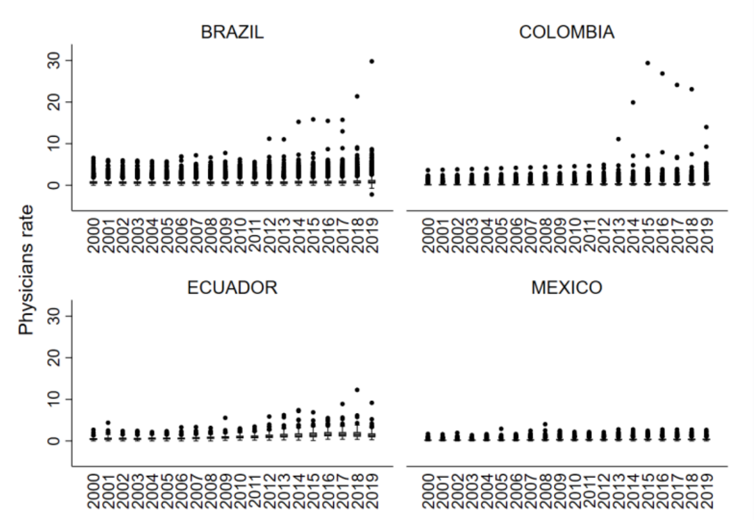

ALL COUNTRIES (BCEM) – PHYSICIANS RATE

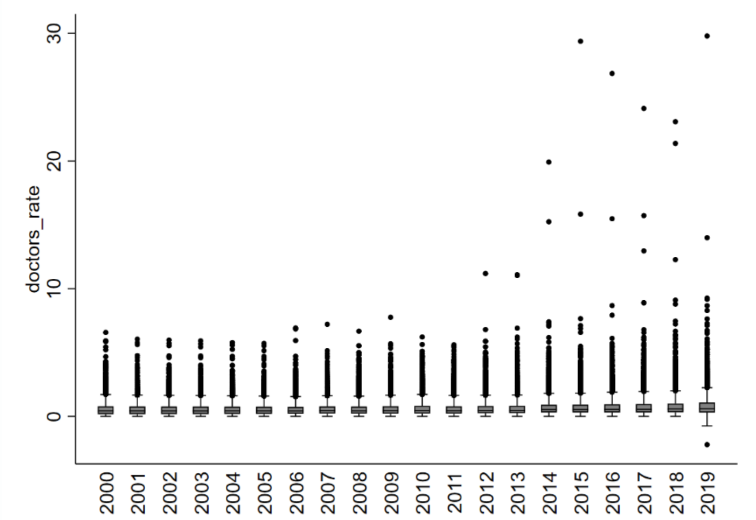

Source: Author's plot for 8,332 selected municipalities in Brazil, Colombia, Ecuador, and Mexico, over 20 years (from 2000 to 2019).
Note: This variable refers to number of physicians per 1,000 inhabitants. We selected municipalities with adequate quality of civil registration and vital statistics (CRVS). In Brazil and Mexico, the physicians' rate was available at the municipal level for the years 2000-19 (no need extrapolations). In Colombia, the physicians' rate was available at the municipal level for the years 2010-19, so we extrapolate for the period 2000-09. Ecuador has the physicians' rate available at the municipal level for the years 2000- 17, and we extrapolate the period 2018-19.

FIGURE 7. Hospital beds rate boxplot for selected municipalities in Brazil, Colombia, Ecuador, and Mexico (BCEM), from the period 2000-19.

EACH COUNTRY – HOSPITAL BEDS RATE

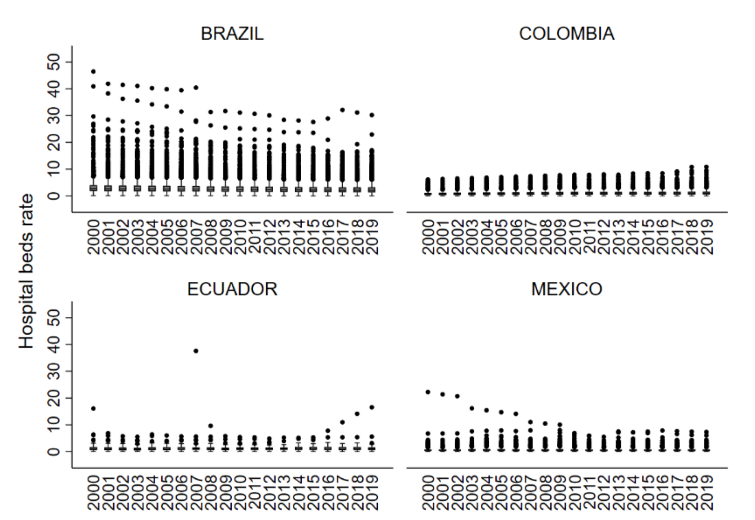

ALL COUNTRIES (BCEM) – HOSPITAL BEDS RATE

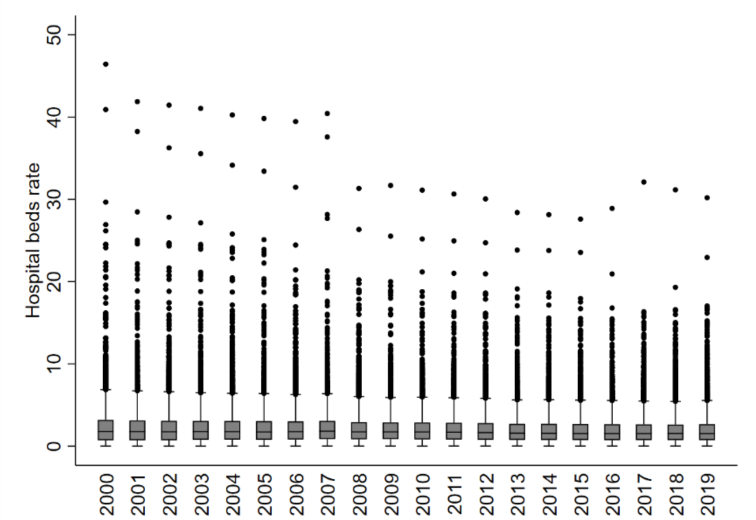

Source: Author's plot for 8,332 selected municipalities in Brazil, Colombia, Ecuador, and Mexico, over 20 years (from 2000 to 2019).
Note: This variable refers to number of hospital beds per 1,000 inhabitants. We selected municipalities with adequate quality of civil registration and vital statistics (CRVS). In Brazil and Mexico, the hospital bed rate was available at the municipal level for the years 2000-19 (no need extrapolations). In Colombia, the physicians' rate was available at the municipal level for the years

2010-19, so we extrapolate for the period 2000-09. Ecuador has the hospital beds rate available at the municipal level for the years 2004-19, and we extrapolate the period 2000-03.

2.3. Some information about poverty rate

In the case of Mexico, the poverty rate is a multidimensional poverty criterion, called "índice de marginación", provided by CONAPO (National Population Council, "*Consejo Nacional de Población*"). In Colombia, the Multidimensional Poverty Index provided by CEDE (Center for Economic Development Studies, "*Centro de Estudios sobre Desarrollo Económico*") was used. In Ecuador, there are different measures for poverty. However, the one that was used was the Unsatisfied Basic Need Index ("*Necesidades Básicas Insatisfechas* - NBI") provided by INEC (National Statistics Institute, "*Instituto Nacional de Estadísticas y Censos*"). In Brazil, we used PNAD (National Household Sample Survey, "*Pesquisa Nacional por Amostra de Domicílios*") microdata to calculate how many people were below the poverty line (originally being ½ of the minimum wage in 2003, and is updated by official decrees), and then obtain the poverty rate.

Therefore, we have different concepts of poverty, with different measures by country. However, the main objective of the poverty rate is to be used as a control variable, and for that purpose, we transformed this rate into a dummy variable, summing a value equal to zero if the municipality is below the poverty median, and equal to 1 if the municipality is above the poverty median. The median was calculated separately by country.

FIGURE 8. Poverty rate boxplot for selected municipalities in Brazil, Colombia, Ecuador, and Mexico (BCEM), from the period 2000-19.

EACH COUNTRY – POVERTY RATE

ALL COUNTRIES (BCEM) – POVERTY RATE

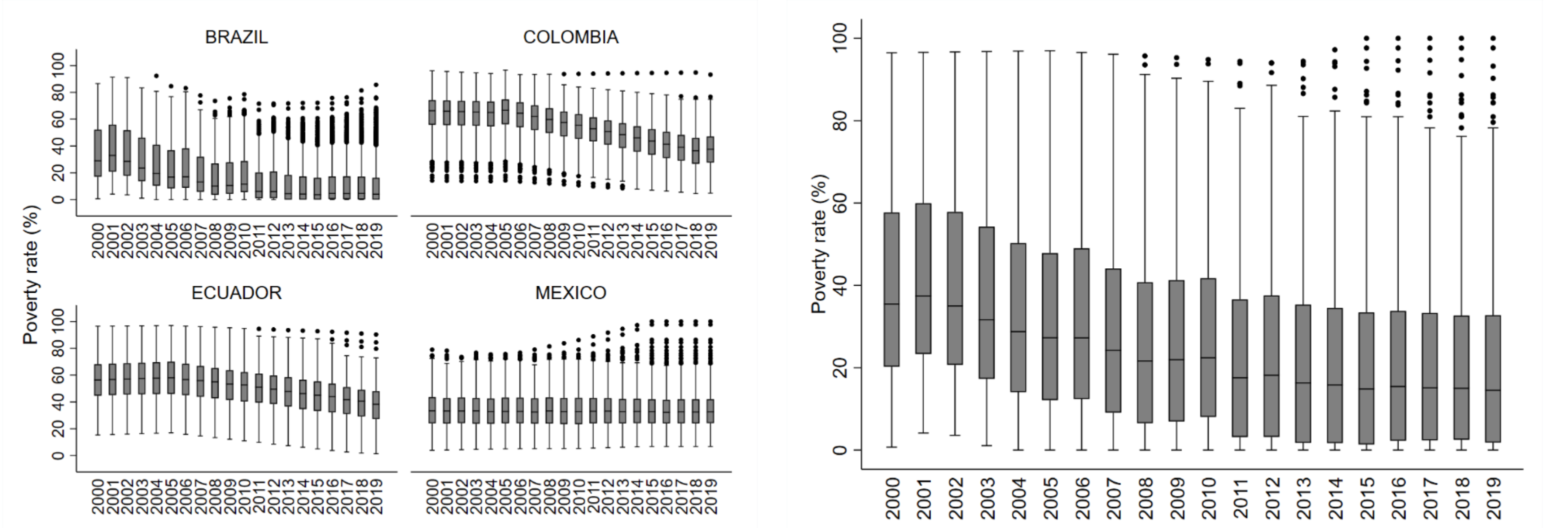

Source: Author's plot for 8,332 selected municipalities in Brazil, Colombia, Ecuador, and Mexico over 20 years (from 2000 to 2019).

Note: We selected municipalities with adequate quality of civil registration and vital statistics (CRVS). In Brazil, the poverty rate was available at the municipal level for the years 2000 and 2010, and the period 2001-09 and 2011-119 were extrapolated with microdata at state level. In Colombia, poverty rate was available for the period 2002-2019, so we extrapolate the years 2000 and 2001. Ecuador has the poverty rate available at the municipal level for the years 2001 and 2010, and we extrapolate the period 2002-09, 2011-19, and the year 2000. In Mexico, the poverty rate at the municipal level is available for the years 2000, 2005, 2010, 2015 and 2020, so we extrapolate the years 2001-04, 2006-09, 2011-14, and 2015-19.

2.4. Quality of Vital Statistics Method

The civil registration and vital statistics (CRVS), despite of being an important public health instrument for planning and evaluation, in most of the developing countries have still low quality and coverage.[25] In that regard, a study[26] involving 148 countries shows that the most places with medium to low quality of CRVS during period 1980-2012 were in African, Asian, and Latin American regions. This reinforces the necessary care and attention of studies that work with CRVS in these regions. However, countries have been encouraged to stimulate the need to monitor progress and accountability of CRVS, especially since 2015 for the Sustainable Development Goals (SDG).[25]

From a methodological point of view, the good quality of these CRVS is also crucial to guarantee the veracity of the results, especially in impact evaluation studies. Since our study uses municipal level child death and livebirth data in Latin American countries, it is essential to separate those municipalities with high and low quality from these CRVS.

To mitigate this, we followed a methodology[27] to calculate the level of quality of CRVS of municipalities, which has been widely used in previous studies in Latin America and Caribbean countries.[28, 29] This methodology used a validated multidimensional criterion based on five indicators: [1] relative mean deviation of the birth-rate; [2] ratio of reported-to-estimated livebirths; [3] age-standardized mortality rate; [4] relative mean deviation of the mortality rate; and [5] proportion of deaths with undetermined causes (Chapter XVIII, ICD-10).[27]

After calculating these indicators, we made a weighted average of them to obtain a final indicator, followed by the division by terciles of the distribution of each country separately, so that the municipalities within the last two terciles were considered to have good quality of CRVS, and the first tercile refers to municipalities with low quality of CRVS.

As the quality CRVS tends to improve in the recent periods, we chose to apply this methodology in the period from 2000 to 2002, as it is the beginning of the historical series worked on this paper.

2.4.1. Discussion and Results – quality of CRVS method

After applying this method to all 8,332 municipalities in BCEM with data available from 2000 to 2019, we selected a subset of 5,647 municipalities that had adequate quality of CRVS, that covers 68% of them in 2002.

Additionally, the application of this method showed important socio-spatial inequalities: In Brazil, the proportion of adequate vital statistics was higher in the Center-South of the country and in the larger municipalities, and lower in many municipalities in the north of the country (Amazon region) and some in the northeast. In Colombia, the municipalities with lowest CRVS were located in the Eastern plains and Amazon region, while the municipalities with the best results in the Andean region. In Ecuador, the lowest results are in the eastern region, especially in the Amazon region, while the municipalities with the highest CRVS are in the Andean region. In Mexico, the best results are in the northern region of the country and the worst are in the southern region. The Table 3 and Figure 8 show these results.

TABLE 3. Number of municipalities and population before and after filter by adequate CRVS.

COUNTRY	NUMBER OF MUNICIPALITIES			BY POPULATION		
	WITH DATA AVALIABLE DURIN 2000-19 PERIOD	FILTER BY ADEQUATE QUALITY OF CRVS (2000-02)	%	TOTAL	FILTER BY ADEQUATE QUALITY OF CRVS (2000-02)	%
Brazil	5,507	3,669	67%	178,135,381	155,200,666	87%
Colombia	1,123	753	67%	41,328,824	35,240,678	85%
Ecuador	221	158	71%	13,232,884	11,202,804	85%
Mexico	1,481	1,067	72%	110,576,814	95,918,270	87%
**BCEM**	8,332	5,647	**68%**	**343,273,903**	**297,562,418**	**86%**

TABLE 3-A. Main differences between the municipalities included and excluded from the retrospective analysis.

Variables	(A) Included	(B) Excluded	Difference (B-A)
**Mortality rate for children younger than 5 years (per 1,000 livebirths)**			
Overall	14·95 (12·91)	15·65 (11·74)	0·70
Neonatal mortality	9·86 (11·56)	10·52 (12·34)	0·66
Post-neonatal mortality	4·79 (7·74)	5·15 (8·30)	0·36
Infant mortality	13·26 (11·57)	15·88 (17·18)	2·62
Toddler mortality	2·29 (4·60)	2·89 (5·42)	0·60
**Primary Health Care coverage (%)**	76·86 (26·32)	85·03 (22·86)	8·17
**Control variables**			
Proportion of households with adequate sanitation (%)	46·01 (31·86)	69·29 (31·05)	17·28
Proportion of households with adequate water (%)	77·49 (21·73)	70·50 (25·03)	-6·99
Proportion of individuals older than 15 years who are illiterate (%)	9·00 (6·53)	13·19 (7·51)	4·19
Proportion of individuals in poverty (%)	18·87 (18·28)	29·69 (20·38)	10·82
GINI index	43·78 (9·09)	46·76 (10·93)	2·98
Hospital bed rate (per 1,000 inhabitants)	1·61 (1·99)	1·18 (1·79)	-0·43
Physicians rate (per 1,000 inhabitants)	0·83 (0·94)	0·54 (0·75)	-0·29

Note: Date are mean (SD). The mortality rates are calculated per 1,000 livebirths.

FIGURE 9. Municipalities according to the quality of vital information.

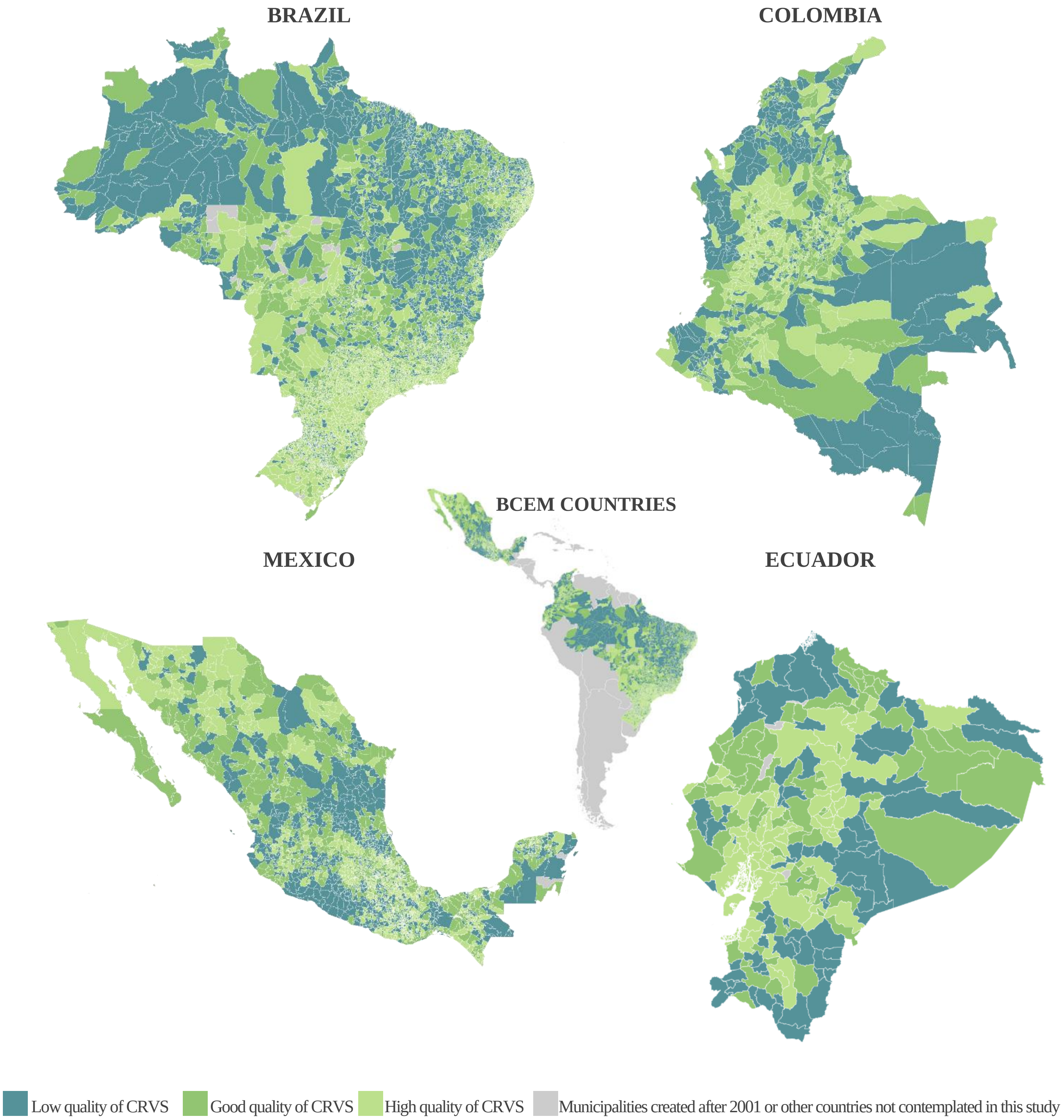

Source: Author's analysis of data from 2000-02 from SIM (DATASUS – Brazil), INEC (Ecuador), and INEGI (Mexico).

PART III - RETROSPECTIVE ANALYSIS

3. Empirical Methods

3.1. Negative binomial regression - Fixed Effects

We estimate Fixed effect models using the negative binomial method to retrospectively evaluate and forecast the impact of each welfare social policy on health outcomes. The equation, that describes the linear relationship between the health outcomes (mortality and hospitalization rates) and covariates, is given by:

$$Log\ Y_{it} = \alpha_i + \sum_{q=1}^{3} \beta_q PHC_{qit} + \sum_{s=4}^{9} \beta_s T_y + \sum_{k=10}^{6} \beta_k X_{kit} + u_{it}$$

Where:

- t refers to the year, i refers to an individual municipality, and q are indexes representing each category of PHC coverage.
- Y_{it} are the different welfare state variables (mortality and hospitalization rates for under-five age group) observed at the municipality i in year t.
- PHC_{qit} are the dummies representing the PHC coverage categories (4 groups) observed at the municipality *i* in year *t* with a coefficient of β_q; with q=0 for low coverage (0-29.9%), q=1 for the intermediate (30-69.9%), q=2 to high (70-99.9%) and q=3 for consolidated coverage (>=100%).
- T_y is the dummy variable that represents precious crisis events with coefficients β_4, β_9, β_{10} from the specific years *y*, with y=2003, 2004, 2008, 2013, 2014, 2015, respectively.
- X_{kit} represents different control covariates, each one with a coefficient of β_k (poverty, Gini index, illiteracy, doctor rates, hospital bed rate, proportion of individuals living in households with inadequate sanitation, and piped water).
- α_i is the fixed effect (time-invariant) term for each municipality, and $u_{it}$is the error term.

4. Results

4.1. Descriptive Analysis

In this section, we describe the temporal dynamics of the PHC programs, alongside with the health outcomes (Under 5 mortality rates by age group and overall) from the year 2000 to 2019. During this period, the overall rate of U5MR reduce expressively.

FIGURE 10. Primary Health Care boxplot for selected municipalities in Brazil, Colombia, Ecuador, and Mexico (BCEM), from the period 2000-19.

EACH COUNTRY – PRIMARY HEALTH CARE | ALL COUNTRIES (BCEM) – PRIMARY HEALTH CARE

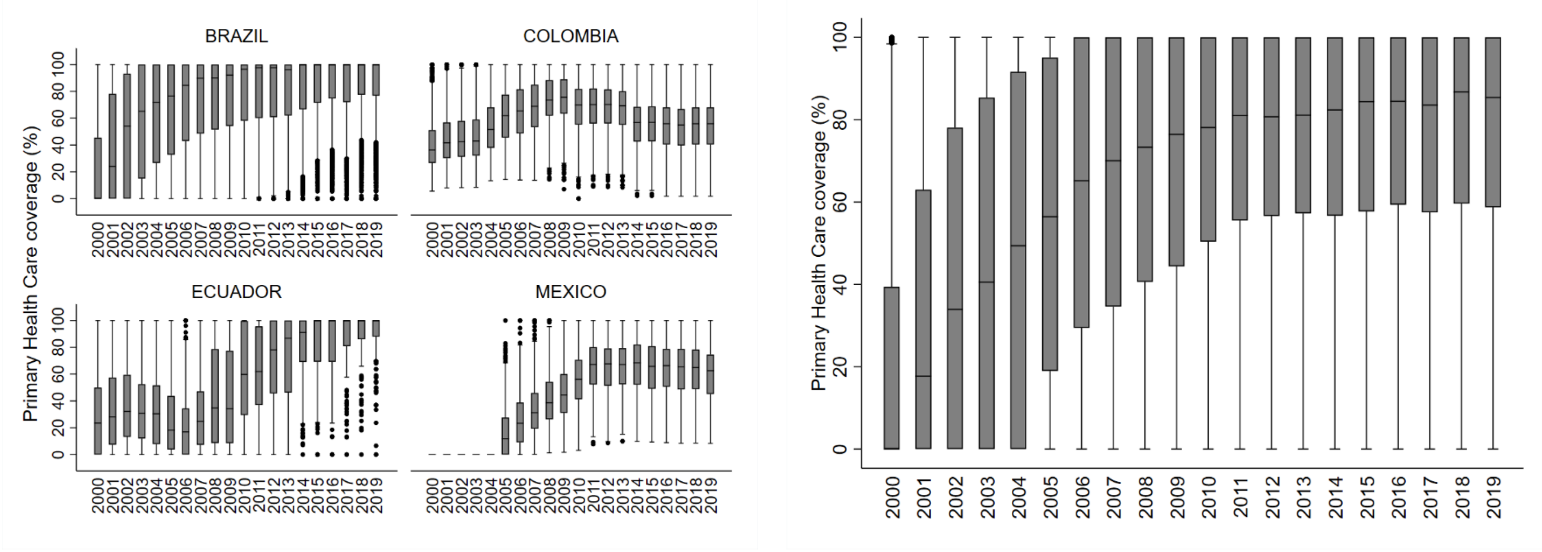

Source: Author's plot for 8,332 selected municipalities in in Brazil, Colombia, Ecuador, and Mexico, over 20 years (from 2000 to 2019).
Note: This variable refers to the Primary Health Care (PHC) programs coverage in relation to the poor population. We selected municipalities with adequate quality of civil registration and vital statistics (CRVS).

FIGURE 11. Child (under 5 years) mortality rate boxplot for selected municipalities in Brazil, Colombia, Ecuador, and Mexico (BCEM), from the period 2000-19.

EACH COUNTRY – CHILD MORTALITY RATE | ALL COUNTRIES (BCEM) – CHILD MORTALITY RATE

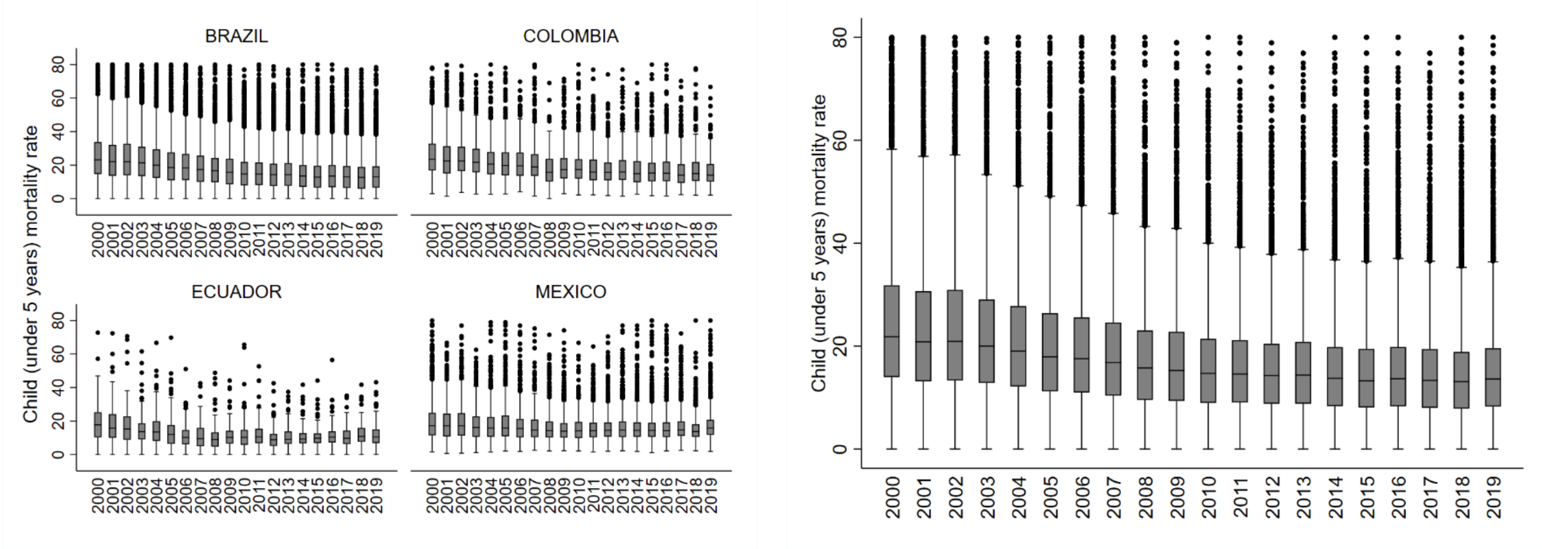

Source: Author's plot for 8,332 selected municipalities in in Brazil, Colombia, Ecuador, and Mexico, over 20 years (from 2000 to 2019).
Note: This variable refers to the child mortality (under-five years) per 1,000 livebirths. We selected municipalities with adequate quality of civil registration and vital statistics (CRVS).

FIGURE 12. Toddler (1 to 4 years) mortality rate boxplot for selected municipalities in Brazil, Colombia, Ecuador, and Mexico (BCEM), from the period 2000-19.

EACH COUNTRY – TODDLER MORTALITY RATE

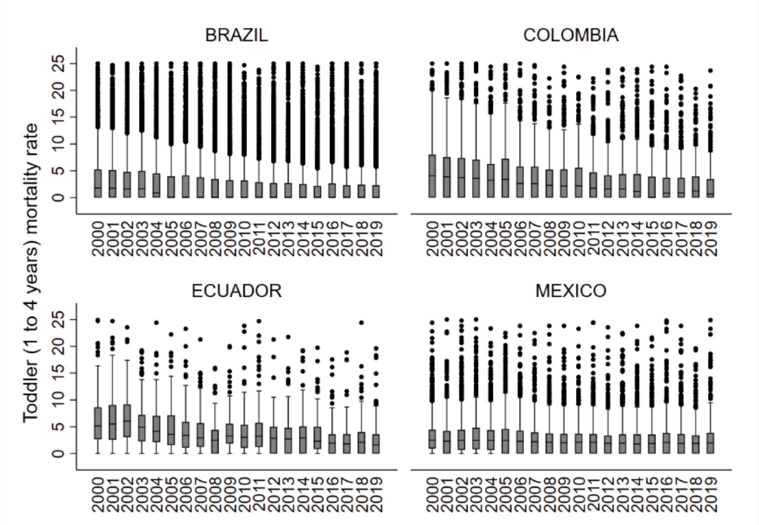

ALL COUNTRIES (BCEM) – TOODLER MORTALITY RATE

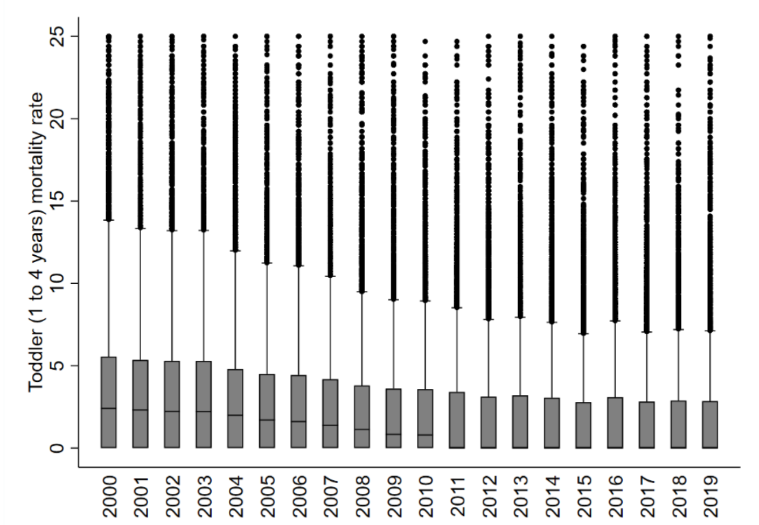

Source: Author's plot for 8,332 selected municipalities in in Brazil, Colombia, Ecuador, and Mexico, over 20 years (from 2000 to 2019).
Note: This variable refers to the toddler mortality (1 to 4 years) per 1,000 livebirths. We selected municipalities with adequate quality of civil registration and vital statistics (CRVS).

FIGURE 13. Infant (under 1 year) mortality rate boxplot for selected municipalities in Brazil, Colombia, Ecuador, and Mexico (BCEM), from the period 2000-19.

EACH COUNTRY – INFANT MORTALITY RATE

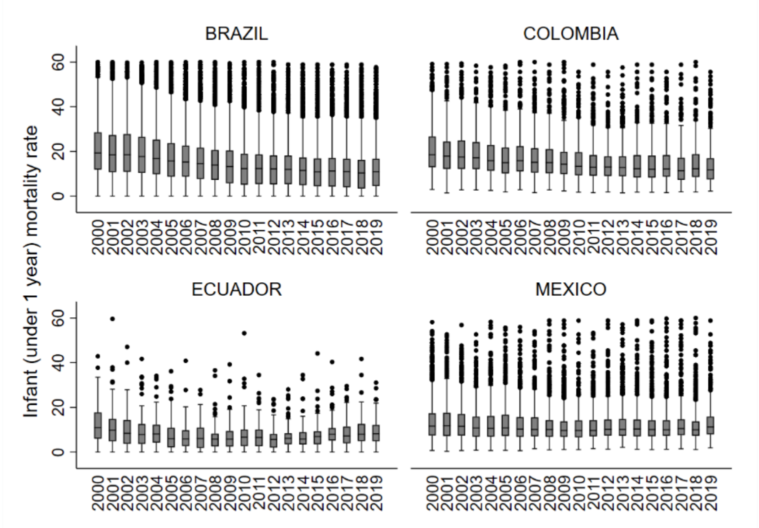

ALL COUNTRIES (BEMC) – INFANT MORTALITY RATE

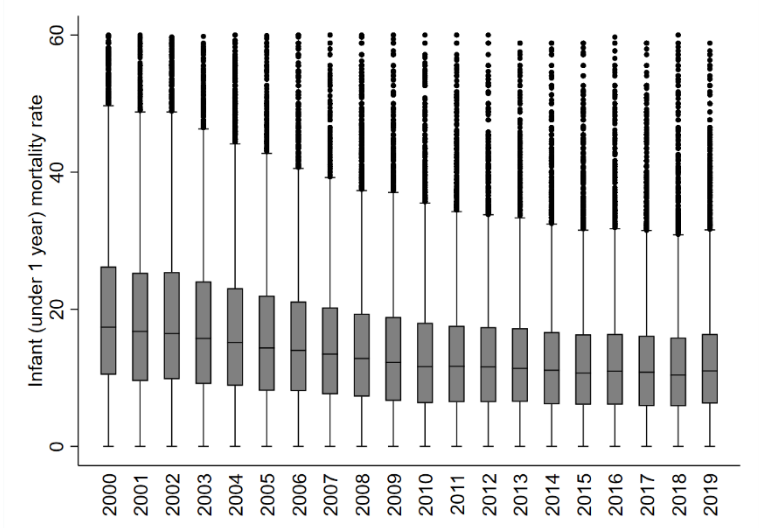

Source: Author's plot for 8,332 selected municipalities in in Brazil, Colombia, Ecuador, and Mexico, over 20 years (from 2000 to 2019).
Note: This variable refers to the infant mortality (under-one year) per 1,000 livebirths. We selected municipalities with adequate quality of civil registration and vital statistics (CRVS).

FIGURE 14. Post-neonatal (28 days to 1 year) mortality rate boxplot for selected municipalities in Brazil, Colombia, Ecuador, and Mexico (BCEM), from the period 2000-19.

EACH COUNTRY – POST-NEONATAL MORTALITY RATE

ALL COUNTRIES (BCEM) – POST-NEONATAL MORTALITY RATE

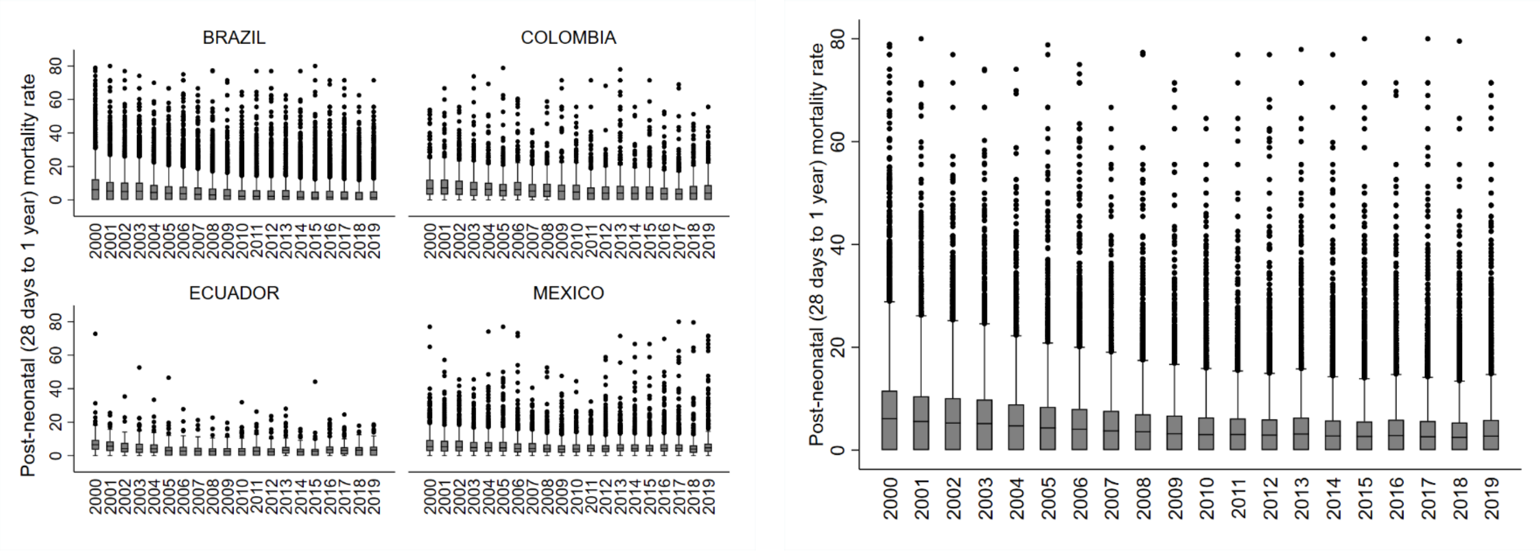

Source: Author's plot for 8,332 selected municipalities in in Brazil, Colombia, Ecuador, and Mexico, over 20 years (from 2000 to 2019).
Note: This variable refers to the post-neonatal mortality (28 days to 1 year) per 1,000 livebirths. We selected municipalities with adequate quality of civil registration and vital statistics (CRVS).

FIGURE 15. Neonatal mortality (0-28 days) rate boxplot for selected municipalities in Brazil, Colombia, Ecuador, and Mexico (BCEM), from the period 2000-19.

EACH COUNTRY – NEONATAL MORTALITY RATE

ALL COUNTRIES (BCEM) – NEONATAL MORTALITY RATE

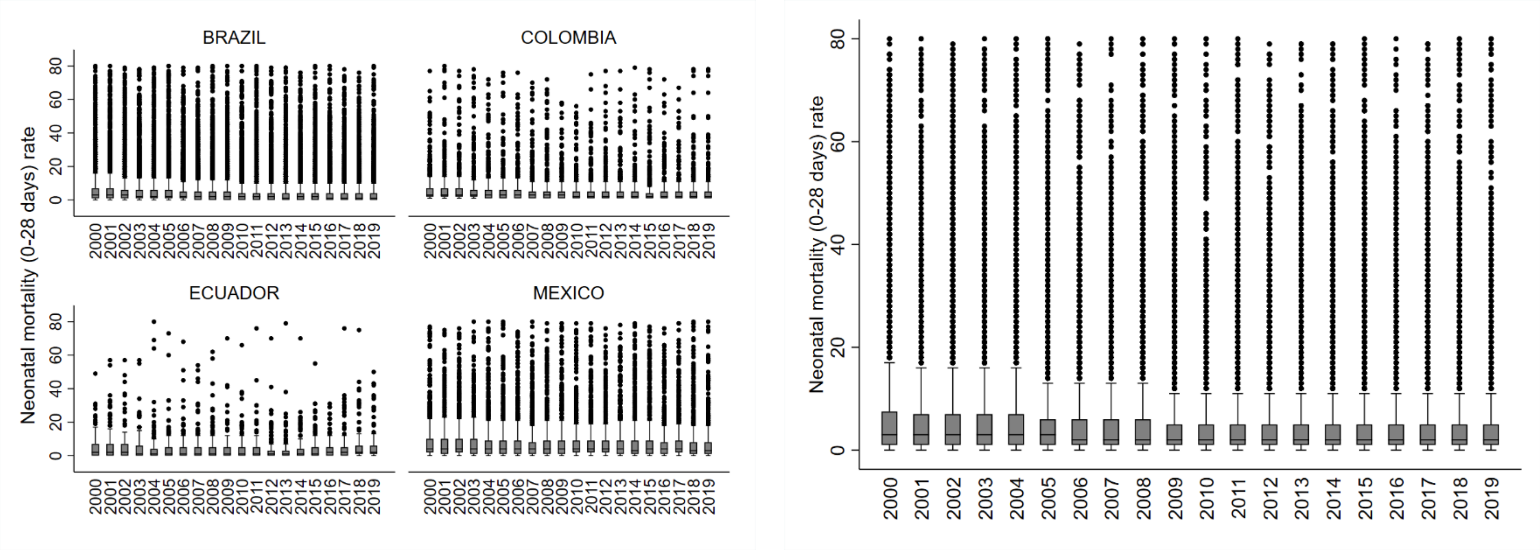

Source: Author's plot for 8,332 selected municipalities in in Brazil, Colombia, Ecuador, and Mexico, over 20 years (from 2000 to 2019).
Note: This variable refers to the neonatal mortality (0 to 28 days) per 1,000 livebirths. We selected municipalities with adequate quality of civil registration and vital statistics (CRVS).

4.2. Triangulation – Difference-in-difference with propensity score matching

We analyzed the effect of PHC on U5MR by difference-in-difference (DID) with propensity score matching (PSM) as a triangulation approach,[30] using the municipalities with no or low PHC coverage since 2005 (n=2,684), versus medium and high coverage municipalities since 2005 (n=274), totaling 2,958 municipalities analyzed in the years 2005 and 2019.

We chose 2005 as the starting year for this analysis because it is the first year of the entire historical series worked on in which data from the 4 PHC programs are available.

In addition, we separated municipalities into low and high PHC coverage according to the coverage quartile of these programs used in the main regression. Thus, municipalities with coverage belonging to the first two quartiles receive a value equal to 0 (control). Similarly, municipalities with coverage in the last two quartiles receive a value equal to 1 (treated).

So that treatment status does not change between treated municipalities, we add a treatment duration effect, i.e., we filter municipalities that have no or low PHC program coverage during the first 6 years (control) and municipalities with medium or high coverage of the PHC program in at least 6 years (treated). We chose the duration period of 6 years because it is the approximate value of the average time elapsed in this part of the analysis (that is, between 2005-2019).

We also tested different duration periods, such as 5 years or more, and 8 years or more. We also tested different ways of estimating the DID, with the "diff" command in STATA and the step-by-step procedure described in the World Bank handbook,[2] which allows estimating DID with a negative binomial panel of fixed effects and with coefficients in rate ratio (RR). We prefer this second one because it allows a more direct comparison with the results reported in the manuscript.

Tables 4 and 5 show the result of DID with PSM, coverages of CCT programs were associated with a statistically significant reduction in child mortality rates, with rate ratios (RR) of 0·990 (95% CI:0.982-0.997).

Even after approaching the municipalities by characteristics observed by the kernel matching method, the first difference in 2005 shows that municipalities with high coverage of the PHC program had a higher infant mortality rate compared to municipalities with low coverage. The second difference, in 2019, shows that both municipalities low and high PHC coverage reduced their child mortality. However, municipalities with high coverage of PHC programs had an even greater reduction, so the difference in difference was statistically significant. Thus, the PHC programs contributed to the reduction of infant mortality, being compatible with the results already found and described in the main manuscript, so these results by DID with PSM reinforce the results found by the fixed effect panel with negative binomial, being a form of triangulation of results.

TABLE 4. **Difference-in-difference with Propensity Score Matching for the association between child mortality rates and intermediate to high Primary Health Care (PHC) coverage, in 2005 and 2019.**

	**Child**<br>Under 5 years	**Toddler**<br>1 to 4 years	**Infant**<br>Under 1 year	**Post Neonatal**<br>28 days to 1 year	**Neonatal**<br>0 to 28 days
Before (2004)					
Control	22·127	6·74	17·964	4·122	11·342
Treated	22·179	7·519	18·986	3·985	11·88
1st Difference (T-C)	0·052	0·779**	1·022*	-0·137	0·538
	(0·737)	(0·364)	(0·621)	(0·217)	(0·414)
After (2019)					
Control	16·325	5·1	13·863	2·686	9·183
Treated	13·812	4·231	12·561	2·61	9·176
2nd Difference (T-C)	-2·513***	-0·869**	-1·302**	-0·075	-0·007
	(0·746)	(0·378)	(0·647)	(0·226	(0·431)
**Diff-in-Diff**	**-2·566**<br>(1·048)**	**-1·648***<br>(0·525)**	**-2·324***<br>(0·897)**	**0·061<br>(0·314)**	**-0·545<br>(0·598)**

Source: Author's data analysis for 5,224 observations – 2,612 municipalities in Brazil, Colombia, Ecuador and Mexico, in 2005 and 2019 period.

Note: Data are in mortality rate per 1,000 livebirths, with standard errors in parentheses. The symbols '***', '**' and '*' denote significance at 1%, 5%, and 10% respectively. We use the STATA "diff" command, with kernel matching (PSM) to approximate the compared municipalities according to their observable characteristics.

TABLE 5. Rate Ratios from the difference-in-difference fixed effect negative binomial models for the association between U5MR and different duration for Primary Health Care (PHC) coverage.

	**Low PHC coverage (first two quartiles) *versus* Intermediate or more (last two quartiles), by duration**		
	5 years	6 years	8 years
**PHC coverage (dummy)**			
	1·22424e+11***	-	-
	[68904·245-2·175e+17]	-	-
	-	302896·8**	-
	-	[4·543-2·019e+10]	-
	-	-	9·76330e+09***
	-	-	[1269·376-7·509e+16]
Intermediate to high (first two quartiles) versus low (last two quartiles) for at least 5 years	0·988***	-	-
	[0·982-0·995]	-	-
Intermediate to high (first two quartiles) versus low (last two quartiles) for at least 6 years	-	0·995*	-
	-	[0·990-1·000]	-
Intermediate to high (first two quartiles) versus low (last two quartiles) for at least 8 years	-	-	0·990***
	-	-	[0·982-0·997]
**Control variables**			
Poverty rate (%)	1·012***	1·014***	1·012***
	[1·007-1·018]	[1·012-1·016]	[1·007-1·018]
Proportion of individuals older than 15 years who are illiterate (%)	1	0·997	0·994
	[0·988-1·013]	[0·992-1·002]	[0·981-1·008]
Gini Index	1·006	1·006***	1·015***
	[0·998-1·015]	[1·003-1·009]	[1·006-1·023]
Piped water	1	1·004***	1·004**
	[0·998-1·003]	[1·003-1·006]	[1·001-1·007]
Proportion of individuals living in households with inadequate sanitation	0·998	0·994***	0·997*
	[0·994-1·001]	[0·993-0·996]	[0·993-1·000]
Hospital bed rate per 1,000 population (%)	1·052***	1·027***	1·026
	[1·018-1·088]	[1·013-1·041]	[0·995-1·059]
Rate of physicians per 1,000 population (%)	0·999	0·888***	0·951
	[0·918-1·088]	[0·871-0·905]	[0·869-1·041]
_cons	0·0134***	0·00852***	0·00629***
	[0·007-0·026]	[0·007-0·011]	[0·003-0·012]
**Number of observations**	5,224	5,362	936
**Number of municipalities**	2,612	2,681	468

Source: Author's data analysis for 5,224 observations – 2,612 municipalities in Brazil, Colombia, Ecuador and Mexico, in 2005 and 2019 period.
Note: Data are in Rate Ratio (RR) coefficients (95% CI) unless otherwise specified. The confidence intervals are in brackets. The symbols '***' , '**' and '*' denote significance at 1%, 5%, and 10% respectively.

4.3. Fit and sensitivity tests

We performed several fitting and sensitivity tests to ensure the robustness of the findings presented in this study.

Initially, we performed the Hausman test to choose between the Fixed and Random Effect models, and the former was deemed more appropriate (see Table 6). Subsequently, we assessed the goodness of fit using the Log-likelihood, AIC, and BIC. These measures consistently demonstrated that the adjusted models produced better results compared to the unadjusted models (see Table 6).

Second, we modified the model specification by measuring the crude estimates of Rate Ratios without including any covariables, and excluding some variables (see Table 7).

Third, we examined the impact of different categorization approaches by estimating the models with continuous variables (see Table 8), and changing from quartile to fixed categories (see Table 9).

Fourth, in order to assess the external validity of our estimates we extended the analysis to include all 8,332 municipalities in the BCEM dataset. This approach encompassed municipalities with vital information of potentially lower quality (see Table 10).

Fifth, we compared the overall results obtained using the Negative Binomial regression to that from Poisson regressions using the Log-likelihood, AIC, and BIC information criteria (see Table 11). The test results showed that the Negative Binomial method yields better estimates. This is reasonable since the negative binomial regression takes the data overdispersion into account, compared to the Poisson which does not.

Sixth, we show the regression results by countries (see Table 12, 13,14).

In other words, given that our results withstood the vast number of sensitivity tests performed, we conclude that the results and conclusions drawn in this study are robust and stable.

TABLE 6. Hausman test between fixed effect and random effect negative binomial models for the association between Primary Health care coverage and under-five mortality rates, in BCEM, 2000-2019.

	Mortality		Mortality	
	**Fixed effects**	**Random effects**	**With controls**	**Without controls**
N. of observations<br>(n. of variables)	108,386<br>(14)	108,388<br>(14)	108,386<br>(14)	109,638<br>(2)
Log likelihood	-228903·8	-254999.4	-228903.8	-235327.2
Akaike information criterion (AIC)	457839·6	510034.8	457839.6	470662.4
Bayesian information criterion (BIC)	457993·1	510207.5	457993.1	470700.9
**Hausman test**	χ^2**=1744·1;** p**-value= 0·000**			

TABLE 7. Fixed-effect negative binomial models for association between Primary Health Care coverage and under-five mortality rates for omitted variables bias test, in BCEM, 2000–2019.

Variables	Crude regression	Without time control	Without selected variables	All variables
**Primary Health Care coverage (%)**				
Low (0 to <34·3%)	1·00	1·00	1·00	1·00
Intermediate (≥34·3 to <70·3%)	0·838***<br>(0·836-0·842)	0·878***<br>(0·874-0·883)	0·867***<br>(0·862-0·872)	0·888***<br>(0·883-0·893)
High (≥70·3% to <100%)	0·766***<br>(0·760-0·772)	0·831***<br>(0·824-0·838)	0·815***<br>(0·809-0·822)	0·843***<br>(0·837-0·851)
Consolidate (100%)	0·701***<br>(0·694-0·709)	0·799***<br>(0·790-0·808)	0·785***<br>(0·777-0·794)	0·814***<br>(0·805-0·822)
**Control variables**				
Proportion of households with adequate sanitation (%)		0·894***<br>(0·886-0·902)	0·896***<br>(0·888-0·904)	0·901***<br>(0·893-0·909)
Proportion of households with adequate water (%)		0·985***<br>(0·976-0·994)	0·976***<br>(0·967-0·985)	0·991*<br>(0·982-1·000)
Proportion of individuals older than 15 years who are illiterate		1·102***<br>(1·091-1·113)	1·122***<br>(1·111-1·133)	1·094***<br>(1·083 - 1·105)
Proportion of individuals in poverty (%)		1·263***<br>(1·252-1·273)	1·267***<br>(1·257-1·278)	1·252***<br>(1·242 - 1·263)
GINI index		1·108***<br>(1·099-1·116)		1·099***<br>(1·091-1·107)
Hospital bed rate (per 1,000 inhabitants)		0·993*<br>(0·982-1·005)	0·999*<br>(0·988-1-010)	0·993*<br>(0·982-1·005)
Physicians rate (per 10,000 inhabitants)		0·985**<br>(0·976-0·994)	0·982**<br>(0·973-0·991)	0·993*<br>(0·984-1·002)
**Time trend control**				
2004				1·047***<br>(1·039-1·056)
2007				0·996*<br>(0·987-1·005)
2008				0·977***<br>(0·969-0·986)
2015				0·908<br>(0·899-0·917)*
2018				0·914**<br>(0·905-0·923)
**Number of observations**	**110,390**	**109,080**	**109,739**	**109,080**
**Number of municipalities**	**5,643**	**5,576**	**5,609**	**5,576**

Note: Data are in Rate Ratio (RR) coefficients (95% CI) unless otherwise specified. The confidence intervals are in parentheses.

Time shocks are controls for specific years of economic crisis (2007, 2008, 2015, and 2018) and for specific yeats related to PHC (2004 and 2008). The symbols '***' , '**' and '*' denote significance at 1%, 5%, and 10% respectively.

TABLE 8. Fixed-effect negative binomial models for association between Primary Health Care coverage and age-specific mortality rates using continuous variables, in BCEM, 2000-2019.

**Variables**	**Child Under 5 years**	**Toddler 1 to 4 years**	**Infant Under 1 year**	**Post-neonatal 28 days to 1 year**	**Neonatal 0 to 28 days**
Primary Health Care coverage (%)	0·748*** (0·740-0·756)	0·738*** (0·721-0·756)	0·784*** (0·774-0·794)	0·677*** (0·665-0·689)	0·788*** (0·777-0·799)
**Control variables**					
Proportion of households with adequate sanitation (%)	0·907*** (0·899-0·915)	0·934*** (0·912-0·949)	0·928*** (0·918-0·937)	0·873 (0·860-0·886)	0·924*** (0·913-0·934)
Proportion of households with adequate water (%)	1·002 (0·993-1·011)	1·016 (0·996-1·036)	0·972*** (0·962-0·982)	0·967** (0·952-0·981)	1·017* (1·005-1·028)
Proportion of individuals older than 15 years who are Illiterate (%)	1·091*** (1·081 - 1·102)	1·094*** (1·071-1·118)	1·135*** (1·123-1·148)	1·081*** (1·063-1·099)	1·076*** (1·063-1·090)
Proportion of individuals in poverty (%)	1·247*** (1·237 - 1·257)	1·252*** (1·229-1·276)	1·300*** (1·288-1·313)	1·265*** (1·248-1·283)	1·239*** (1·227-1·252)
GINI index	1·092** (1·084-1·100)	0·987 (0·967-1·006)	1·057** (1·048-1·066)	1·091** (1·078-1·105)	1·087** (1·077-1·097)
Hospital bed rate (per 1,000 inhabitants)	0·993* (0·982-1·004)	0·995* (0·970-1·019)	0·951** (0·939-0·963)	1·003 (0·984-1·022)	0·985** (0·971-0·999)
Physicians rate (per 10,000 inhabitants)	1·000 (0·990-1·009)	0·978*** (0·959-0·997)	0·955*** (0·945-0·965)	0·995* (0·980-1·010)	1·009 (0·997-1·021)
Time trend	Yes	Yes	Yes	Yes	Yes
**Number of observations**	**109,080**	**103,234**	**106,544**	**105,699**	**105,170**
**Number of municipalities**	**5,576**	**5,363**	**5,575**	**5,526**	**5,572**

Note: Data are in Rate Ratio (RR) coefficients (95% CI) unless otherwise specified. The confidence intervals are in parentheses. Time shocks are controls for specific years of economic crises (2007, 2008, 2015, and for specific years related to PHC (2004-2008). The symbols '***' , '**' and '*' denote significance at 1%, 5%, and 10% respectively.

TABLE 9. Fixed-effect negative binomial models for association between Primary Health Care coverage and age-specific mortality rates, using fixed PHC categories, in BCEM, 2000-2019.

**Variables**	**Child**<br>**Under 5 years**	**Toddler**<br>**1 to 4 years**	**Infant**<br>**Under 1 year**	**Post-Neonatal**<br>**28 days to 1 year**	**Neonatal**<br>**0 to 28 days**
**Primary Health Care coverage (%)**					
Low (0-29·9)	1·00	1·00	1·00	1·00	1·00
Intermediate (30-69·9)	0·888***<br>(0·883-0·893)	0·879***<br>(0·869-0·890)	0·883***<br>(0·877-0·888)	0·871***<br>(0·863-0·880)	0·897***<br>(0·891-0·903)
High (70-99·9)	0·858***<br>(0·850-0·866)	0·807***<br>(0·792-0·823)	0·878***<br>(0·869-0·887)	0·811***<br>(0·799-824)	0·891***<br>(0·881-0·902)
Consolidate (100)	0·796***<br>(0·786-0·807)	0·707***<br>(0·687-0·727)	0·844***<br>(0·831-0·856)	0·680***<br>(0·665-0·696)	0·865***<br>(0·851-0·880)
**Control Variables**					
Proportion of households with adequate sanitation (%)	0·902***<br>(0·893-0·910)	0·914***<br>(0·896-0·932)	0·928***<br>(0·919-0·938)	0·869***<br>(0·856-0·882)	0·918***<br>(0·908-0·929)
Proportion of households with adequate water (%)	0·993***<br>(0·985-1·002)	1·008<br>(0·988-1·028)	0·964***<br>(0·954-0·974)	0·959***<br>(0·945-0·974)	1·006<br>(0·995-1·018)
Proportion of individuals older than 15 years who are illiterate (%)	1·099***<br>(1·088 - 1·110)	1·118***<br>(1·095-1·1·142)	1·129***<br>(1·116-1·141)	1·094***<br>(1·076-1·112)	1·082***<br>(1·069-1·096)
Proportion of individuals in poverty (%)	1·254***<br>(1·244 - 1·265)	1·296***<br>(1·273-1·321)	1·320***<br>(1·308-1·332)	1·267***<br>(1·250-1·285)	1·250***<br>(1·237-1·262)
GINI index	1·109***<br>(1·101-1·118)	1·084***<br>(1·066-1·102)	1·031***<br>(1·022-1·040)	1·118***<br>(1·104-1·132)	1·100***<br>(1·090-1·111)
Hospital bed rate (per 1,000 inhabitants)	0·994*<br>(0·983-1·006)	1·002<br>(0·978-1·027)	0·944**<br>(0·932-0·956)	1·004<br>(0·986-1·024)	0·988*<br>(0·974-1·002)
Physicians rate (per 10,000 inhabitants)	0·990**<br>(0·981-0·999)	0·972**<br>(0·953-0·991)	0·940<br>(0·930-0·950)	0·984**<br>(0·969-0·999)	1·000<br>(0·989-1·012)
Years binaries	Yes	Yes	Yes	Yes	Yes
**Number of observations**	109,080	103,234	106,527	105,699	105,170
**Number of municipalities**	5,576	5,363	5,575	5,526	5,572

Note: Data are in Rate Ratio (RR) coefficients (95% CI) unless otherwise specified. The confidence intervals are in parentheses. Time shocks are controls for specific years of economic crisis (2007, 2008, 2015, and 2018) and for specific years related to PHC (2004-2008). The symbols '***' , '**' and '*' denote significance at 1%, 5%, and 10% respectively.

TABLE 10. Fixed-effect negative binomial models for association between Primary Health Care coverage and age-specific mortality rates without filter for adequate quality of information (QVI), in al municipalities in BCEM, 2000-2019

**Variables**	**Child**<br>**Under 5 years**	**Toddler**<br>**1 to 4 years**	**Infant**<br>**Under 1 year**	**Post-Neonatal**<br>**28 days to 1 year**	**Neonatal**<br>**0 to 28 days**
**Primary Health Care coverage (%)**					
Low (0 to <34·3%)	1	1	1	1	1
	(1·000–1·000)	(1·000–1·000)	(1·000–1·000)	(1·000–1·000)	(1·000–1·000)
Intermediate (≥34·3 to <70·3%)	0·894***	0·881***	0·888***	0·869***	0·901***
	(0·890–0·899)	(0·871–0·891)	(0·882–0·893)	(0·861–0·876)	(0·895–0·907)
High (≥70·3% to <100%)	0·882***	0·835***	0·907***	0·829***	0·922***
	(0·875–0·889)	(0·822–0·849)	(0·899–0·915)	(0·819–0·840)	(0·913–0·931)
Consolidate (100%)	0·825***	0·733***	0·875***	0·708***	0·913***
	(0·816–0·834)	(0·717–0·750)	(0·864–0·886)	(0·695–0·721)	(0·900–0·925)
**Control Variables**					
Proportion of households with adequate sanitation (%)	0·880***	0·921***	0·939***	0·839***	0·924***
	(0·872–0·888)	(0·905–0·938)	(0·931–0·948)	(0·827–0·851)	(0·914–0·934)
Proportion of households with adequate water (%)	0·967***	1·015	0·932***	0·934***	0·978***
	(0·959–0·976)	(0·997–1·033)	(0·923–0·941)	(0·921–0·947)	(0·967–0·989)
Proportion of individuals older than 15 years who are illiterate (%)	1·089***	1·118***	1·120***	1·097***	1·073***
	(1·079–1·100)	(1·097–1·140)	(1·108–1·131)	(1·080–1·113)	(1·060–1·085)
Proportion of individuals in poverty (%)	1·198***	1·274***	1·302***	1·268***	1·212***
	(1·187–1·208)	(1·252–1·297)	(1·290–1·314)	(1·251–1·285)	(1·200–1·225)
Gini Index	1·108***	1·064***	1·038***	1·112***	1·106***
	(1·101–1·116)	(1·048–1·080)	(1·030–1·046)	(1·099–1·124)	(1·097–1·115)
Hospital bed rate (per 1,000 inhabitants)	1·007	1·012	0·933***	1·001	0·986**
	(0·996–1·017)	(0·991–1·034)	(0·923–0·944)	(0·984–1·017)	(0·973–0·999)
Physicians rate (per 10,000 inhabitants)	0·998	0·980**	0·949***	0·972***	1·003
	(0·990–1·005)	(0·965–0·996)	(0·941–0·957)	(0·960–0·984)	(0·993–1·012)
Years binaries	Yes	Yes	Yes	Yes	Yes
**Number of observations**	159,612	150,701	155,890	154,954	154,608
**Number of municipalities**	8,199	7,866	8,194	8,096	8,189

Note: Data are in Rate Ratio (RR) coefficients (95% CI) unless otherwise specified. The confidence intervals are in parentheses. Time shocks are control for specific years of economic crisis (2007, 2008, 2015, and 2018) and for specific years related to PHC programs (2004-2008). The symbols '***' , '**' and '*' denote significance at 1%, 5%, and 10% respectively.

TABLE 11. Fixed-effect negative binomial and Poisson models for association between Primary Health Care coverage and age-specific mortality rates, in BCEM, 2000-2019.

**Variables**	**Child**		**Infant**	
	**Negative binomial Rate Ratio (95% CI)**	**Poisson Rate Ratio (95% CI)**	**Negative Binomial Rate Ratio (95% CI)**	**Poisson Rate Ratio (95% CI)**
**Primary Health Care coverage (%)**				
Low (0 to <34·3%)	1·00	1·00	1·00	1·00
Intermediate (≥34·3 to <70·3%)	0·888*** (0·883-0·893)	0·883*** (0·879-0·887)	0·866*** (0·861-0·872)	0·848*** (0·844-0·852)
High (≥70·3% to <100%)	0·843*** (0·837-0·851)	0·823*** (0·817-0·828)	0·863*** (0·855-0·871)	0·807*** (0·801-0·813)
Consolidate (100%)	0·814*** (0·805-0·822)	0·769*** (0·762-0·775)	0·864*** (0·853-0·875)	0·753*** (0·746-0·761)
**Control variables**				
Proportion of households with adequate Sanitation (%)	0·901*** (0·893-0·909)	0·915*** (0·909-0·922)	0·919*** (0·909-0·928)	0·893*** (0·886-0·900)*
Proportion of households with adequate water (%)	0·991* (0·982-1·000)	0·995* (0·988-1·003)	0·960*** (0·950-0·970)	1·009* (1·001-1·017)
Proportion of individuals older than 15 years who are illiterate (%)	1·094*** (1·083 - 1·105)	1·084*** (1·075 - 1·092)	1·129*** (1·117-1·142)	1·065*** (1·056-1·074)
Proportion of individuals in poverty (%)	1·252*** (1·242 - 1·263)	1·262*** (1·253 - 1·270)	1·306*** (1·294-1·318)	1·281*** (1·272-1·290)
GINI index	1·099*** (1·091-1·107)	1·102*** (1·095-1·109)	1·057*** (1·048-1·066)	1·089*** (1·081-1·096)
Hospital bed rate (per 1,000 inhabitants)	0·993* (0·982-1·005)	1·002 (0·992-1·011)	0·953*** (0·941-0·965)	1·014* (1·004-1·025)
Physicians rate (per 10,000 inhabitants)	0·993* (0·984-1·002)	0·998* (0·991-1·005)	0·951*** (0·941-0·961)	0·998* (0·990-1·007)
Years binaries	Yes	Yes	Yes	Yes
**Number of observations**	**109,080**	**109,080**	**106,544**	**106,544**
**Number of municipalities**	**5,576**	**5,576**	**5,575**	**5,575**
**Akaike´s information criterion (AIC)**	**460,837**	**472,202**	**446,448**	**466,906**
**Bayesian information criterion (BIC)**	**460,990**	**472,346**	**446,601**	**467,049**

Note: Data are in Rate Ratio (RR) coefficients (95% CI) unless otherwise specified. The confidence intervals are in parentheses. Time shocks are controls for specific years of economic crisis (2007, 2008, 2015, and 2018) and for specific years related to PHC (2004-2008). The symbols '***' , '**' and '*' denote significance at 1%, 5%, and 10% respectively.

TABLE 12. Fixed-effect negative for association between Primary Health Care coverage and under-five mortality rate by countries, in BCEM, 2000-2019.

Variables	Child mortality (0-5 years)			
	**Brazil**	**Colombia**	**Ecuador**	**Mexico**
**Primary Health Care coverage (%)**				
Low (0 to <34·3%)	1·00	1·00	1·00	1·00
Intermediate (≥34·3 to <70·3%)	0·851***<br>(0·845-0·857)	0·832***<br>(0·816-0·848)	0·938***<br>(0·905-0·972)	0·898***<br>(0·889-0·907)
High (≥70·3% to <100%)	0·794***<br>(0·785-0·802)	0·711***<br>(0·690-0·732)	0·810***<br>(0·777-0·844)	0·910*<br>(0·894-0·927)
Consolidate (100%)	0·745***<br>(0·735-0·755)	0·664***<br>(0·637-0·692)	0·881**<br>(0·838-0·928)	0·975<br>(0·945-1·006)
**Control variables**				
Proportion of households with adequate sanitation (%)	0·862***<br>(0·854-0·871)	1·113*<br>(1·082-1·145)	0·851***<br>(0·744-0·973)	1·037*<br>(1·002-1·074)
Proportion of households with adequate water (%)	0·951***<br>(0·940-0·962)	0·973***<br>(0·952-0·994)	0·619***<br>(0·547-0·700)	1·069<br>(1·047-1·092)
Proportion of individuals older than 15 years who are illiterate (%)	1·028***<br>(1·015 - 1·041)	1·226***<br>(1·198-1·254)	1·129***<br>(1·058-1·205)	1·089***<br>(1·065-1·114)
Proportion of individuals in poverty (%)	1·197***<br>(1·185-1·209)	1·389***<br>(1·363-1·415)	1·111***<br>(1·017-1·214)	1·130***<br>(1·088-1·175)
GINI index	1·121***<br>(1·109-1·133)	1·050***<br>(1·020-1·082)	1·032*<br>(0·944-1·128)	1·083***<br>(1·069-1·097)
Hospital bed rate (per 1,000 inhabitants)	1·020***<br>(1·005-1·034)	0·857***<br>(0·826-0·889)	0·867***<br>(0·808-0·930)	0·986*<br>(0·964-1·009)
Physicians rate (per 10,000 inhabitants)	0·985**<br>(0·974-0·995)	0·820***<br>(0·790-0·851)	0·906***<br>(0·857-0·857)	1·040*<br>(1·020-1·061)
Years binaries	Yes	Yes	Yes	Yes
Number of observations	73,358	13,185	2,799	19,044
Number of municipalities	3,668	694	149	1,065

Note: Data are in Rate Ratio (RR) coefficients (95% CI) unless otherwise specified. The confidence intervals are in parentheses. Time shocks are controls for specific years of economic crisis (2007, 2008, 2015, and 2018) and for specific years related to PHC (2004-2008). The symbols '***' , '**' and '*' denote significance at 1%, 5%, and 10% respectively.

TABLE 13. Fixed-effect negative for association between Primary Health Care coverage and toddler mortality rate by countries, in BCEM, 2000-2019.

Variables	Toddler mortality (1-4 years)			
	**Brazil**	**Colombia**	**Ecuador**	**Mexico**
**Primary Health Care coverage (%)**				
Low (0 to <34·3%)	1·00	1·00	1·00	1·00
Intermediate (≥34·3 to <70·3%)	0·837*** (0·822-0·851)	0·848*** (0·814-0·883)	0·960* (0·908-1·014)	0·931*** (0·911-0·951)
High (≥70·3% to <100%)	0·780*** (0·761-0·800)	0·695*** (0·655-0·737)	0·701*** (0·654-0·751)	0·866*** (0·837-0·897)
Consolidate (100%)	0·719*** (0·698-0·741)	0·624*** (0·576-0·677)	0·696*** (0·641-0·756)	0·893** (0·844-0·946)
**Control variables**				
Proportion of households with adequate sanitation (%)	0·848*** (0·829-0·869)	1·137* (1·074-1·205)	0·882* (0·726-1·072)	1·042 (0·981-1·106)
Proportion of households with adequate water (%)	0·934*** (0·909-0·959)	1·040 (0·995-1·086)	0·582*** (0·483-0·701)	1·056* (1·013-1·100)
Proportion of individuals older than 15 years who are illiterate (%)	1·036*** (1·005-1·067)	1·244*** (1·186-1·304)	1·329*** (1·199-1·472)	1·062*** (1·015-1·112)
Proportion of individuals in poverty (%)	1·228*** (1·199-1·256)	1·415*** (1·360-1·473)	1·532*** (1·301-1·804)	0·980 (0·904-1·062)
GINI index	1·147*** (1·119-1·175)	1·089*** (1·026-1·156)	1·093* (0·953-1·253)	1·039*** (1·012-1·068)
Hospital bed rate (per 1,000 inhabitants)	1·046* (1·011-1·081)	0·908*** (0·844-0·976)	0·891*** (0·802-0·991)	0·969* (0·924-1·016)
Physicians rate (per 10,000 inhabitants)	0·959** (0·936-0·983)	0·849** (0·789-0·915)	0·919** (0·845-0·999)	1·035 (0·995-1·076)
Years binaries	Yes	Yes	Yes	Yes
**Number of observations**	70,476	10,339	2,973	18,812
**Number of municipalities**	3,524	663	149	1,026

Note: Data are in Rate Ratio (RR) coefficients (95% CI) unless otherwise specified. The confidence intervals are in parentheses. Time shocks are controls for specific years of economic crisis (2007, 2008, 2015, and 2018) and for specific years related to PHC (2004-2008). The symbols '***' , '**' and '*' denote significance at 1%, 5%, and 10% respectively.

TABLE 14. Fixed-effect negative for association between Primary Health Care coverage and infant mortality rate by countries, in BCEM, 2000-2019.

Variables	Infant mortality (0-1 years)			
	Brazil	Colombia	Ecuador	Mexico
**Primary Health Care coverage (%)**				
Low (0 to <34·3%)	1·00	1·00	1·00	1·00
Intermediate (≥34·3 to <70·3%)	0·808*** (0·801-0·815)	0·813*** (0·799-0·828)	0·928*** (0·890-0·968)	0·896*** (0·887-0·906)
High (≥70·3% to <100%)	0·799*** (0·787-0·808)	0·706*** (0·687-0·726)	0·830*** (0·791-0·870)	0·926** (0·908-0·944)
Consolidate (100%)	0·802** (0·789-0·814)	0·642*** (0·616-0·668)	0·967* (0·911-1·027)	1·003 (0·969-1·038)
**Control variables**				
Proportion of households with adequate sanitation (%)	0·893*** (0·882-0·904)	1·115* (1·086-1·145)	0·859* (0·733-1·007)	1·028 (0·989-1·070)
Proportion of households with adequate water (%)	0·905*** (0·892-0·917)	0·966** (0·947-0·986)	0·640*** (0·555-0·737)	1·070* (1·046-1·094)
Proportion of individuals older than 15 years who are illiterate (%)	1·105*** (1·088-1·122)	1·197*** (1·171-1·224)	1·052* (0·973-1·137)	1·098*** (1·072-1·124)
Proportion of individuals in poverty (%)	1·235*** (1·220-1·250)	1·374*** (1·351-1·398)	1·050* (0·950-1-161)	1·157*** (1·110-1·206)
GINI index	1·070*** (1·056-1·083)	1·015*** (0·987-1·043)	1·001* (0·901-1·111)	1·089*** (1·074-1·104)
Hospital bed rate (per 1,000 inhabitants)	0·973*** (0·957-0·990)	0·885*** (0·853-0·917)	0·875*** (0·804-0·952)	0·991* (0·967-1·015)
Physicians rate (per 10,000 inhabitants)	0·933*** (0·921-0·945)	0·872*** (0·841-0·903)	0·927*** (0·867-0·991)	1·040* (1·018-1·062)
Years binaries	Yes	Yes	Yes	Yes
**Number of observations**	73,335	11,192	2,973	19,044
**Number of municipalities**	3,667	694	149	1,065

Note: Data are in Rate Ratio (RR) coefficients (95% CI) unless otherwise specified. The confidence intervals are in parentheses. Time shocks are controls for specific years of economic crisis (2007, 2008, 2015, and 2018) and for specific years related to PHC (2004-2008). The symbols '***' , '**' and '*' denote significance at 1%, 5%, and 10% respectively.

TABLE 15. Fixed-effect negative binomial models for the association between Primary Health Care coverage and Under-5 mortality rate, using dummies for countries.

Variables	Code for countries	Dummies for each country, withou:			
		Brazil	Colombia	Ecuador	Mexico
**Primary Health Care coverage (%)**					
Low (0 to <34·3%)	1	1	1	1	1
	(1.000–1.000)	(1.000–1.000)	(1.000–1.000)	(1.000–1.000)	(1.000–1.000)
Intermediate (≥34·3 to <70·3%)	0.882***	0.882***	0.882***	0.882***	0.882***
	(0.877–0.887)	(0.877–0.887)	(0.877–0.887)	(0.877–0.887)	(0.877–0.887)
High (≥70·3% to <100%)	0.838***	0.838***	0.838***	0.838***	0.838***
	(0.832–0.845)	(0.832–0.845)	(0.832–0.845)	(0.832–0.845)	(0.832–0.845)
Consolidate (100%)	0.796***	0.796***	0.796***	0.796***	0.796***
	(0.788–0.805)	(0.788–0.805)	(0.788–0.805)	(0.788–0.805)	(0.788–0.805)
**Control Variables**					
Proportion of households with adequate sanitation (%)	0.900***	0.900***	0.900***	0.900***	0.900***
	(0.892–0.908)	(0.892–0.908)	(0.892–0.908)	(0.892–0.908)	(0.892–0.908)
Proportion of households with adequate water (%)	0.986***	0.986***	0.986***	0.986***	0.986***
	(0.977–0.995)	(0.977–0.995)	(0.977–0.995)	(0.977–0.995)	(0.977–0.995)
Proportion of individuals older than 15 years who are illiterate (%)	1.084***	1.084***	1.084***	1.084***	1.084***
	(1.073–1.094)	(1.073–1.094)	(1.073–1.094)	(1.073–1.094)	(1.073–1.094)
Proportion of individuals in poverty (%)	1.261***	1.261***	1.261***	1.261***	1.261***
	(1.251–1.271)	(1.251–1.271)	(1.251–1.271)	(1.251–1.271)	(1.251–1.271)
Gini Index	1.095***	1.095***	1.095***	1.095***	1.095***
	(1.087–1.104)	(1.087–1.104)	(1.087–1.104)	(1.087–1.104)	(1.087–1.104)
Hospital bed rate (per 1,000 inhabitants)	0.985***	0.985***	0.985***	0.985***	0.985***
	(0.973–0.996)	(0.973–0.996)	(0.973–0.996)	(0.973–0.996)	(0.973–0.996)
Physicians rate (per 10,000 inhabitants)	0.989**	0.989**	0.989**	0.989**	0.989**
	(0.980–0.998)	(0.980–0.998)	(0.980–0.998)	(0.980–0.998)	(0.980–0.998)
**Code for each country**					
Brazil = 1	1	-	-	-	-
	(1.000–1.000)	-	-	-	-
Colombia = 2	0.513***	-	-	-	-
	(0.478–0.551)	-	-	-	-
Ecuador = 3	0.216***	-	-	-	-
	(0.195–0.239)	-	-	-	-
Mexico = 4	0.528***	-	-	-	-
	(0.495–0.563)	-	-	-	-
**Dummies for each country**					
Brazil	-	-	1.950***	4.633***	1.894***
	-	-	(1.816–2.094)	(4.192–5.121)	(1.777–2.020)
Colombia	-	0.513***	-	2.376***	0.972
	-	(0.478–0.551)	-	(2.127–2.654)	(0.897–1.052)
Ecuador	-	0.216***	0.421***	-	0.409***
	-	(0.195–0.239)	(0.377–0.470)	-	(0.368–0.454)
Mexico	-	0.528***	1.029	2.446***	-
	-	(0.495–0.563)	(0.950–1.115)	(2.200–2.718)	-
**Years binaries**	Yes	Yes	Yes	Yes	Yes
**Number of observations**	**109,080**	**109,080**	**109,080**	**109,080**	**109,080**
**Number of municipalities**	**5,576**	**5,576**	**5,576**	**5,576**	**5,576**

Note: Data are in Rate Ratio (RR) coefficients (95% CI) unless otherwise specified. The confidence intervals are in parentheses. Time shocks are control for specific years of economic crisis (2007, 2008, 2015, and 2018) and for specific years related to PHC programs (2004-2008). The symbols '***' , '**' and '*' denote significance at 1%, 5%, and 10% respectively.

TABLE 16. Mixed-effect negative binomial models for the association between Primary Health Care coverage and Under-5 mortality rate, adjusted for random effect for countries.

Variables	Mixed-Effect models	
	Overall	Random-effect for country
**Primary Health Care coverage (%)**		
Low (0 to <34·3%)	1	1
	(1.000–1.000)	(1.000–1.000)
Intermediate (≥34·3 to <70·3%)	0.975***	0.915***
	(0.966–0.984)	(0.912–0.919)
High (≥70·3% to <100%)	0.937***	0.881***
	(0.927–0.946)	(0.877–0.885)
Consolidate (100%)	0.925***	0.856***
	(0.917–0.934)	(0.851–0.861)
**Control Variables**		
Proportion of households with adequate sanitation (%)	0.971***	1.067***
	(0.964–0.979)	(1.063–1.072)
Proportion of households with adequate water (%)	0.972***	0.974***
	(0.965–0.979)	(0.970–0.978)
Proportion of individuals older than 15 years who are illiterate (%)	1.199***	1.134***
	(1.188–1.209)	(1.129–1.140)
Proportion of individuals in poverty (%)	1.136***	1.196***
	(1.126–1.146)	(1.190–1.202)
Gini Index	1.231***	1.108***
	(1.222–1.240)	(1.103–1.113)
Hospital bed rate (per 1,000 inhabitants)	1.058***	1.030***
	(1.050–1.066)	(1.026–1.035)
Physicians rate (per 10,000 inhabitants)	0.932***	0.916***
	(0.925–0.939)	(0.912–0.920)
**Years binaries**	Yes	Yes
**Number of observations**	**109,080**	**109,080**
**Number of municipalities**	**5,576**	**5,576**

Note: Data are in Rate Ratio (RR) coefficients (95% CI) unless otherwise specified. The confidence intervals are in parentheses. Time shocks are control for specific years of economic crisis (2007, 2008, 2015, and 2018) and for specific years related to PHC programs (2004-2008). The symbols '***' , '**' and '*' denote significance at 1%, 5%, and 10% respectively.

TABLE 17. Intraclass Correlation Coefficient (ICC) at municipal, state, and country level

**Country**	**One-Way Absolute agreement**	**Two-Way**	**Mixed**
Individual	-0·397 (-0·402 – -0·392)	-	-0·005 (-0·011 – -0·000)
Average	-1·316 (-1·344 – -1·289)	-	-0·011 (-0·023 – -0·001)
**F test** ICC=0·00	**0·43**	-	**0·99**
**State**	**One-Way**	**Two-Way**	**Mixed**
Individual	-0·217 (-0·222 – -0·211)	-	-0·038 (-0·044 – -0·032)
Average	-0·554 (-0·572 – -0·536)	-	-0·079 (-0·092 – -0·066)
**F test** ICC=0·00	**0·64**	-	**0·93**
**Country and State**	**One-Way**	**Two-Way**	**Mixed**
Individual	-	-0·278 (-0·280 – -0·276)	-0·038 (-0·041 – -0·034)
Average	-	-1·884 (-1·914 – -1·855)	-0·123 (-0·134 – -0·111)
**F test** ICC=0·00	-	**0·35**	**0·89**

Note: The symbols '***' , '**' , '*' and ' ' denote significance at 1%, 5%, 10% and not significant, respectively.

TABLE 18. Multilevel Mixed-effect negative binomial models for the association between Primary Health Care coverage and Under-5 mortality rate, at countries and state levels.

**Variables**	**Negative Binomial**		**Poisson**	
	**Country**	**State**	**Country**	**State**
**Primary Health Care coverage (%)**				
Low (0 to <34·3%)	1 (1·000 – 1·000)	1 (1·000 – 1·000)	1 (1·000 – 1·000)	1 (1·000 – 1·000)
Intermediate (≥34·3 to <70·3%)	0·963*** (0·954 – 0·971)	0·961*** (0·952 – 0·969)	0·915*** (0·912 – 0·919)	0·917*** (0·914 – 0·921)
High (≥70·3% to <100%)	0·913*** (0·904 – 0·922)	0·916*** (0·907 – 0·925)	0·881*** (0·877 – 0·885)	0·884*** (0·880 – 0·889)
Consolidate (100%)	0·881*** (0·872 – 0·890)	0·899*** (0·890 – 0·907)	0·856*** (0·851 – 0·861)	0·892*** (0·887 – 0·898)
**Control Variables**				
Proportion of households with adequate sanitation (%)	1·011*** (1·003 – 1·019)	0·952*** (0·944 – 0·961)	1·067*** (1·063 – 1·072)	0·937*** (0·932 – 0·941)
Proportion of households with adequate water (%)	0·975*** (0·968 – 0·983)	0·976*** (0·969 – 0·983)	0·974*** (0·970 – 0·978)	0·972*** (0·968 – 0·976)
Proportion of individuals older than 15 years who are illiterate (%)	1·160*** (1·150 – 1·171)	1·175*** (1·164 – 1·185)	1·134*** (1·129 – 1·140)	1·145*** (1·139 – 1·151)
Proportion of individuals in poverty (%)	1·214*** (1·203 – 1·226)	1·150*** (1·139 – 1·161)	1·196*** (1·190 – 1·202)	1·169*** (1·163 – 1·174)
Gini Index	1·142*** (1·133 – 1·152)	1·197*** (1·188 – 1·206)	1·108*** (1·103 – 1·113)	1·167*** (1·162 – 1·172)
Hospital bed rate (per 1,000 inhabitants)	1·020*** (1·012 – 1·028)	1·035*** (1·027 – 1·043)	1·030*** (1·026 – 1·035)	1·038*** (1·033 – 1·042)
Physicians rate (per 10,000 inhabitants)	0·930*** (0·922 – 0·937)	0·937*** (0·930 – 0·945)	0·916*** (0·912 – 0·920)	0·950*** (0·946 – 0·955)
Years binaries	Yes	Yes	Yes	Yes
**Number of observations**	109,082	109,082	109,082	109,082
**Number of states (4 countries)**	115	115	115	115
**Number of municipalities**	5.572	5.572	5.572	5.572

Note: Data are in Rate Ratio (RR) coefficients (95% CI) unless otherwise specified. The confidence intervals are in parentheses. Time shocks are control for specific years of economic crisis (2007, 2008, 2015, and 2018) and for specific years related to PHC programs (2004-2008). The symbols '***' , '**' and '*' denote significance at 1%, 5%, and 10% respectively.

TABLE 19. Fixed-effect negative binomial models for the association between Primary Health Care coverage and age-specific mortality rates, using dummies for different types of PHC programs.

Variables	Child<br>Under 5 years	Toddler<br>1 to 4 years	Infant<br>Under 1 year	Post-Neonatal<br>28 days to 1 year	Neonatal<br>0 to 28 days
**Primary Health Care coverage (%)**					
Low (0 to <34·3%)	1	1	1	1	1
	(1·000–1·000)	(1·000–1·000)	(1·000–1·000)	(1·000–1·000)	(1·000–1·000)
Intermediate (≥34·3 to <70·3%)	0·933***	0·972***	0·968***	0·931***	0·917***
	(0·926–0·941)	(0·955–0·988)	(0·959–0·977)	(0·920–0·943)	(0·908–0·927)
High (≥70·3% to <100%)	0·887***	0·874***	0·953***	0·872***	0·892***
	(0·878–0·896)	(0·856–0·893)	(0·942–0·964)	(0·858–0·886)	(0·880–0·903)
Consolidate (100%)	0·850***	0·811***	0·961***	0·781***	0·892***
	(0·839–0·861)	(0·790–0·833)	(0·948–0·975)	(0·765–0·798)	(0·878–0·907)
**Control Variables**					
Proportion of households with adequate sanitation (%)	0·903***	0·917***	0·931***	0·873***	0·919***
	(0·895–0·911)	(0·900–0·935)	(0·921–0·940)	(0·860–0·887)	(0·909–0·930)
Proportion of households with adequate water (%)	0·983***	0·997	0·958***	0·952***	0·999
	(0·974–0·992)	(0·978–1·017)	(0·948–0·967)	(0·937–0·966)	(0·988–1·011)
Proportion of individuals older than 15 years who are illiterate (%)	1·096***	1·126***	1·148***	1·101***	1·081***
	(1·085–1·107)	(1·102–1·150)	(1·136–1·161)	(1·083–1·120)	(1·067–1·094)
Proportion of individuals in poverty (%)	1·241***	1·271***	1·278***	1·244***	1·242***
	(1·230–1·251)	(1·248–1·295)	(1·266–1·290)	(1·226–1·261)	(1·229–1·255)
Gini Index	1·108***	1·103***	1·054***	1·127***	1·096***
	(1·100–1·117)	(1·084–1·122)	(1·044–1·063)	(1·112–1·141)	(1·085–1·106)
Hospital bed rate (per 1,000 inhabitants)	0·984***	0·997	0·953***	1·002	0·980***
	(0·972–0·995)	(0·972–1·021)	(0·941–0·965)	(0·983–1·021)	(0·966–0·994)
Physicians rate (per 10,000 inhabitants)	0·981***	0·964***	0·946***	0·983**	0·996
	(0·972–0·990)	(0·945–0·983)	(0·936–0·956)	(0·968–0·998)	(0·985–1·008)
**PHC type control**					
**Dummy 1 for PHC type** (1= Mexico and Colombia, 0 = Brazil and Ecuador)	1·885***	1·350***	0·441***	0·780***	1·592***
	(1·787–1·988)	(1·130–1·614)	(0·407–0·476)	(0·698–0·870)	(1·468–1·726)
**Interaction dummy with highest PHC coverage**	0·912***	0·860***	0·840***	0·884***	0·946***
	(0·902–0·921)	(0·841–0·879)	(0·830–0·850)	(0·869–0·899)	(0·934–0·958)
Years binaries	Yes	Yes	Yes	Yes	Yes
**Number of observations**	**109,080**	**103,234**	**106,527**	**105,699**	**105,170**
**Number of municipalities**	**5,576**	**5,362**	**5,575**	**5,526**	**5,572**

Note: Data are in Rate Ratio (RR) coefficients (95% CI) unless otherwise specified. The confidence intervals are in parentheses. Time shocks are control for specific years of economic crisis (2007, 2008, 2015, and 2018) and for specific years related to PHC programs (2004-2008). The symbols '***' , '**' and '*' denote significance at 1%, 5%, and 10% respectively.

FIGURE 16. Histogram of the discrete frequency of the occurrence count of the variable of interest.

**(A)**	**(B)**
**Our data**	**A typical zero-inflated situation**

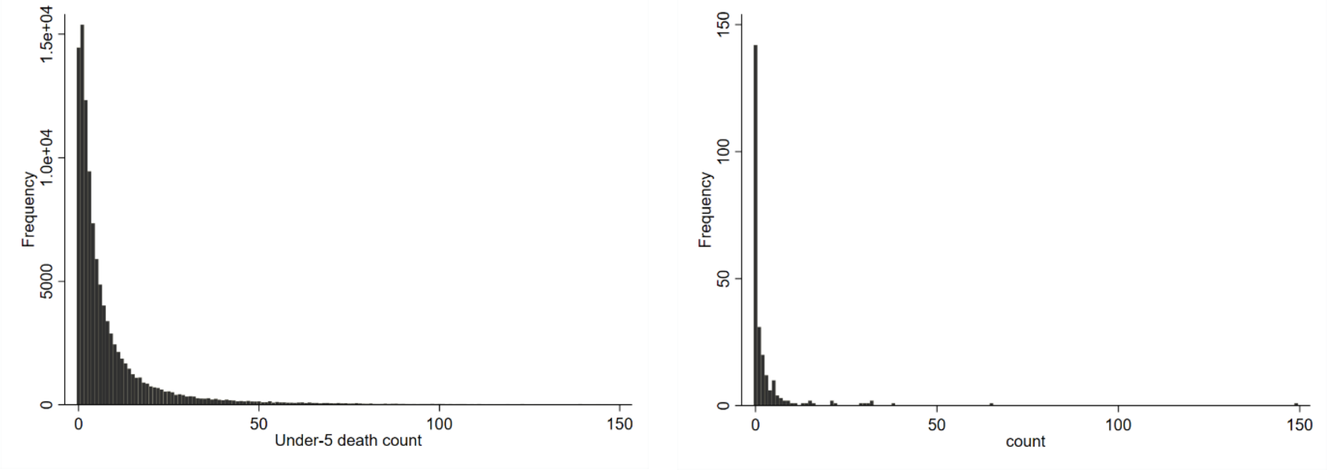

Note: The data presented in Column A refers to the database used in the main model, that is, the count of Under-5 deaths by selected municipalities in Brazil, Colombia, Ecuador and Mexico, during the years 2000-2019. The data presented in Column B are from the STATA database and manual on zero-inflated model, available in <http://www.stata-press.com/data/r10/fish> and < https://stats.oarc.ucla.edu/stata/dae/zero-inflated-negative-binomial-regression>.

TABLE 20. **Frequency, percentage and cumulative percentage of the occurrence count of the variable of interest.**

**Count data**	**(A) Our data (Under-5 death)**			**(B) A typical zero-inflated situation (Count)**		
	**Frequency**	**%**	**% cumulative**	**Frequency**	**%**	**% cumulative**
0	14,461	13.1	13.1	142	56.8	56.8
1	15,388	13.9	27.0	31	12.4	69.2
2	12,335	11.2	38.2	20	8.0	77.2
3 or more	68,227	61.8	100	57	22.8	100

Note: The data presented in Column A refers to the database used in the main model, that is, the count of Under-5 deaths by selected municipalities in Brazil, Colombia, Ecuador and Mexico, during the years 2000-2019. The data presented in Column B are from the STATA database and manual on zero-inflated model, available in <http://www.stata-press.com/data/r10/fish> and < https://stats.oarc.ucla.edu/stata/dae/zero-inflated-negative-binomial-regression>.

TABLE 21. Multilevel Fixed-effect zero-inflated negative binomial models for the association between Primary Health Care coverage and Under-5 mortality rate, using poverty rate and gini index as explanatory variable for the inflation of zero (not occurrence of deaths).

Variables	Poverty rate	Gini Index
**Primary Health Care coverage (%)**		
Low (0 to <34·3%)	1	1
	(1.000,1.000)	(1.000,1.000)
Intermediate (≥34·3 to <70·3%)	0.934***	0.928***
	(0.919,0.948)	(0.914,0.941)
High (≥70·3% to <100%)	0.909***	0.890***
	(0.896,0.923)	(0.878,0.903)
Consolidate (100%)	0.897***	0.905***
	(0.885,0.910)	(0.892,0.917)
**Control Variables**		
Proportion of individuals older than 15 years who are illiterate (%)	1.260***	1.227***
	(1.245-1.274)	(1.209-1.246)
Proportion of households with adequate sanitation (%)	1.009	0.939***
	(0.996-1.021)	(0.926-0.952)
Proportion of households with adequate water (%)	0.928***	0.954***
	(0.917-0.939)	(0.942-0.966)
Proportion of individuals in poverty (%)		1.121***
		(1.099-1.142)
Gini Index	1.170***	
	(1.156-1.184)	
Hospital bed rate (per 1,000 inhabitants)	1.061***	1.113***
	(1.048-1.073)	(1.101-1.125)
Physicians rate (per 10,000 inhabitants)	0.942***	0.945***
	(0.931-0.954)	(0.934-0.956)
Years binaries	Yes	Yes
**Number of observations**	109,082	109,082
**Number of municipalities**	5,572	5,572

Note: "Illiteracy rate" is the abbreviation of "Proportion of individuals older than 15 years who are illiterate". "Sewage" is the abbreviation of "Proportion of households with adequate sanitation". Data are in Rate Ratio (RR) coefficients (95% CI) unless otherwise specified. The confidence intervals are in parentheses. Time shocks are control for specific years of economic crisis (2007, 2008, 2015, and 2018) and for specific years related to PHC programs (2004-2008). The symbols '***' , '**' and '*' denote significance at 1%, 5%, and 10% respectively.

TABLE 22. Results of the regression effects model for the relationship between Primary Health Care coverage and under 5 mortality rate, 2000–2019.

	Data in level (95% IC)		Data in Rate Ratio (RR) (95% IC)
	Model 1	Model 2	Model 3
PHC logarithm $(PHC_{m,t})$	-0.67	-	-
PHC in quartiles	-		
Intermediate (≥34·3 to <70·3%)	-	-0.058*** (-0.069 – -0.047)	0.943*** (0.933 – 0.954)
High (≥70·3% to <100%)	-	-0.104*** (-0.116 – -0.091)	0.901*** (0.890 – 0.913)
Consolidate (100%)	-	-0.137*** (-0.153 – -0.122)	0.872*** (0.858 – 0.885)
Mills ratio $(\lambda_{m,t})$	-2.085 (-2.207 – -1.962)	-1.723 (-1.855 – -1.598)	0.178 (0.156 – 0.202)
Observations	94,322	94,322	94,322
Municipalities	5,578	5,578	5,578
σ_u	0.449	0.432	0.432
σ_e	0.479	0.484	0.484
ρ	0.467	0.443	0.443

Note: Data are in Rate Ratio (RR) coefficients (95% CI) unless otherwise specified. The confidence intervals are in parentheses. The symbols '***' , '**' and '*' denote significance at 1%, 5%, and 10% respectively. σ_u is the variance, σ_e is the standard deviation and ρ is the proportion of the total variance contributed by the panel-level variance component.

TABLE 23. Fixed-effect negative binomial models for the association between Primary Health Care coverage and age-specific mortality rates in Brazil, using dummies for vaccination, prenatal care, and Conditional Cash Transference Programs coverages.

Variables	Child	Toddler	Infant	Post-Neonatal	Neonatal
	Under 5 years	1 to 4 years	Under 1 year	28 days to 1 year	0 to 28 days
**Primary Health Care coverage (%)**					
Low (0 to <34·3%)	1 (1.000 – 1.000)	1 (1.000 – 1.000)	1 (1.000 – 1.000)	1 (1.000 – 1.000)	1 (1.000 – 1.000)
Intermediate (≥34·3 to <70·3%)	0.884*** (0.878 – 0.891)	0.869*** (0.854 – 0.885)	0.839*** (0.832 – 0.847)	0.862*** (0.851 – 0.874)	0.900*** (0.892 – 0.908)
High (≥70·3% to <100%)	0.844*** (0.835 – 0.853)	0.833*** (0.812 – 0.855)	0.849*** (0.838 – 0.860)	0.808*** (0.792 – 0.824)	0.865*** (0.854 – 0.877)
Consolidate (100%)	0.810*** (0.799 – 0.820)	0.789*** (0.765 – 0.814)	0.873*** (0.859 – 0.887)	0.726*** (0.709 – 0.744)	0.860*** (0.845 – 0.874)
**Control Variables**					
Proportion of households with adequate sanitation (%)	0.898*** (0.889 – 0.907)	0.886*** (0.865 – 0.907)	0.933*** (0.922 – 0.945)	0.880*** (0.864 – 0.897)	0.913*** (0.901 – 0.925)
Proportion of households with adequate water (%)	0.959*** (0.948 – 0.970)	0.941*** (0.917 – 0.967)	0.912*** (0.900 – 0.925)	0.905*** (0.887 – 0.924)	0.993 (0.978 – 1.007)
Proportion of individuals older than 15 years who are illiterate (%)	1.016** (1.003 – 1.029)	1.023 (0.992 – 1.054)	1.095*** (1.078 – 1.112)	1.011 (0.987 – 1.034)	1.020** (1.004 – 1.037)
Proportion of individuals in poverty (%)	1.110*** (1.098 – 1.121)	1.131*** (1.104 – 1.159)	1.136*** (1.122 – 1.151)	1.055*** (1.036 – 1.075)	1.136*** (1.121 – 1.151)
Gini Index	1.116*** (1.104 – 1.127)	1.139*** (1.111 – 1.168)	1.068*** (1.055 – 1.081)	1.133*** (1.112 – 1.155)	1.103*** (1.088 – 1.117)
Hospital bed rate (per 1,000 inhabitants)	1.002 (0.988 – 1.016)	1.025 (0.991 – 1.060)	0.956*** (0.940 – 0.972)	1.012 (0.987 – 1.038)	0.99 (0.972 – 1.008)
Physicians rate (per 10,000 inhabitants)	0.980*** (0.969 – 0.990)	0.953*** (0.930 – 0.977)	0.925*** (0.914 – 0.937)	0.964*** (0.946 – 0.983)	0.995 (0.982 – 1.009)
Vaccination coverage (%)	0.997 (0.991 – 1.002)	0.992 (0.979 – 1.005)	0.979*** (0.972 – 0.985)	0.994 (0.984 – 1.004)	1.001 (0.994 – 1.008)

Variables	Child	Toddler	Infant	Post-Neonatal	Neonatal
	**Under 5 years**	**1 to 4 years**	**Under 1 year**	**28 days to 1 year**	**0 to 28 days**
Prenatal care (%)	0.907***	0.918***	0.907***	0.888***	0.913***
	(0.900 – 0.913)	(0.902 – 0.934)	(0.899 – 0.915)	(0.876 – 0.900)	(0.905 – 0.921)
Conditional Cash Transference coverage (%)	0.845***	0.831***	0.825***	0.781***	0.885***
	(0.838 – 0.852)	(0.815 – 0.848)	(0.816 – 0.833)	(0.769 – 0.793)	(0.876 – 0.895)
Years binaries	Yes	Yes	Yes	Yes	Yes
**Number of observations**	**73,358**	**70,476**	**73,335**	**72,536**	**73,295**
**Number of municipalities**	**3,668**	**3,524**	**3,667**	**3,627**	**3,665**

Note: Data are in Rate Ratio (RR) coefficients (95% CI) unless otherwise specified. The confidence intervals are in parentheses. Time shocks are control for specific years of economic crisis (2007, 2008, 2015, and 2018) and for specific years related to PHC programs (2004-2008). The symbols '***' , '**' and '*' denote significance at 1%, 5%, and 10% respectively.

TABLE 24. Models for the Bivariate Association Between Primary Health Care Program (PHC) Level of coverage and Primary Care Indicators: Brazil, 2000–2019.

Variables	Primary Care Indicators	
	**Vaccination**	**Prenatal care**
**Primary Health Care coverage (%)**		
Low (0 to <34·3%)	1	1
	(1.000 – 1.000)	(1.000 – 1.000)
Intermediate (≥34·3 to <70·3%)	1.077***	1.194***
	(1.041 – 1.113)	(1.155 – 1.234)
High (≥70·3% to <100%)	1.201***	1.401***
	(1.163 – 1.241)	(1.357 – 1.446)
Consolidate (100%)	1.295***	1.519***
	(1.263 – 1.329)	(1.481 – 1.558)
**Number of observations**	**73,378**	**73,378**
**Number of municipalities**	**3,669**	**3,669**

Note: Data are in Rate Ratio (RR) coefficients (95% CI) unless otherwise specified. The confidence intervals are in parentheses. The symbols '***' , '**' and '*' denote significance at 1%, 5%, and 10% respectively.

TABLE 25. Fixed-effect negative binomial models for the association between Primary Health Care coverage and age-specific mortality rates, using dummies Conditional Cash Transference Programs.

Variables	Child<br>Under 5 years	Toddler<br>1 to 4 years	Infant<br>Under 1 year	Post-Neonatal<br>28 days to 1 year	Neonatal<br>0 to 28 days
**Primary Health Care coverage (%)**					
Low (0 to <34·3%)	1	1	1	1	1
	(1·000 – 1·000)	(1·000 – 1·000)	(1·000 – 1·000)	(1·000 – 1·000)	(1·000 – 1·000)
Intermediate (≥34·3 to <70·3%)	0·893***	0·904***	0·884***	0·887***	0·892***
	(0·888 – 0·898)	(0·894 – 0·915)	(0·879 – 0·890)	(0·879 – 0·895)	(0·886 – 0·898)
High (≥70·3% to <100%)	0·850***	0·813***	0·872***	0·834***	0·868***
	(0·843 – 0·857)	(0·799 – 0·827)	(0·864 – 0·880)	(0·822 – 0·845)	(0·859 – 0·877)
Consolidate (100%)	0·834***	0·764***	0·880***	0·765***	0·877***
	(0·825 – 0·843)	(0·747 – 0·782)	(0·869 – 0·891)	(0·751 – 0·779)	(0·865 – 0·889)
**Control Variables**					
**Conditional Cash Transference coverage (%)**	0·875***	0·851***	0·865***	0·778***	0·934***
	(0·868 – 0·882)	(0·836 – 0·866)	(0·857 – 0·873)	(0·767 – 0·789)	(0·924 – 0·944)
Proportion of households with adequate sanitation (%)	0·932***	0·947***	0·963***	0·927***	0·933***
	(0·923 – 0·940)	(0·928 – 0·966)	(0·952 – 0·973)	(0·913 – 0·942)	(0·922 – 0·944)
Proportion of households with adequate water (%)	0·999	1·017*	0·967***	0·971***	1·008
	(0·990 – 1·008)	(0·997 – 1·037)	(0·957 – 0·977)	(0·956 – 0·985)	(0·997 – 1·020)
Proportion of individuals older than 15 years who are illiterate (%)	1·094***	1·108***	1·130***	1·087***	1·077***
	(1·083 – 1·105)	(1·085 – 1·132)	(1·117 – 1·143)	(1·069 – 1·105)	(1·064 – 1·091)
Proportion of individuals in poverty (%)	1·226***	1·259***	1·290***	1·216***	1·232***
	(1·216 – 1·236)	(1·236 – 1·283)	(1·277 – 1·302)	(1·199 – 1·233)	(1·219 – 1·245)
Gini Index	1·103***	1·086***	1·029***	1·112***	1·092***
	(1·094 – 1·111)	(1·068 – 1·105)	(1·020 – 1·038)	(1·098 – 1·126)	(1·082 – 1·102)
Hospital bed rate (per 1,000 inhabitants)	0·987**	0·995	0·936***	0·993	0·983**
	(0·976 – 0·998)	(0·971 – 1·020)	(0·924 – 0·948)	(0·974 – 1·012)	(0·969 – 0·997)
Physicians rate (per 10,000 inhabitants)	0·992*	0·975**	0·940***	0·988	1·003
	(0·983 – 1·001)	(0·956 – 0·994)	(0·930 – 0·949)	(0·973 – 1·003)	(0·991 – 1·014)
Years binaries	Yes	Yes	Yes	Yes	Yes
**Number of observations**	**109,080**	**103,234**	**106,527**	**105,699**	**105,170**
**Number of municipalities**	**5,576**	**5,363**	**5,575**	**5,526**	**5,572**

Note: Data are in Rate Ratio (RR) coefficients (95% CI) unless otherwise specified. The confidence intervals are in parentheses. Time shocks are control for specific years of economic crisis (2007, 2008, 2015, and 2018) and for specific years related to PHC programs (2004-2008). The symbols '***' , '**' and '*' denote significance at 1%, 5%, and 10% respectively.

FIGURE 17. Common Support of PHC coverage for the IPTW Method.

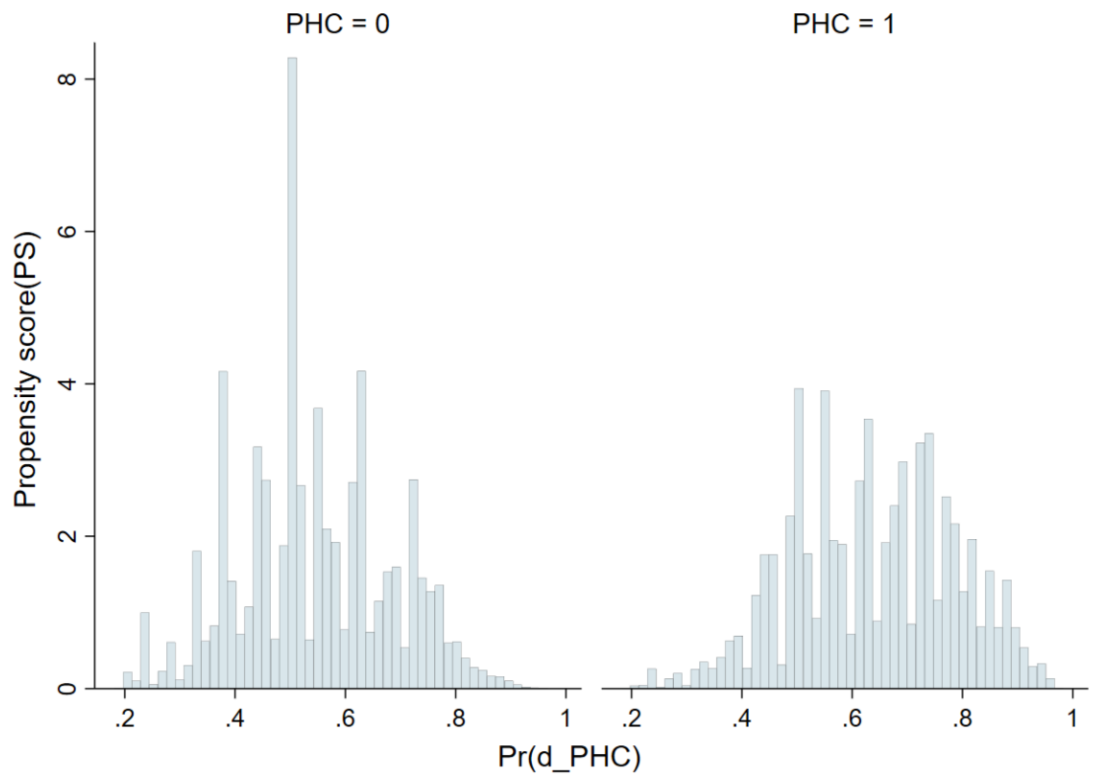

TABLE 26. Mixed-effect negative binomial models for the association between Primary Health Care coverage and Under-5 mortality rates, using a structural nested model and the inverse probability of treatment weight (IPTW).

Variables	Negative Binomial	
	**Country**	**State**
**Primary Health Care coverage (%)**		
Low (0 to <34·3%)	1	1
	(1.000-1.000)	(1.000-1.000)
Intermediate (≥34·3 to <70·3%)	0.963***	0.961**
	(0.937-0.990)	(0.924-1.000)
High (≥70·3% to <100%)	0.923***	0.931***
	(0.914-0.931)	(0.891-0.973)
Consolidate (100%)	0.896***	0.918***
	(0.832-0.965)	(0.867-0.970)
**Control Variables**		
Proportion of households with adequate sanitation (%)	1.015	0.961*
	(0.979-1.053)	(0.918-1.006)
Proportion of households with adequate water (%)	0.981	0.981
	(0.895-1.075)	(0.948-1.014)
Proportion of individuals older than 15 years who are illiterate (%)	1.154***	1.165***
	(1.092-1.219)	(1.130-1.202)
Proportion of individuals in poverty (%)	1.224***	1.143***
	(1.133-1.322)	(1.102-1.186)
Gini Index	1.142***	1.204***
	(1.079-1.208)	(1.152-1.258)
Hospital bed rate (per 1,000 inhabitants)	1.017	1.036**
	(0.981-1.055)	(1.006-1.068)
Physicians rate (per 10,000 inhabitants)	0.932**	0.935***
	(0.882-0.984)	(0.906-0.964)
_cons	0.0135***	0.0149***
	(0.011-0.016)	(0.014-0.016)
/		
lnalpha	0.108***	0.0990***
	(0.065-0.181)	(0.080-0.122)
var(_cons[~)	1.022	
	(0.991-1.053)	
var(_cons[~)		1.022***
		(1.013-1.032)
Years binaries	Yes	Yes
**Number of observations**	**108,388**	**108,388**
**Number of municipalities**	**5,576**	**5,576**
**Number of states**	**115**	**115**
**Number of countries**	**4**	**4**
**Number of Groups**	**4**	**50**

Note: Data are in Rate Ratio (RR) coefficients (95% CI) unless otherwise specified. The confidence intervals are in parentheses. Time shocks are control for specific years of economic crisis (2007, 2008, 2015, and 2018) and for specific years related to PHC programs (2004-2008). The symbols '***' , '**' and '*' denote significance at 1%, 5%, and 10% respectively.

TABLE 27. Fixed-effect negative binomial models for the association between Primary Health Care coverage and Under-5 mortality rate, according to different decades: 2000-2010 and 2010-2019.

Variables	2000-2010 period		2010-2019 period	
**Primary Health Care coverage (%)**				
Low	1.000	1.000	1.000	1.000
Intermediate	0.860***<br>(0.854-0.866)	0.899***<br>(0.892-0.906)	0.959***<br>(0.947-0.970)	0.963***<br>(0.951-0.975)
High	0.796***<br>(0.786-0.805)	0.849***<br>(0.839-0.859)	0.943***<br>(0.927-0.959)	0.952***<br>(0.936-0.968)
Consolidate	0.728***<br>(0.717-0.740)	0.799***<br>(0.787-0.812)	0.943***<br>(0.924-0.963)	0.961***<br>(0.941-0.983)
**Control variables**				
Proportion of households with adequate sanitation (%)		0.915**<br>(0.903-0.927)		0.974**<br>(0.957-0.991)
Proportion of households with adequate water (%)		0.984**<br>(0.969-0.998)		0.998*<br>(0.983-1.013)
Proportion of individuals older than 15 years who are illiterate (%)		1.05***<br>(1.042-1.072)		1.067***<br>(1.046-1.087)
Proportion of individuals in poverty (%)		1.149***<br>(1.137-1.162)		1.049***<br>(1.028-1.071)
GINI index		1.056***<br>(1.045-1.068)		1.041***<br>(1.025-1.058)
Hospital bed rate (per 1,000 inhabitants)		0.988***<br>(0.971-1.005)		1.011<br>(0.993-1.029)
Physicians rate (per 10,000 inhabitants)		0.995*<br>(0.982-1.009)		0.986*<br>(0.971-0.999)
Years binaries	No	Yes	No	Yes
**Number of observations**	**60,681**	**59,972**	**54,955**	**54,297**
**Number of counties**	**5,629**	**5,562**	**5,614**	**5,548**

Note: Data are in Rate Ratio (RR) coefficients (95% CI) unless otherwise specified. The confidence intervals are in parentheses. Time shocks are control for specific years of economic crisis (2007, 2008, 2015, and 2018) and for specific years related to PHC programs (2004-2008). The symbols '***' , '**' and '*' denote significance at 1%, 5%, and 10% respectively.

TABLE 28. Fixed-effect negative binomial models for the association between Primary Health Care coverage and age-specific mortality rates, using the median to categorize the PHC coverage.

Variables	Child	Toddler	Infant	Post-Neonatal	Neonatal
	**Under 5 years**	**1 to 4 years**	**Under 1 year**	**28 days to 1 year**	**0 to 28 days**
**Primary Health Care coverage (%)**					
Below the median	1	1	1	1	1
	[1.000,1.000]	[1.000,1.000]	[1.000,1.000]	[1.000,1.000]	[1.000,1.000]
Above the median	0.921***	0.854***	0.953***	0.862***	0.960***
	[0.913,0.928]	[0.841,0.868]	[0.945,0.962]	[0.851,0.873]	[0.950,0.969]
**Control Variables**					
Proportion of households with adequate sanitation (%)	0.887***	0.895***	0.917***	0.849***	0.906***
	[0.878,0.895]	[0.878,0.913]	[0.908,0.927]	[0.836,0.862]	[0.896,0.916]
Proportion of households with adequate water (%)	0.980***	0.993	0.948***	0.944***	0.995
	[0.971,0.989]	[0.974,1.013]	[0.938,0.958]	[0.930,0.959]	[0.984,1.007]
Proportion of individuals older than 15 years who are illiterate (%)	1.136***	1.157***	1.172***	1.133***	1.115***
	[1.124,1.147]	[1.133,1.182]	[1.159,1.186]	[1.114,1.152]	[1.101,1.129]
Proportion of individuals in poverty (%)	1.262***	1.306***	1.331***	1.279***	1.256***
	[1.251,1.272]	[1.282,1.330]	[1.319,1.344]	[1.261,1.298]	[1.243,1.269]
Gini Index	1.143***	1.131***	1.057***	1.157***	1.131***
	[1.134,1.152]	[1.112,1.150]	[1.047,1.066]	[1.143,1.172]	[1.120,1.141]
Hospital bed rate (per 1,000 inhabitants)	0.992	1.004	0.934***	1.005	0.986*
	[0.981,1.004]	[0.979,1.029]	[0.922,0.946]	[0.986,1.024]	[0.972,1.001]
Physicians rate (per 10,000 inhabitants)	0.971***	0.951***	0.919***	0.964***	0.984***
	[0.962,0.980]	[0.933,0.970]	[0.909,0.928]	[0.949,0.979]	[0.973,0.996]
Years binaries	Yes	Yes	Yes	Yes	Yes
**Number of observations**	**109,080**	**103,234**	**106,527**	**105,699**	**105,170**
**Number of municipalities**	**5,576**	**5,362**	**5,575**	**5,526**	**5,572**

Note: Data are in Rate Ratio (RR) coefficients (95% CI) unless otherwise specified. The confidence intervals are in parentheses. Time shocks are control for specific years of economic crisis (2007, 2008, 2015, and 2018) and for specific years related to PHC programs (2004-2008). The symbols '***' , '**' and '*' denote significance at 1%, 5%, and 10% respectively.

TABLE 29. Fixed-effect negative binomial models for the association between Primary Health Care coverage and age-specific mortality rates, using terciles to categorize the PHC coverage.

Variables	Child<br>Under 5 years	Toddler<br>1 to 4 years	Infant<br>Under 1 year	Post-Neonatal<br>28 days to 1 year	Neonatal<br>0 to 28 days
**Primary Health Care coverage**					
1st tercile	1	1	1	1	1
	[1.000,1.000]	[1.000,1.000]	[1.000,1.000]	[1.000,1.000]	[1.000,1.000]
2nd tercile	0.898***	0.852***	0.923***	0.869***	0.923***
	[0.892,0.904]	[0.840,0.864]	[0.916,0.930]	[0.860,0.879]	[0.915,0.931]
3rd tercile	0.852***	0.753***	0.910***	0.750***	0.919***
	[0.843,0.862]	[0.734,0.771]	[0.898,0.922]	[0.735,0.765]	[0.905,0.932]
**Control Variables**					
Proportion of households with adequate sanitation (%)	0.895***	0.908***	0.923***	0.862***	0.912***
	[0.887,0.904]	[0.890,0.926]	[0.914,0.933]	[0.849,0.875]	[0.902,0.922]
Proportion of households with adequate water (%)	0.987***	1.004	0.954***	0.953***	1.001
	[0.978,0.996]	[0.985,1.024]	[0.944,0.964]	[0.938,0.967]	[0.990,1.013]
Proportion of individuals older than 15 years who are illiterate (%)	1.115***	1.128***	1.157***	1.109***	1.099***
	[1.104,1.126]	[1.104,1.152]	[1.143,1.170]	[1.090,1.127]	[1.085,1.113]
Proportion of individuals in poverty (%)	1.259***	1.300***	1.330***	1.273***	1.254***
	[1.249,1.269]	[1.276,1.324]	[1.317,1.343]	[1.256,1.291]	[1.241,1.267]
Gini Index	1.130***	1.110***	1.047***	1.140***	1.120***
	[1.121,1.138]	[1.091,1.128]	[1.038,1.057]	[1.126,1.155]	[1.110,1.131]
Hospital bed rate (per 1,000 inhabitants)	0.994	1.005	0.937***	1.005	0.988
	[0.983,1.006]	[0.980,1.030]	[0.925,0.949]	[0.986,1.025]	[0.974,1.002]
Physicians rate (per 10,000 inhabitants)	0.981***	0.966***	0.926***	0.976***	0.992
	[0.972,0.990]	[0.947,0.985]	[0.916,0.936]	[0.961,0.991]	[0.981,1.004]
Years binaries	Yes	Yes	Yes	Yes	Yes
**Number of observations**	**109,080**	**103,234**	**106,527**	**105,699**	**105,170**
**Number of municipalities**	**5,576**	**5,362**	**5,575**	**5,526**	**5,572**

Note: Data are in Rate Ratio (RR) coefficients (95% CI) unless otherwise specified. The confidence intervals are in parentheses. Time shocks are control for specific years of economic crisis (2007, 2008, 2015, and 2018) and for specific years related to PHC programs (2004-2008). The symbols '***' , '**' and '*' denote significance at 1%, 5%, and 10% respectively.

TABLE 30. Fixed-effect negative binomial models for the association between Primary Health Care coverage and age-specific mortality rates, using quintiles to categorize the PHC coverage.

Variables	Child<br>Under 5 years	Toddler<br>1 to 4 years	Infant<br>Under 1 year	Post-Neonatal<br>28 days to 1 year	Neonatal<br>0 to 28 days
**Primary Health Care coverage (%)**					
1st quintile	1	1	1	1	1
	[1.000,1.000]	[1.000,1.000]	[1.000,1.000]	[1.000,1.000]	[1.000,1.000]
2nd quintile	0.886***	0.896***	0.877***	0.874***	0.888***
	[0.881,0.891]	[0.886,0.907]	[0.871,0.882]	[0.866,0.882]	[0.882,0.894]
3rd quintile	0.841***	0.804***	0.861***	0.815***	0.863***
	[0.834,0.848]	[0.790,0.818]	[0.853,0.870]	[0.804,0.826]	[0.854,0.871]
4th quintile	0.836***	0.782***	0.869***	0.770***	0.878***
	[0.826,0.846]	[0.762,0.802]	[0.857,0.882]	[0.754,0.785]	[0.864,0.891]
5th quintile	0.776***	0.690***	0.830***	0.660***	0.844***
	[0.766,0.787]	[0.671,0.710]	[0.817,0.842]	[0.645,0.675]	[0.830,0.859]
**Control Variables**					
Proportion of households with adequate sanitation (%)	0.901***	0.913***	0.926***	0.870***	0.917***
	[0.893,0.909]	[0.896,0.931]	[0.916,0.935]	[0.857,0.883]	[0.907,0.928]
Proportion of households with adequate water (%)	0.991*	1.007	0.959***	0.957***	1.004
	[0.982,1.000]	[0.987,1.027]	[0.949,0.969]	[0.943,0.972]	[0.992,1.015]
Proportion of individuals older than 15 years who are illiterate (%)	1.096***	1.116***	1.129***	1.091***	1.079***
	[1.085,1.107]	[1.093,1.140]	[1.117,1.142]	[1.073,1.109]	[1.066,1.093]
Proportion of individuals in poverty (%)	1.252***	1.294***	1.319***	1.264***	1.247***
	[1.242,1.262]	[1.271,1.318]	[1.307,1.331]	[1.246,1.282]	[1.234,1.260]
Gini Index	1.100***	1.085***	1.026***	1.108***	1.091***
	[1.092,1.109]	[1.067,1.103]	[1.017,1.036]	[1.094,1.122]	[1.080,1.101]
Hospital bed rate (per 1,000 inhabitants)	0.993	1.002	0.943***	1.002	0.985**
	[0.982,1.004]	[0.977,1.027]	[0.930,0.955]	[0.983,1.021]	[0.971,1.000]
Physicians rate (per 10,000 inhabitants)	0.992*	0.974***	0.940***	0.987*	1.002
	[0.983,1.001]	[0.955,0.993]	[0.930,0.950]	[0.972,1.002]	[0.991,1.014]
Years binaries	Yes	Yes	Yes	Yes	Yes
**Number of observations**	**109,080**	**103,234**	**106,527**	**105,699**	**105,170**
**Number of municipalities**	**5,576**	**5,362**	**5,575**	**5,526**	**5,572**

Note: Data are in Rate Ratio (RR) coefficients (95% CI) unless otherwise specified. The confidence intervals are in parentheses. Time shocks are control for specific years of economic crisis (2007, 2008, 2015, and 2018) and for specific years related to PHC programs (2004-2008). The symbols '***' , '**' and '*' denote significance at 1%, 5%, and 10% respectively.

TABLE 31. Random Spatial effect models with Neighbors-based correlations functions at municipal levels for association between Primary Health Care coverage and age-specific mortality rates in Brazil, 2000–2019.

	CAR	ICAR
**Primary Health Care coverage (%)**		
Low (>=0 to <38.9)	1	1
Intermediate (≥38.9 to <75.9%)	0.896***(0.886,0.905)	0.886***(0.877,0.896)
High (≥75·9% to <100%)	0.844***(0.834,0.855)	0.833***(0.823,0.844)
Consolidate (100%)	0.788***(0.778,0.798)	0.777***(0.767,0.788)
**Control Variables**		
Proportion of households with adequate sanitation (%)	0.943***(0.933,0.953)	0.886***(0.875,0.896)
Proportion of households with adequate water (%)	0.895***(0.885,0.905)	0.895***(0.885,0.906)
Proportion of individuals older than 15 years who are illiterate (%)	1.152***(1.137,1.167)	1.093***(1.077,1.108)
Proportion of individuals in poverty (%)	1.224***(1.209,1.238)	1.185***(1.17,1.199)
Gini Index	1.135***(1.123,1.147)	1.117***(1.105,1.13)
Hospital bed rate (per 1,000 inhabitants)	1.008 (0.996,1.02)	1.002 (0.99,1.014)
Physicians rate (per 10,000 inhabitants)	0.972***(0.962,0.982)	0.977***(0.967,0.987)
**Years (interventions)**		
2007	1.01 (0.995,1.026)	1.011 (0.996,1.026)
2008	0.992 (0.977,1.007)	0.995 (0.98,1.01)
2015	0.886***(0.872,0.901)	0.884***(0.869,0.899)
2018	0.869***(0.854,0.883)	0.867***(0.853,0.882)

Note: Neighbors based correlations functions (Random spatial effect). ICAR: Intrinsic Conditional Autorregressive Correlation. CAR: Conditional Autorregresive Correlation

5. Deaths averted by PHC programs during 2000-19

To simulate deaths avoided due to PHC strategies in 2000-19 period, we predicted coefficient $E(Y_{it} \mid X)$, here X represents the set of covariates including the interventions, and Y_{it} are the real under-5 mortality rate at municipality *i*, in year *t*, using the Monte Carlo method to obtain uncertainty intervals. It can be summarized in the following steps:

1. Predict the number of deaths NDY_{it} using the same coefficients of the main model (Table 2 in manuscript) and the same values of the variables of the retrospective dataset (2000-2019), except for PHC that is fixed to 0 in all municipalities and all years.
2. Calculate the cumulative difference between the real deaths DY_{it} and NDY_{it} over the years 2000-2019.

For each outcome, 10,000 simulations were performed. The number 10,000 was chosen based on the observed stabilization of the estimates.

According to this method, we estimated that the implementation of PHC programs in the BCEM countries avoided 305,890 (95%CI: 251,826-360,517) child deaths over the period 2000-19 in the hypothetical case these programs did not exist (0% coverage).

TABLE 31. Child death avoided by Primary Health Care (PHC) coverage during 2000-19.

**Averted deaths until 2019**		
Estimate	LI	LS
305,890	251,826	360,517

PART IV – FORECASTING ANALYSIS

6. Description of the forecasting methodology

This section gives guidelines about the forecasting procedure. This process was developed according to standard international modeling reporting guidelines (ISPOR-SMDM). The modeling approach adopted for this study was developed based on two stages.

First, a synthetic set of control variables were simulated for Brazilian, Colombian, Ecuadorian and Mexican municipalities for the period 2010 – 2030. This was done using the available municipality information for the period 2010 -2019 (see Table 1). On the other hand, poverty, Gini index, and the exposure PHC were used to create different economic scenarios as well as policy responses.

In the second stage, for each year and each municipality, the mortality rate for all the municipalities was estimated as the outcome of the same multivariate fixed effects regressions, using the forecast demographic, socioeconomic and exposure variables (PHC coverage) as input values.

7. Purpose of the forecasting and its applications

The developed model had the overall purpose to simulate the effects of socioeconomic and policy coverage changes on health outcomes in BEM countries using ecologic-level data and - when available - retrospective ecologic datasets. Elements of flexibility have been introduced in the code to allow simulation of different sets of variables and different regression models.

The purpose of forecasting methodologies in this context, is to simulate the effects of PHC policies on health outcomes within BCEM municipalities, this is done using information from retrospective ecological datasets. R code has been developed to allow flexible simulation of different sets of variables and different regression models.

8. Inputs, outputs, and other parameters

8.1. Scenarios of poverty and coverage of PHC programs

In order to develop forecasting, exponential functions were used to simulate the covariates behavior for the next 11 years (2020-2030). Regarding the poverty rate, an increasing scenario was considered for the first years (economic crisis period). This is described by the equation,

$$x_t = x_{2019} + c_1 x_{2019}(1 - \exp(-k_1 t)), \; (2)$$

for the remaining years, we consider the exponential decay,

$$x_t = x_{2019} - c_2 x_{2019}(1 - \exp(-k_2 t)), \; (3)$$

where the parameters c_1, k_1, c_2, k_2 were settled according to different available sources.

With respect to the intervention variables (PHC) under the mitigation scenario, they were considered as having the same behavior of the poverty rate (mitigation effect) during the economic crisis.[31] For the post - crisis, the interventions were simulated using the exponential decay in equation (3). The decreasing rate k_2 in this case, was settled as half the poverty to simulate the transition period between the crisis and recovery scenarios.

Under the austerity scenarios, the interventions x_t, austerity were considered to follow an exponential decay which shows directly the percentage of decrease per year, this allowed to support the policies simulation according to situations that concerns the BEM countries government expenditure.[32] The equation below describes this dynamic.

$$x_{t,aust} = x_{t,aust}\,(1-p)^t$$

where p is the percentage of decreasing for each intervention and t refers to the year.

We simulated three economic crisis scenarios using the increase in the poverty rates, which was calculated using the data from 2000 to 2019. Poverty is defined as the percentage of households below the eligibility condition for the PHC program. The magnitude of the economic crisis is represented by the percentage variation of the poverty rate from 2019. Recent reports show that the acute increase of poverty rate from 2020 to 2021 (about 22,7%) is significantly higher compared to the annual poverty increase used as economic crises scenarios in this study.[33]

The economic crisis scenarios considered in this analysis were simulated as follows:

- Shorter Economic Crisis scenario: A milder and shorter economic crisis, with an increase in poverty rates for the first three years (2020 - 2022). This behavior was generated using equation (2). On the other side, For the post-crisis period (2023 -2030), poverty rates were simulated by using equation (3), the parameters describing the poverty by country for this first scenario are described in Web-Table 32.

TABLE 32. Parameters describing poverty during the first economic crisis scenario.

Parameter	Country	Increasing period	Decreasing period
k	Brazil	0·225	0·2
	Colombia	0·166	0·23
	Mexico	0·02	0·03
	Ecuador	0·15	0·3
c	Brazil	0·6	0·53
	Colombia	0·4	0·2
	Mexico	0·025	0·05
	Ecuador	0·2	0·1

- Medium Economic Crisis scenario: A medium economic crisis with a larger increase in the poverty rate for the first 5 years (2020-2024). This behavior was generated using equation (2). As in the first scenario, For the post-crisis period (2025 -2030), the poverty rates were simulated by using equation (3), the parameters describing the poverty by country for this scenario are described in Table 33.

TABLE 33. Parameters describing poverty during the second economic crisis scenario.

Parameter	Country	Increasing period	Decreasing period
k	Brazil	0·45	0·2
	Colombia	0·25	0·23
	Mexico	0·04	0·03
	Ecuador	0·3	0·3
c	Brazil	1·2	0·53
	Colombia	0·6	0·2
	Mexico	0·05	0·05
	Ecuador	0·4	0·1

- Longer Economic Crisis scenario: A longer economic crisis was created using similar parameters as the Medium Economic Crisis scenarios, but with an increase sustained over 7 years (from 2015 to 2021).

In response to the economic crisis, three policy responses were considered in the main analysis:

- Mitigation scenario: a mitigation strategy with a proportional behavior of the PHC programs to the poverty scenarios, during the corresponding simulated economic crisis. In this case, these interventions were generated in the same way as poverty rates, considering the same equation and parameters according to each period and scenario.
- Baseline scenario: derived from a validated model - already employed in previous studies[34, 35] - that projected the effects of the current fiscal austerity measures due to the *Emenda Constitucional* 95 (EC95) on the coverage of the three interventions. This scenario was simulated according to the equation (4) considering a percentage of decrease of 5%, as in previous studies.[35, 36]
- Severe Austerity scenario: based on the reduction of PHC proportional to the reduction of government expenditure on social protection observed from 2014-2019.[36] This scenario was simulated according to the equation (4) considering a percentage of decrease of 9·8%. This percentage was derived from the reduction of government expenditure on social protection (excluding cash transfer programs) observed from 2014-2019.[36]

The Figure 18 show the behavior of the poverty scenarios.

FIGURE 18. Forecasting poverty scenarios.

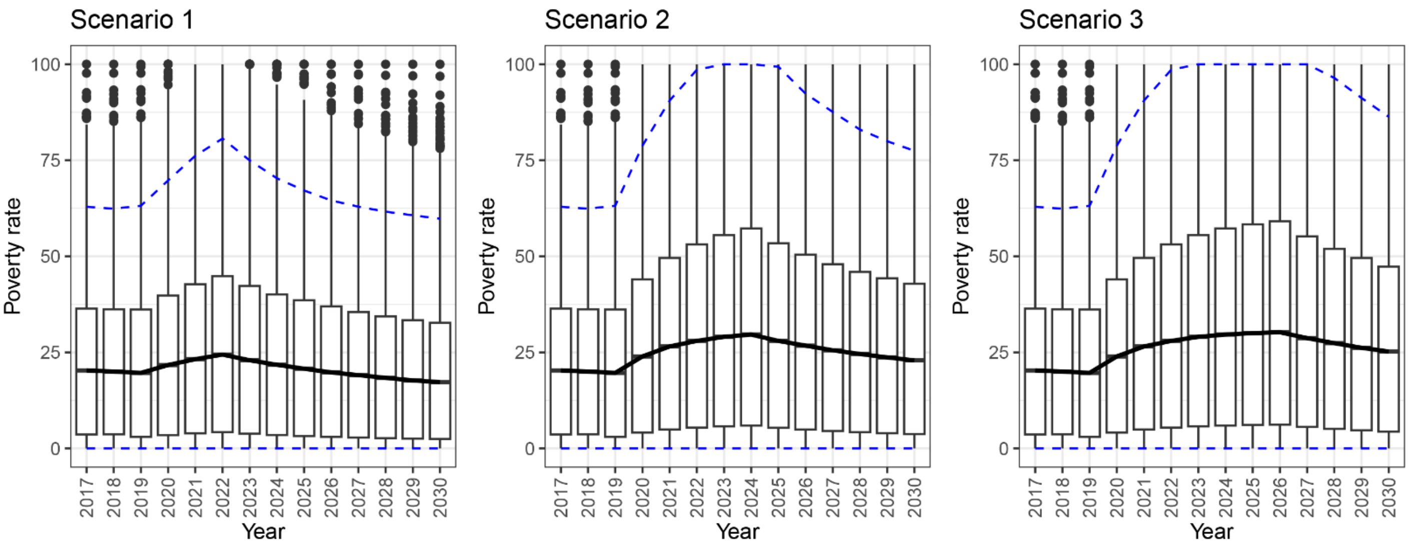

9. Prediction methodology

To generate predictions and confidence intervals for each response Y_{it}, the Monte Carlo methodology was used. This procedure allows to get more accurate results compared with conventional methods such as the use of the normal distribution. It can be summarized in the following steps

Simulate the intervention values for the forecasting period (2020 -2030) using the mitigation and austerity scenarios settled in previous sections. Also simulate the control covariates using equations (2) and (3) and following their trend. Simulate a new Y_{it} from the negative binomial distribution using the estimated parameters from the retrospective study and the forecasted covariates.

Get the predictions $E(Y_{it} \mid X)$ using the new simulated variable Y_{it}, here X represents the set of covariates including the interventions.
Get back to step 1.

The algorithm ended when the number of desired Monte Carlo simulations M is reached. The predictions and confidence interval estimated for Y_{it} will be the mean and the percentiles 2·5% and 97·5% of the M simulations respectively. For each outcome and each scenario, 10,000 simulations were performed, allowing parameter values to vary in each simulation cycle according to their assumed underlying distribution. The number 10,000 was chosen based on the stabilization of the estimates.

9.1. External validation of each model

The external validation of the model was undertaken comparing the overall national mortality rate (computed for each municipality) forecasted using microsimulations, with the official Brazilian mortality estimates (overall) during the years 2010-2019, which are the most up-to-date available, and estimating the linear regression and the correlation coefficients (R2) of predicted vs observed values, as shown in Figure 19.

FIGURE 19. Linear regression and correlation coefficient (R2) of predicted vs observed values, and trend of the simulated overall mortality rate vs the official Brazilian mortality rate estimates for the period 2010-2019.

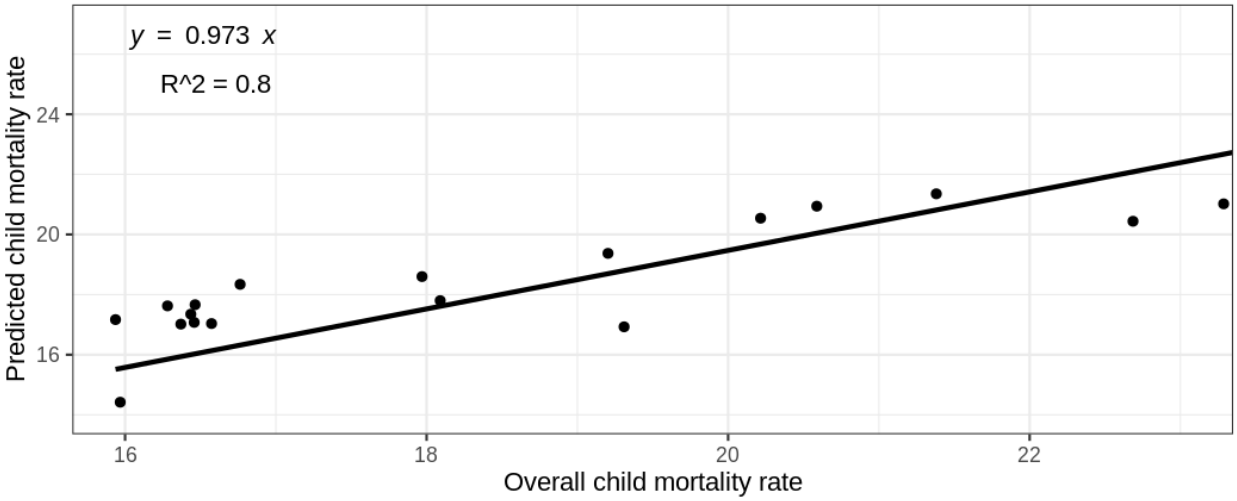

10. Sensitivity analysis

To evaluate how a lengthening of the economic crisis could affect mortality rates we additionally modelled the impact of austerity and social protection mitigation considering two additional poverty scenarios (see Figures 19). The Table 34 is consistent with the results found for the poverty scenario 2 (see the main manuscript) showing that the averted deaths decrease as the policies conditions improve in terms of increase the coverage of the different social programs.

TABLE 34. Rate ratio (RR) and cumulative difference in under-five deaths over the period 2020-2030 between alternative policy scenarios, according to different the Economic Crisis Scenario.

	Mitigation/Baseline=5%		Mitigation/Austerity=9.8%	
**Year**	**Rate Ratio (RR)**	**Li -Ls**	**Rate Ratio (RR)**	**Li -Ls**
		**Under -five Mortality Rate**		
		Poverty scenario 1		
2020	0·950	0·913 – 0·987	0·945	0·909 – 0·983
2025	0·882	0·845 – 0·920	0·852	0·807 – 0·892
2030	0·845	0·801 – 0·892	0·780	0·734 – 0·851
Avoidable deaths	68,054	56,797 – 79,350	105,703	89,243 – 122,177
		Poverty scenario 2		
2020	0·942	0·903 - 0·982	0·938	0·899 – 0·983
2025	0·868	0·828 - 0·910	0·840	0·798 - 0·893
2030	0·833	0·787 – 0·880	0·769	0·724 – 0·841
Avoidable deaths	103,737	86,812 – 120,895	142,284	120,217 - 164,378
		Poverty scenario 3		
2020	0·942	0·903 – 0·982	0·938	0·899 – 0·983
2025	0·867	0·827 - 0·910	0·840	0·797 – 0·898
2030	0·832	0·787 – 0·879	0·769	0·724 – 0·839
Avoidable deaths	108,756	91,056 – 126,647	147,582	124,693 - 170,471

Source: Author's data analysis for municipalities in Brazil, Colombia, Ecuador and Mexico (BCEM countries).
Note: Data are in Rate Ratio (RR) coefficients (95% CI) unless otherwise specified.

11. Main Limitations

Uncertainty around future macroeconomic scenarios in Latin America is one of the main limitations of this work. Specifically, the extremely unstable economic situation creates uncertainty around prediction of poverty rate, income and other independent variables. This left us to simulate several crisis scenarios, creating comparative results between our findings. Another limitation is the protective effects of PHC on overall and childhood morbidity and mortality,[37] this could affect the modelling of austerity measures focused on PHC programs.

On the other hand, estimates of impact austerity measures on under 5 age groups are probably conservative in this work. This is because the non-reflection of constraints in other public spending areas such as housing, education and other welfare programs with known impacts on poverty and health. . Moreover, austerity measures recently enshrined in the constitution of BEM countries means that public spending will only increase in line with inflation, which will not account for the demographic growth of the population, its ageing processes, and growing costs associated with new healthcare treatments and technologies.[36, 38] Another limitation of the study is that we do not model the impact of the increased coverage of PHC on poverty rate dynamics, assuming that poverty rates influence PHC coverage and not the contrary. This is mainly due to the assumption that WB simulations of poverty increase during economic crisis already account for PHC effects, and because reliable parameters were not available at the moment of writing.

REFERENCES

1. Hone T, Rasella D, Barreto M, Atun R, Majeed A, Millett C. Large Reductions In Amenable Mortality Associated With Brazil's Primary Care Expansion And Strong Health Governance. Health Aff (Millwood). 2017;36(1):149-58.
2. de Almeida ER, de Sousa ANA, Brandao CC, de Carvalho FFB, Tavares G, Silva KC. [National primary health care policy in Brazil: an analysis of the review process (2015-2017)Politica Nacional de Atencion Basica en Brasil: un analisis del proceso de revision (2015-2017)]. Rev Panam Salud Publica. 2018;42:e180.
3. Rasella D, Hone T, de Souza LE, Tasca R, Basu S, Millett C. Mortality associated with alternative primary healthcare policies: a nationwide microsimulation modelling study in Brazil. BMC Med. 2019;17(1):82.
4. Rasella D, Aquino R, Barreto ML. Reducing childhood mortality from diarrhea and lower respiratory tract infections in Brazil. Pediatrics. 2010;126(3):e534-40.
5. Rasella D, Harhay MO, Pamponet ML, Aquino R, Barreto ML. Impact of primary health care on mortality from heart and cerebrovascular diseases in Brazil: a nationwide analysis of longitudinal data. BMJ. 2014;349:g4014.
6. Hone T, Rasella D, Barreto ML, Majeed A, Millett C. Association between expansion of primary healthcare and racial inequalities in mortality amenable to primary care in Brazil: A national longitudinal analysis. PLoS Med. 2017;14(5):e1002306.
7. Giedion U, Uribe MV. Colombia's universal health insurance system. Health Aff (Millwood). 2009;28(3):853-63.
8. Torres F, Bernal O. The Subsidized Regime of Colombia’s National Health Insurance System.: World Bank; 2013 Apr. Contract No.: 15.
9. Aldulaimi S, Mora FE. A Primary Care System to Improve Health Care Efficiency: Lessons from Ecuador. J Am Board Fam Med. 2017;30(3):380-3.
10. ISAGS. Sistemas de Salud en Suramérica: desafíos para la universalidad, la integralidad y la equidad. Rio de Janeiro: ISAGS; 2012.
11. Espinosa V, Acuña C, De la Torres D, Tambini G. La reforma en salud del Ecuador. Pan American Journal of Public Health. 2017;41.
12. Garcia-Diaz R, Sosa-Rubi SG, Servan-Mori E, Nigenda G. Welfare effects of health insurance in Mexico: The case of Seguro Popular de Salud. PLoS One. 2018;13(7):e0199876.
13. Hone T, Macinko J, Millett C. Revisiting Alma-Ata: what is the role of primary health care in achieving the Sustainable Development Goals? Lancet. 2018;392(10156):1461-72.
14. Kluge H, Kelley E, Swaminathan S, Yamamoto N, Fisseha S, Theodorakis PN, et al. After Astana: building the economic case for increased investment in primary health care. Lancet. 2018;392(10160):2147-52.
15. Macinko J, Starfield B, Erinosho T. The impact of primary healthcare on population health in low- and middle-income countries. J Ambul Care Manage. 2009;32(2):150-71.
16. Kluge H, Kelley E, Barkley S, Theodorakis PN, Yamamoto N, Tsoy A, et al. How primary health care can make universal health coverage a reality, ensure healthy lives, and promote wellbeing for all. Lancet. 2018;392(10156):1372-4.
17. WHO. The World Health Report 2008-Primary Health Care (Now More Than Ever). Available at: https://apps.who.int/iris/handle/10665/43949.
18. Ford-Gilboe M, Wathen CN, Varcoe C, Herbert C, Jackson BE, Lavoie JG, et al. How Equity-Oriented Health Care Affects Health: Key Mechanisms and Implications for Primary Health Care Practice and Policy. Milbank Q. 2018;96(4):635-71.
19. DeVoe JE, Bazemore AW, Cottrell EK, Likumahuwa-Ackman S, Grandmont J, Spach N, et al. Perspectives in Primary Care: A Conceptual Framework and Path for Integrating Social Determinants of Health Into Primary Care Practice. Ann Fam Med. 2016;14(2):104-8.
20. Jones MK, Bloch G, Pinto AD. A novel income security intervention to address poverty in a primary care setting: a retrospective chart review. BMJ Open. 2017;7(8):e014270.
21. Pega F, Liu SY, Walter S, Pabayo R, Saith R, Lhachimi SK. Unconditional cash transfers for reducing poverty and vulnerabilities: effect on use of health services and health outcomes in low- and middle-income countries. Cochrane Database Syst Rev. 2017;11(11):CD011135.
22. Cates CB, Weisleder A, Mendelsohn AL. Mitigating the Effects of Family Poverty on Early Child Development through Parenting Interventions in Primary Care. Acad Pediatr. 2016;16(3 Suppl):S112-20.
23. Adams J, White M, Moffatt S, Howel D, Mackintosh J. A systematic review of the health, social and financial impacts of welfare rights advice delivered in healthcare settings. BMC Public Health. 2006;6:81.

24. Haighton C, Moffatt S, Howel D, McColl E, Milne E, Deverill M, et al. The Do-Well study: protocol for a randomised controlled trial, economic and qualitative process evaluations of domiciliary welfare rights advice for socio-economically disadvantaged older people recruited via primary health care. BMC Public Health. 2012;12:382.
25. AbouZahr C, de Savigny D, Mikkelsen L, Setel PW, Lozano R, Nichols E, et al. Civil registration and vital statistics: progress in the data revolution for counting and accountability. Lancet. 2015;386(10001):1373-85.
26. Mikkelsen L, Phillips DE, AbouZahr C, Setel PW, de Savigny D, Lozano R, et al. A global assessment of civil registration and vital statistics systems: monitoring data quality and progress. Lancet. 2015;386(10001):1395-406.
27. de Andrade C. Socio-spatial inequalities in the adequacy of Ministry of Health data on births and deaths at the municipal level in Brazil. Cad Saude Publica. 2007;23(5):1207-16.
28. Moncayo AL, Granizo G, Grijalva MJ, Rasella D. Strong effect of Ecuador's conditional cash transfer program on childhood mortality from poverty-related diseases: a nationwide analysis. BMC Public Health. 2019;19(1):1132.
29. Rasella D, Aquino R, Barreto ML. Impact of the Family Health Program on the quality of vital information and reduction of child unattended deaths in Brazil: an ecological longitudinal study. BMC Public Health. 2010;10:380.
30. Lawlor DA, Tilling K, Davey Smith G. Triangulation in aetiological epidemiology. Int J Epidemiol. 2016;45(6):1866-86.
31. Rasella D, Basu S, Hone T, Paes-Sousa R, Ocke-Reis CO, Millett C. Child morbidity and mortality associated with alternative policy responses to the economic crisis in Brazil: A nationwide microsimulation study. PLoS Med. 2018;15(5):e1002570.
32. Mariani CB, Gomes EC, Cenci DR, Queiroz RF. Financiamiento de Asistencia Social no Brasil. Nota Técnica de Monitoreamento. 2019.
33. R. SA, Ribeiro MG. Boletin Desigualdade nas Metrópoles. Porto Alegre: Observatorio das Metrópoles.
34. Fernald LC, Gertler PJ, Neufeld LM. Role of cash in conditional cash transfer programmes for child health, growth, and development: an analysis of Mexico's Oportunidades. Lancet. 2008;371(9615):828-37.
35. Levy S. Progress Against Poverty: Sustaining Mexico's Progresa-Oportunitdades Program. Washington: Brookings Institution Press; 2007.
36. Rossi P, Dweck E. Impacts of the New Fiscal Regime on health and education. Cadernos de Saúde Pública. 2016;32(12).
37. Rasella D, Aquino R, Santos CA, Paes-Sousa R, Barreto ML. Effect of a conditional cash transfer programme on childhood mortality: a nationwide analysis of Brazilian municipalities. Lancet. 2013;382(9886):57-64.
38. Paiva AB, Mesquina ACS, Jaccoud L, Passos L. The new tax regime and its implications for social assistance policy and Brazil. Instituto de Pesquisa Economica Aplicada. 2016;27.
